# Supplementary material for: Gliovascular transcriptional perturbations in Alzheimer’s disease reveal molecular mechanisms of blood brain barrier dysfunction
Source: Nat Commun. 2024 Jun 20;15:4758. doi: 10.1038/s41467-024-48926-6 (PMC11190273; doi:10.1038/s41467-024-48926-6)
Supplement: Supplementary file 1 — Supplementary Information [file 41467_2024_48926_MOESM1_ESM.pdf]

Gliovascular transcriptional perturbations in Alzheimer's disease reveal molecular mechanisms of blood brain barrier dysfunction

Özkan İş, PhD[1]*, Xue Wang, PhD[2]*, Joseph S. Reddy, PhD[2], Yuhao Min, BS[1], Elanur Yilmaz[3,4], PhD, Prabesh Bhattarai, PhD[3,4], Tulsi Patel, PhD[1], Jeremiah Bergman, BS[1], Zachary Quicksall, MS[2], Michael G. Heckman, MS[2], Frederick Q Tutor-New, BA, BS[1], Birsen Can Demirdogen, PhD[1,5], Launia White[2], Shunsuke Koga, MD, PhD[1], Vincent Krause[1], Yasuteru Inoue, MD, PhD[1], Takahisa Kanekiyo, MD, PhD[1], Mehmet Ilyas Cosacak, PhD[6], Nastasia Nelson[3,4], Annie J. Lee, PhD[3,4,7], Badri Vardarajan, PhD[3,4,7], Richard Mayeux, MD, PhD[3,4,7,8,9], Naomi Kouri, PhD[1], Kaancan Deniz, MD[1], Troy Carnwath, BS[1], Stephanie R. Oatman, BS[1],Laura J. Lewis-Tuffin, PhD[10], Thuy Nguyen, BS[1], for the Alzheimer's Disease Neuroimaging Initiative^, Minerva M. Carrasquillo, PhD[1], Jonathan Graff-Radford, MD[11], Ronald C. Petersen, MD, PhD[11,12], Clifford R Jr Jack, MD[13], Kejal Kantarci, MD, MS[12], Melissa E. Murray, PhD[1], Kwangsik Nho, PhD[14,15,16], Andrew J. Saykin, PsyD[,15,16,17], Dennis W. Dickson, MD[1], Caghan Kizil, PhD[3,4,7], Mariet Allen, PhD[1] and Nilüfer Ertekin-Taner, MD, PhD[1,18#]

[1] Department of Neuroscience, Mayo Clinic, Jacksonville, FL, USA
[2] Department of Quantitative Health Sciences, Mayo Clinic, Jacksonville, FL, USA
[3] Department of Neurology, Columbia University Irving Medical Center, New York, NY, USA
[4] Taub Institute for Research on Alzheimer's Disease and the Aging Brain, Columbia University Irving Medical Center, New York, NY, USA
[5] Department of Biomedical Engineering, TOBB University of Economics and Technology, Ankara, Turkey
[6] German Center for Neurodegenerative Diseases (DZNE) within Helmholtz Association, Dresden, Germany
[7] The Gertrude H. Sergievsky Center, College of Physicians and Surgeons, Columbia University, New York, NY, USA
[8] Department of Psychiatry, Columbia University Irving Medical Center, New York, NY, USA
[9] Department of Epidemiology, Mailman School of Public Health, Columbia University, New York, NY, USA
[10] Mayo Clinic Florida Cytometry and Cell Imaging Laboratory, Mayo Clinic, Jacksonville, FL, USA
[11] Department of Neurology, Mayo Clinic, Rochester, MN, USA
[12] Mayo Clinic Alzheimer's Disease Research Center, Rochester, MN, USA
[13] Mayo Clinic, Radiology, Rochester, MN, USA
[14] Center for Computational Biology and Bioinformatics, Indiana University School of Medicine, Indianapolis, IN, USA
[15] Department of Radiology and Imaging Sciences, Indiana University School of Medicine, Indianapolis, IN, USA
[16] Indiana Alzheimer's Disease Research Center, Indiana University School of Medicine, Indianapolis, IN, USA
[17] Department of Medical and Molecular Genetics, Indiana University School of Medicine, Indianapolis, IN, USA
[18] Department of Neurology, Mayo Clinic, Jacksonville, FL, USA
[*] equal contribution
^ A list of authors and their affiliations appears at the end of the paper.
# corresponding author
Corresponding Author address and e-mail:

Mayo Clinic, 4500 San Pablo Road, Jacksonville, FL 32224. E-mail: taner.nilufer@mayo.edu

Supplementary Table of Abbreviations

Abbreviations:

Aβ: Amyloid β
AD: Alzheimer's disease
AG: Angular gyrus
Ast: Astrocytes
BBB: Blood-brain barrier
CNS: Central nervous system
CSF: Cerebrospinal fluid
DEG: Differentially expressed genes
DLPFC: Dorsolateral prefrontal cortex
EC: Entorhinal cortex
ECM: Extracellular matrix
FACS: Fluorescence-activated cell sorting
FANS: Fluorescence-activated nuclear sorting
ICC: Immunocytochemistry
IF: Immunofluorescence
IHC: Immunohistochemistry
IPSC: Induced pluripotent stem cells
GO: Gene ontology
GVU: Gliovascular unit
HC: Hippocampus
MTX: Midtemporal cortex
Per: Pericytes
PFC: Prefrontal cortex
QC: Quality control
RNAseq: RNA sequencing
scRNAseq: Single cell RNA sequencing
SFX: Superior frontal cortex
SMC: Smooth muscle cells
snRNAseq: Single nucleus RNA sequencing
TH: Thalamus
TCX: Temporal cortex
UMAP: Uniform Manifold Approximation and Projection
UMI: Unique molecular identifier

Supplementary Results

Postmortem Results:

Ligand-target interactions between astrocytic and vascular AD-associated genes:

For completeness, expression levels of the six prioritized vascular target genes were also tested for associations with AD-related neuropathologies (**Supplementary Figure 12B**), age, sex and *APOE*ε4 (**Supplementary Figure 12C**). The results are detailed below. In summary, neuropathology associations are consistent with that expected from AD-related DEG findings, and some associations were also detected with sex and age, but not with *APOE*ε4.

We explored the expression profile of the 6 prioritized vascular genes for association with key AD variables including Braak stage, Thal phase, age, sex, and *APOE*ε4. We hypothesized that elevated expression of these genes is associated with increased AD pathology. For *ANGPT2*, expression in endothelial cl.26 is used, whereas for other genes expression in pericyte cl.25 is used, per their NicheNet results. As expected, all 6 vascular target genes that are higher in AD brains also have higher expression with increasing Braak stages and Thal phase (**Supplementary Figure 12B**). These associations are statistically significant ($q<0.05$) except for the Thal phase association with *AHNAK* levels ($q=0.13$). All genes are also higher in the presence of TDP-43 pathology with *ANGPT2* and *TSC22D3* reaching significance ($q<0.05$).

There are also significant associations with age and sex, but not *APOEε4*. Brain levels of *ANGPT2*, *ECE1*, *STAT3* and *SMAD3* are reduced with aging (**Supplementary Figure 12C**). These associations are driven by the AD group for *ECE1* and *SMAD3*, which have significant diagnosis by age interaction for expression associations, but not the other genes. Regarding sex differences, *ANGPT2* and *STAT3* expression is significantly lower in males in the combined group, which seems to be driven by controls, especially for the latter. *SMAD3* is lower in AD males. Collectively, these results demonstrate that while

all 6 vascular targets are higher in AD temporal cortex and associated with increasing AD-related pathologies, the levels of some of these genes are also influenced by age and sex in a differential manner by diagnosis.

Association of blood *SMAD3* gene expression levels with infarcts, Aβ deposition and cortical atrophy:

Since *APOE* or sex can influence brain vascular burden[1,2], we also performed secondary analyses of *SMAD3* locus variants with infarcts and blood *SMAD3* levels, in *APOE*-ε4 or sex-stratified cohorts. The direction of effect remained the same in all strata, though as expected significance was lower given smaller sample sizes (**Supplementary Data 35**)**.** Effect sizes for both infarct and *SMAD3* blood expression associations were similar between *APOE*-ε4 carriers and non-carriers but greater for males compared to females. These findings suggest that higher blood *SMAD3* levels may have a protective effect against infarcts in a non-*APOE* dependent manner, though this effect may be stronger for males.

Supplementary Figures

Supplementary Figure 1 Neuropathological measures in AD and control samples
Supplementary Figure 2 Purity and quality of isolated and purified nuclei
Supplementary Figure 3 Effect of fluorescence-activated nuclei sorting (FANS) to cell type distribution
Supplementary Figure 4 Parametric results of snRNAseq data after quality control and filtration steps
Supplementary Figure 5 snRNAseq nuclei clusters on UMAP by features
Supplementary Figure 6 snRNAseq cluster nuclei proportion distributions
Supplementary Figure 7 Selected signature genes in brain vascular nuclei clusters
Supplementary Figure 8 Comparison of our study with previous study
Supplementary Figure 9 Heatmap of 40 astrocyte ligand genes identified in NicheNet analysis
Supplementary Figure 10 Astrocyte ligands and vascular targets
Supplementary Figure 11 Heatmap of 26 vascular target genes identified in NicheNet analysis
Supplementary Figure 12 Validation of six vascular prioritized genes
Supplementary Figure 13 *SMAD3* is a predicted target for multiple astrocytic ligands in AD
Supplementary Figure 14 High expression of *VEGFA* is detected in astrocytes
Supplementary Figure 15 Marker gene expression profile of pericytes in integrated datasets
Supplementary Figure 16 Marker gene expression profile of astrocytes in integrated datasets
Supplementary Figure 17 Correlation amongst *SMAD3* probes
Supplementary Figure 18 QC of utilized IPSCs
Supplementary Figure 19 Validation of *SMAD3* gene expression through RNAscope
Supplementary Figure 20 Experimental design for the treatment of IPSC derived pericytes
Supplementary Figure 21 Diagnosis stratified response of IPSC derived pericytes
Supplementary Figure 22 Pericyte *SMAD3* levels at different VEGF treatment concentration
Supplementary Figure 23 Validation of human snRNAseq results on transgenic zebrafish
Supplementary Figure 24 FACS Gating strategy of zebrafish brain cells
Supplementary Figure 25 QC Results of Aβ and PBS treated transgenic zebrafish results
Supplementary Figure 26 Structural comparison of VEGFR2 in human and zebrafish
Supplementary Figure 27 Zebrafish Immunostaining quantification strategies
Supplementary Figure 28 FANS Gating strategy of intact nuclei from frozen human brain
Supplementary Figure 29 Fold change in gene expression profile of iPSC-derived pericytes
Supplementary Figure 30 Graphical abstract

Supplementary Figure 1:

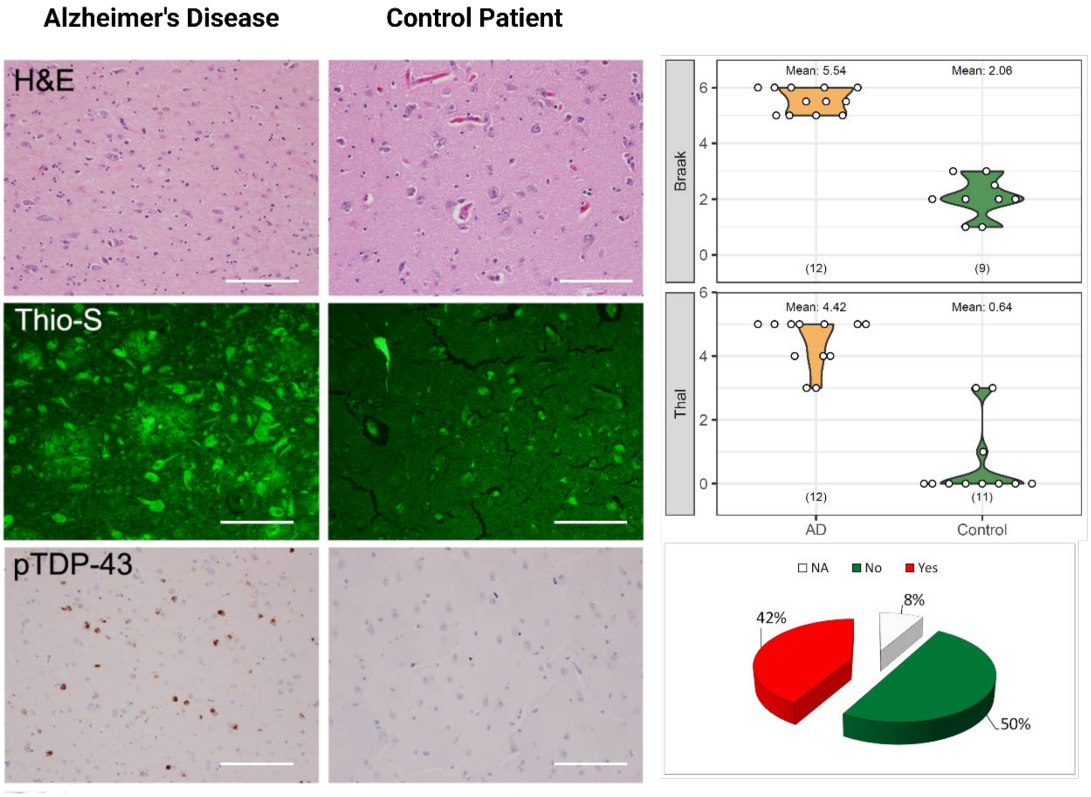

Supplementary Figure 1: Neuropathological measures in AD and control samples. Left panels depict representative immunohistochemistry pictures from an Alzheimer's patient and control brain amygdala tissue slides for H&E, thioflavin-S and TDP-43 staining. The white scale bar in each panel equals 200 µm. Right sided panels from top to bottom show distribution of Thal phase, Braak stage and presence or absence of TDP-43 in the cohort of 12 AD and 12 control brains. Source data are provided as a Source Data file.

Supplementary Figure 2:

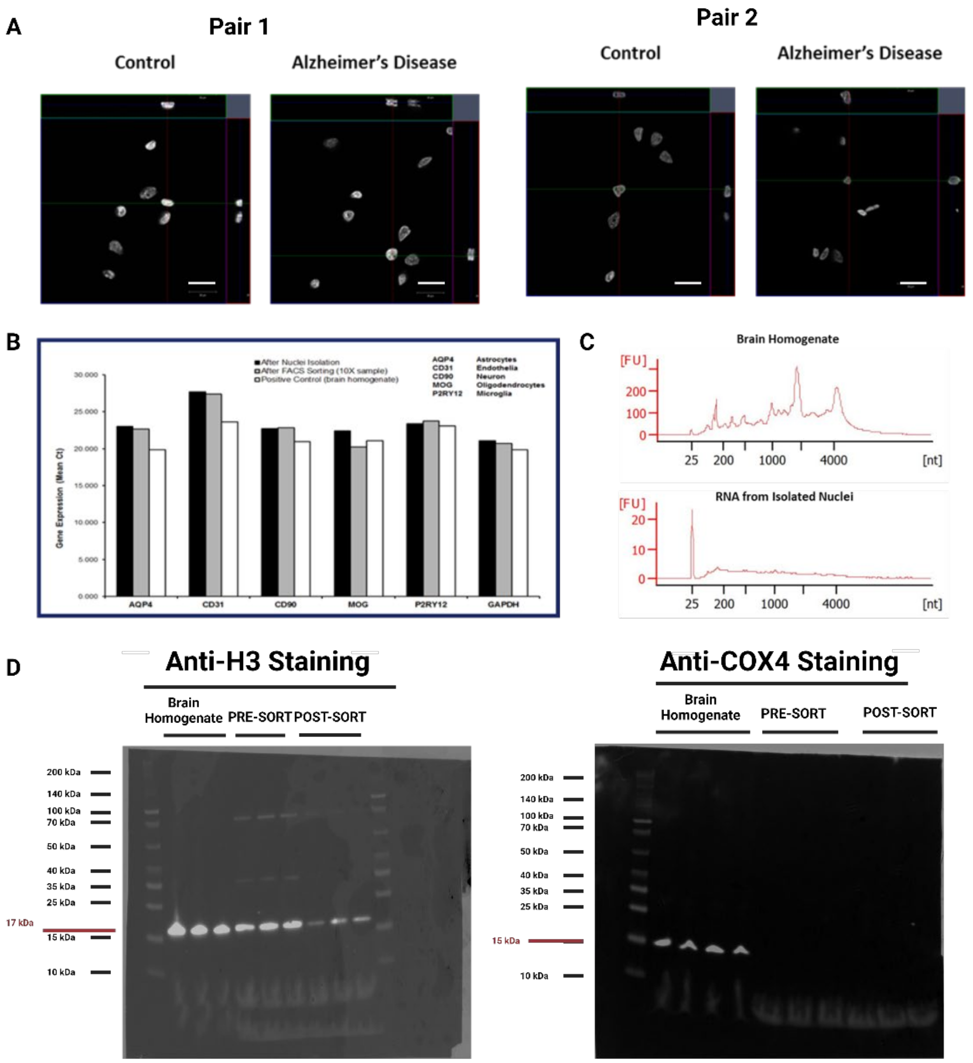

Supplementary Figure 2: Purity and quality of isolated and purified nuclei. (A): Intact nuclei were observed under confocal microscope. The scale bar equals 20 µm. (B) qPCR was performed with cell type specific TaqMan probes from isolated nuclear RNA demonstrate lack of bias in detecting various cell types. (C) Disappearance of 18S and 28S peaks in Bioanalyzer histogram demonstrates the disappearance of cytoplasmic RNA from the nuclear fraction. (D): Effect of FANS to purity of isolated nuclei was measured using Nuclear (Anti-H3) and mitochondrial (Anti-COX4) antibodies. This experiment was completed once prior to snRNAseq data generation.

Supplementary Figure 3:

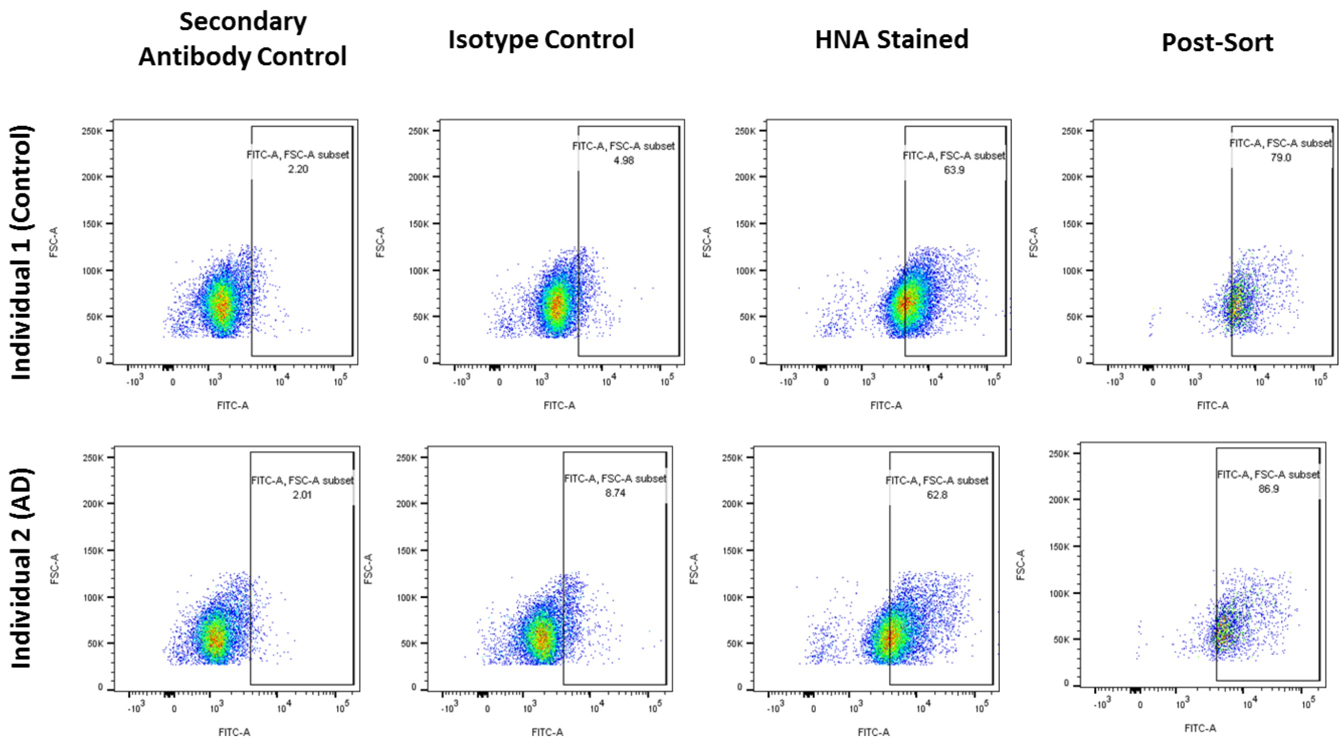

Supplementary Figure 3: Effect of fluorescence-activated nuclei sorting (FANS) to cell type distribution. Upper: FANS profile of the sorted nuclei. Lower: effect of FANS sorting to the distribution of cell types.

Supplementary Figure 4:

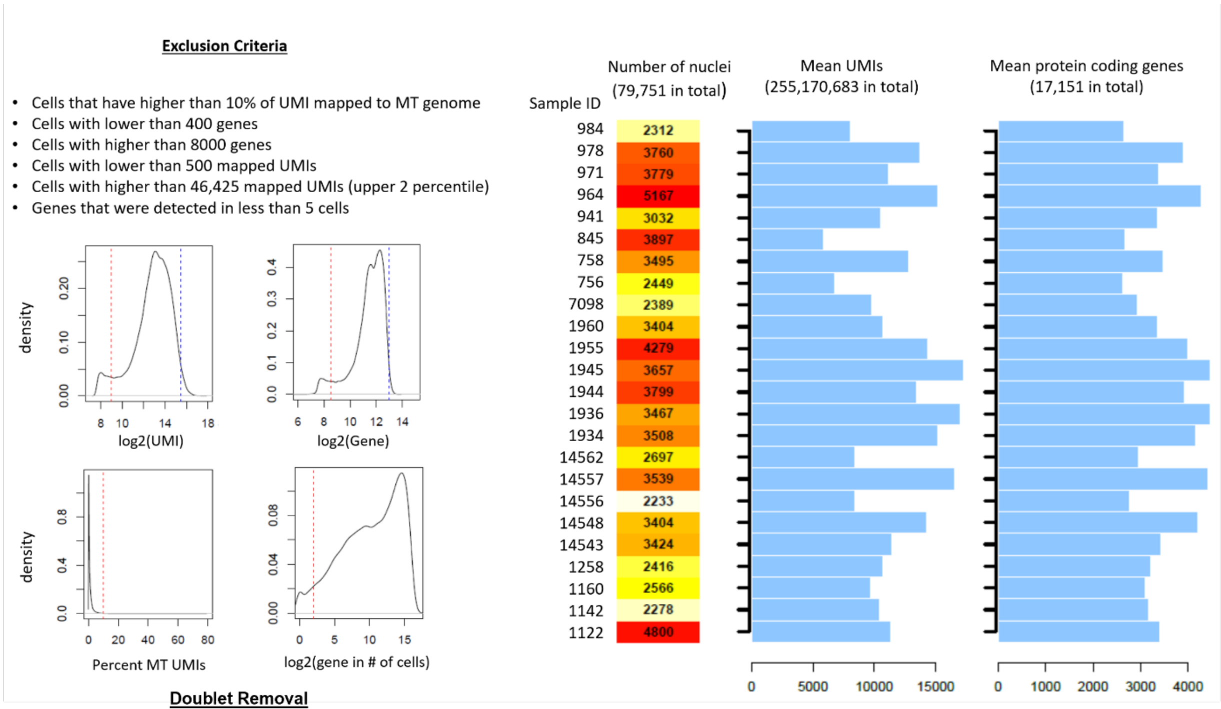

Doublet Removal

SampleID	1122	1142	1258	941	1160	756	845	14543	14557	14562	1934	1945	14548	14556	1944	1936	1955	1960	7098	758	964	971	978	984
Singlet	4644	2265	2369	3021	2513	2373	3830	3314	3487	2684	3434	3568	3392	2190	3747	3453	4176	3386	2350	3475	4988	3695	3743	2299
Doublet	156	13	47	11	53	76	67	110	52	13	74	89	12	43	52	14	103	18	39	20	179	84	17	13

Stacked bar plot of percent of nuclei from each donor in each cluster

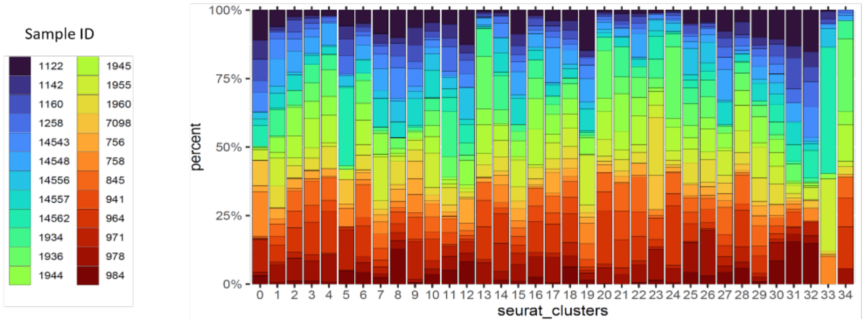

Supplementary Figure 4: Parametric results of snRNAseq data after quality control and filtration steps. Top left: Exclusion criteria for nuclei and genes. Bottom left: Density plots: UMI=unique molecular identifier. Top right: Post-QC number of nuclei, mean UMI and number of protein coding genes per each sample. Table: the number of doublets and singlets detected by Srublet. These doublets were removed from downstream analysis. Lower: To test if there are over-representations of nuclei from donor(s) in each cluster, we followed the approach of Wang et al. [3] to perform one-sided Fisher's Exact Test (FET).

Supplementary Figure 5:

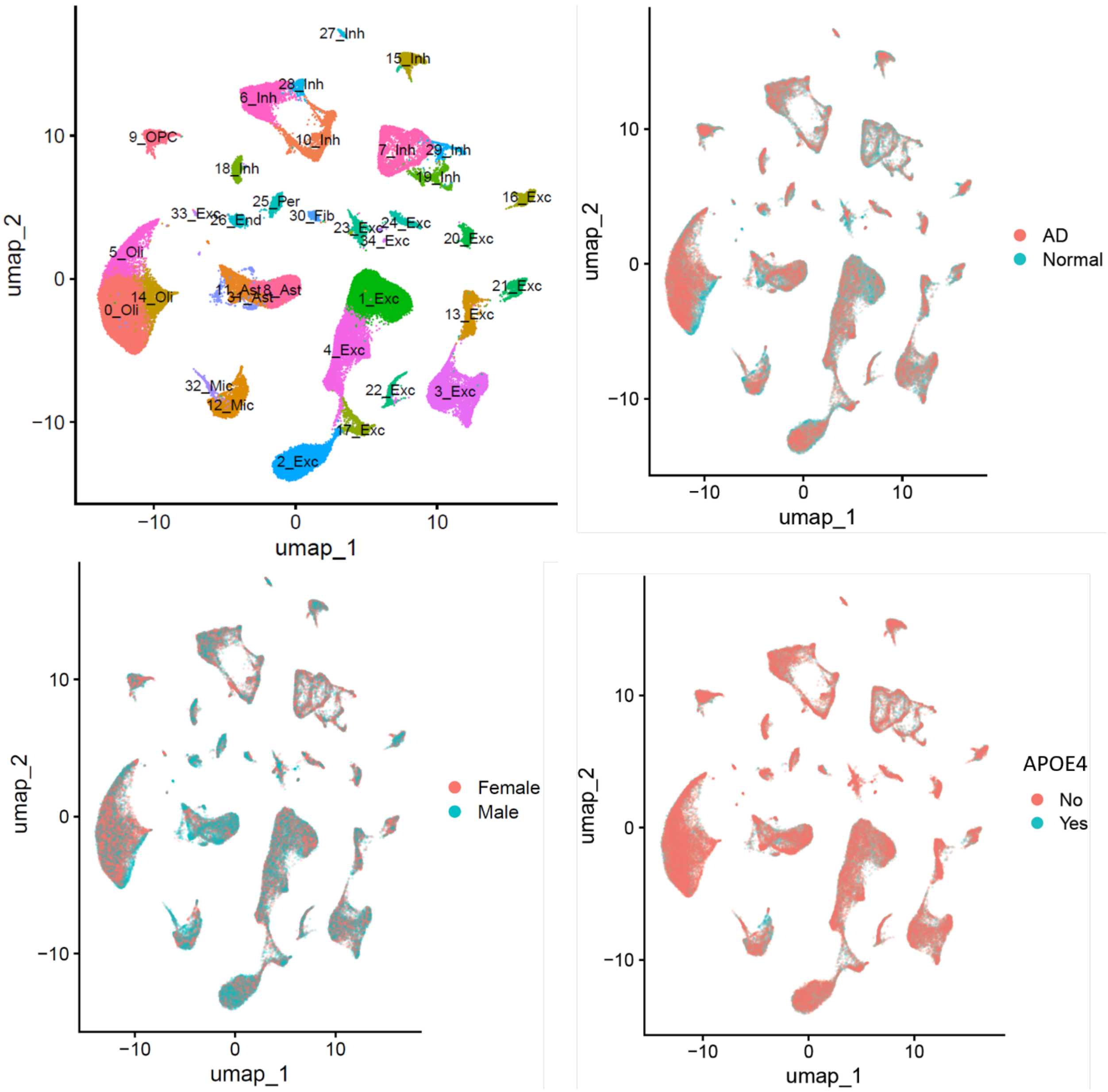

Supplementary Figure 5: snRNAseq nuclei clusters on UMAP by features: Top left: Nuclei clusters displayed on UMAP reduced dimension space, annotated as cluster number followed by cell type. Exc: excitatory neuron; Inh: inhibitory neuron; Oli: oligodendrocyte; OPC: oligodendrocyte progenitor cell; End: endothelia; Per: pericyte; Fib: fibroblast; Ast: astrocyte; Mic: microglia. Top right and bottom: Distributions are colored by variables. “APOE4 Yes” means either homozygotes or heterozygotes.

Supplementary Figure 6:

A. Cluster nuclei proportions by diagnosis

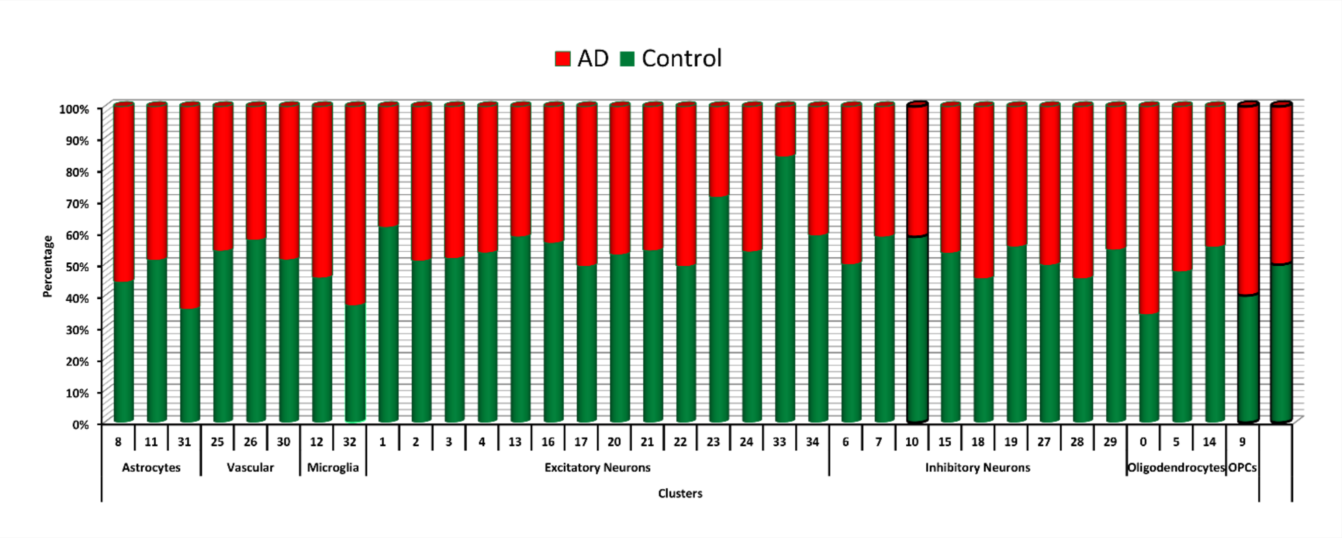

B. Cluster nuclei proportions by Braak stage

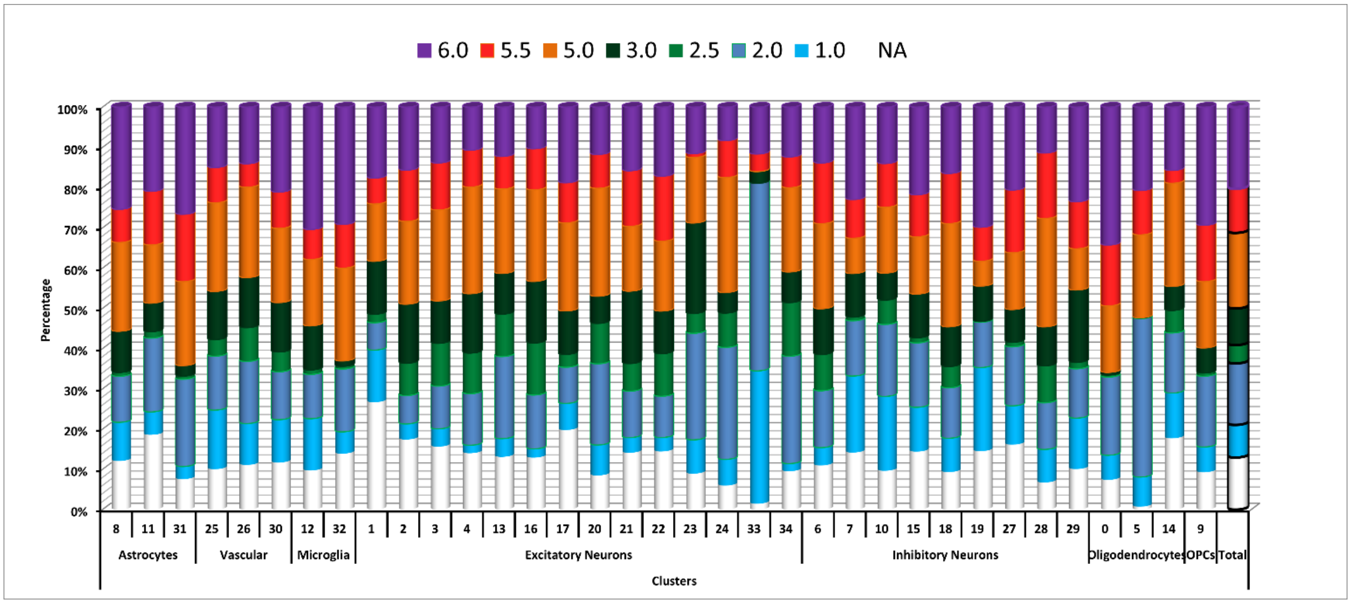

C. Cluster nuclei proportions by Thal phase

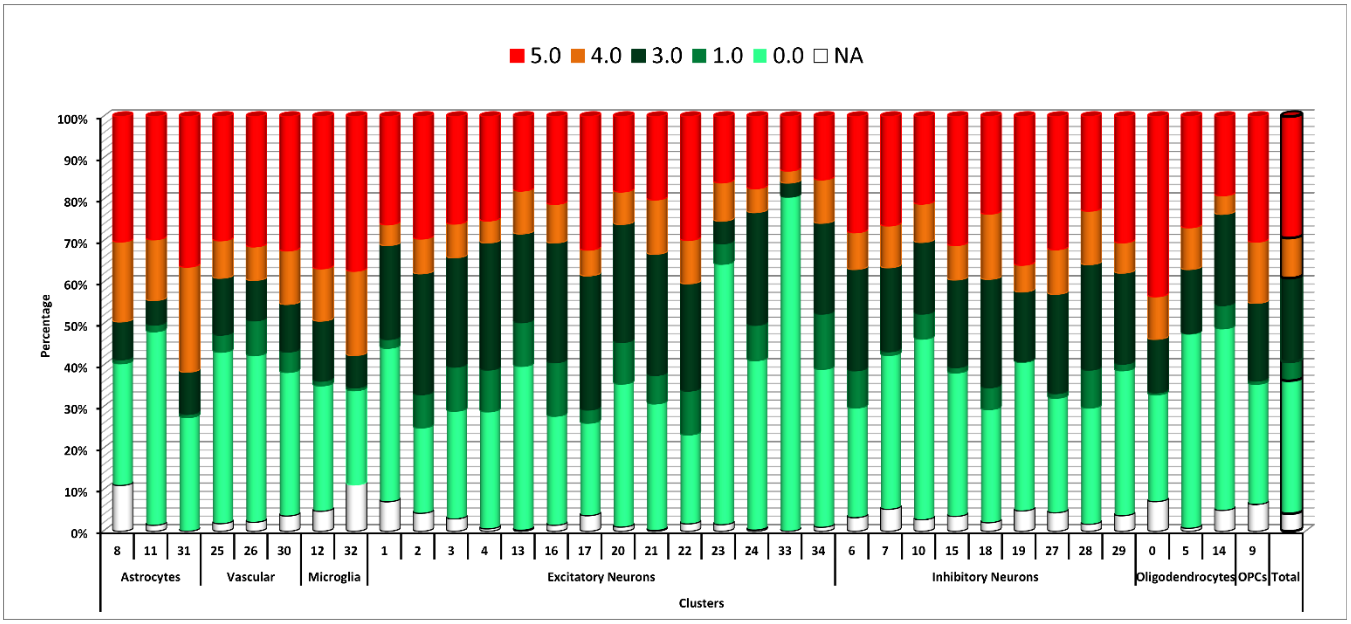

D. Cluster nuclei proportions by TDP43

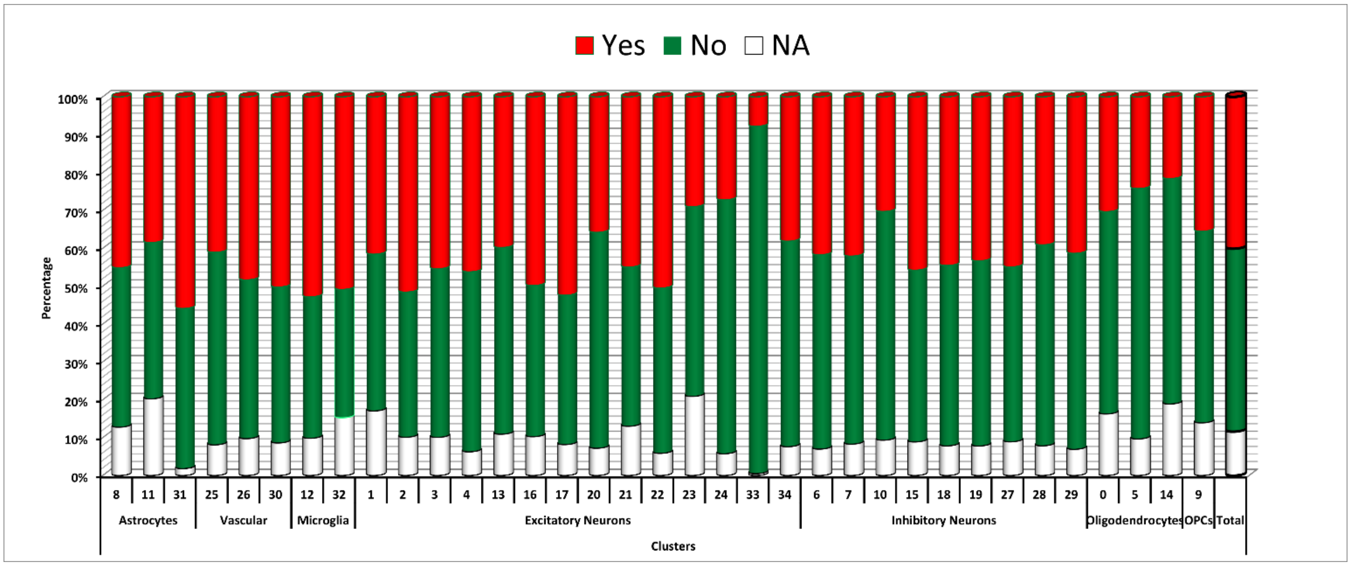

E. Cluster nuclei proportions by sex

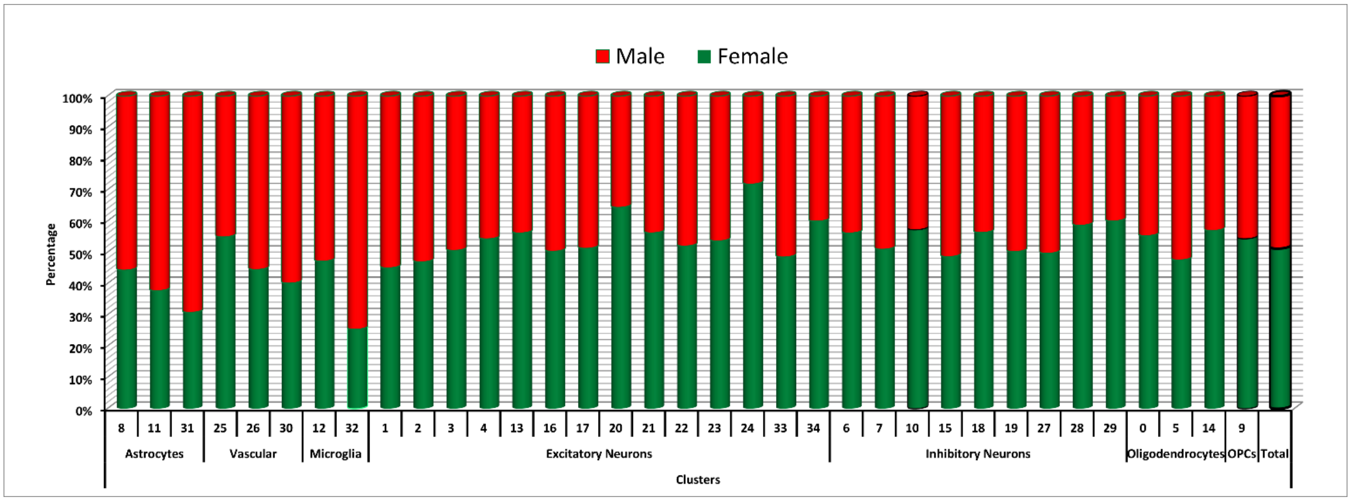

F. Cluster nuclei proportions by *APOE*

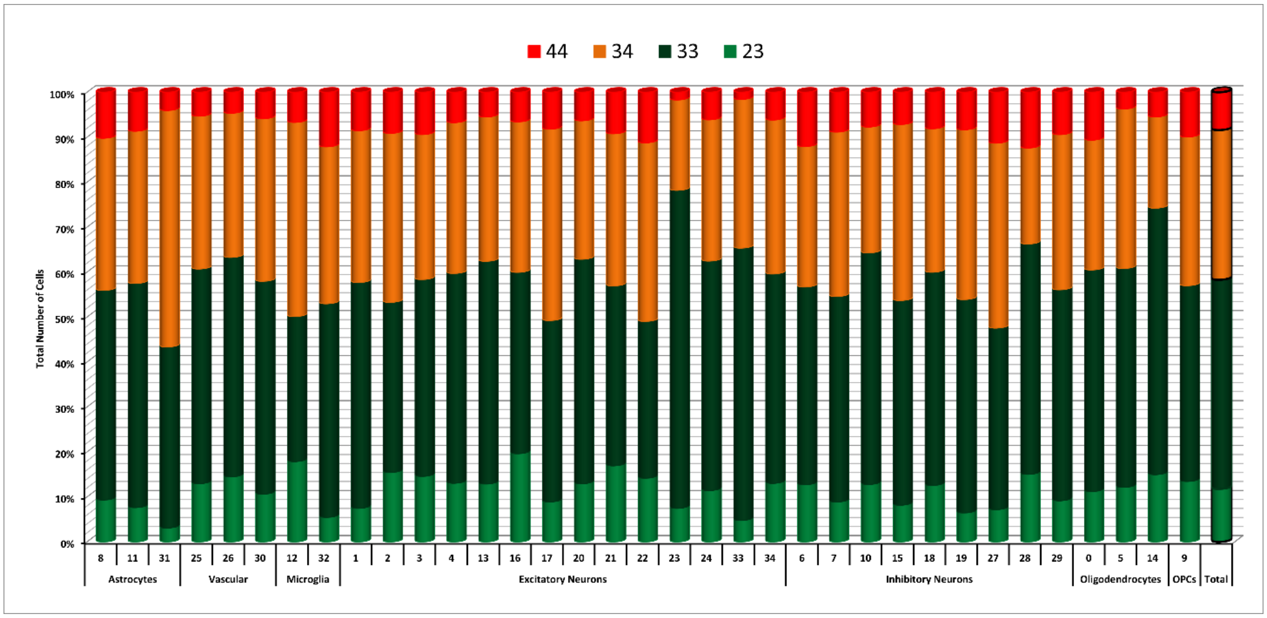

G. Cluster nuclei proportions by age

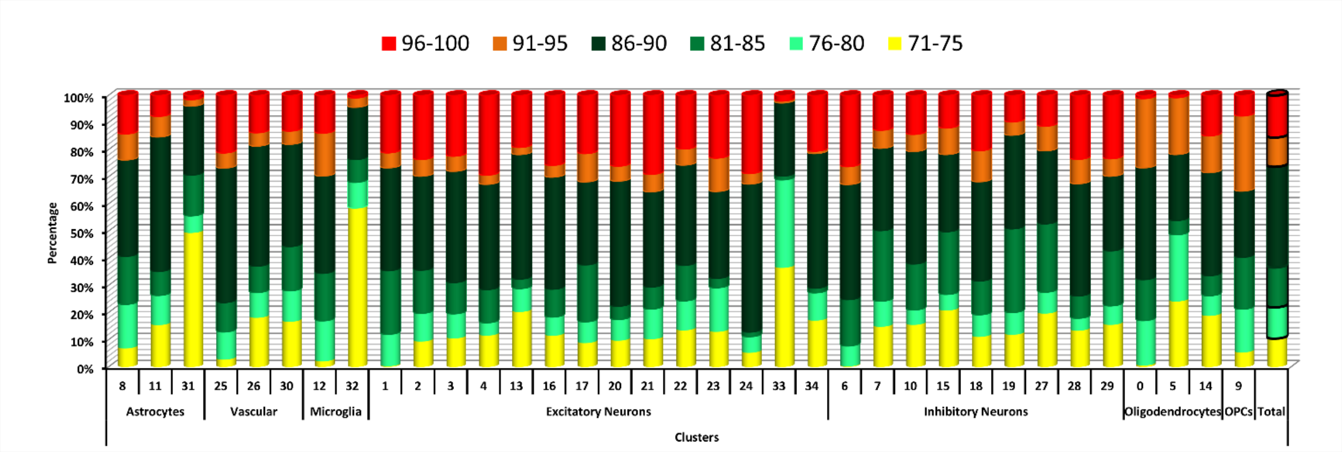

Supplementary Figure 6: snRNAseq cluster nuclei proportion distributions by A) diagnosis, B) Braak Stage, C) Thal phase, D) TDP-43, E) sex, F) *APOE* and G) age. Source data are provided as a Source Data file.

Supplementary Figure 7:

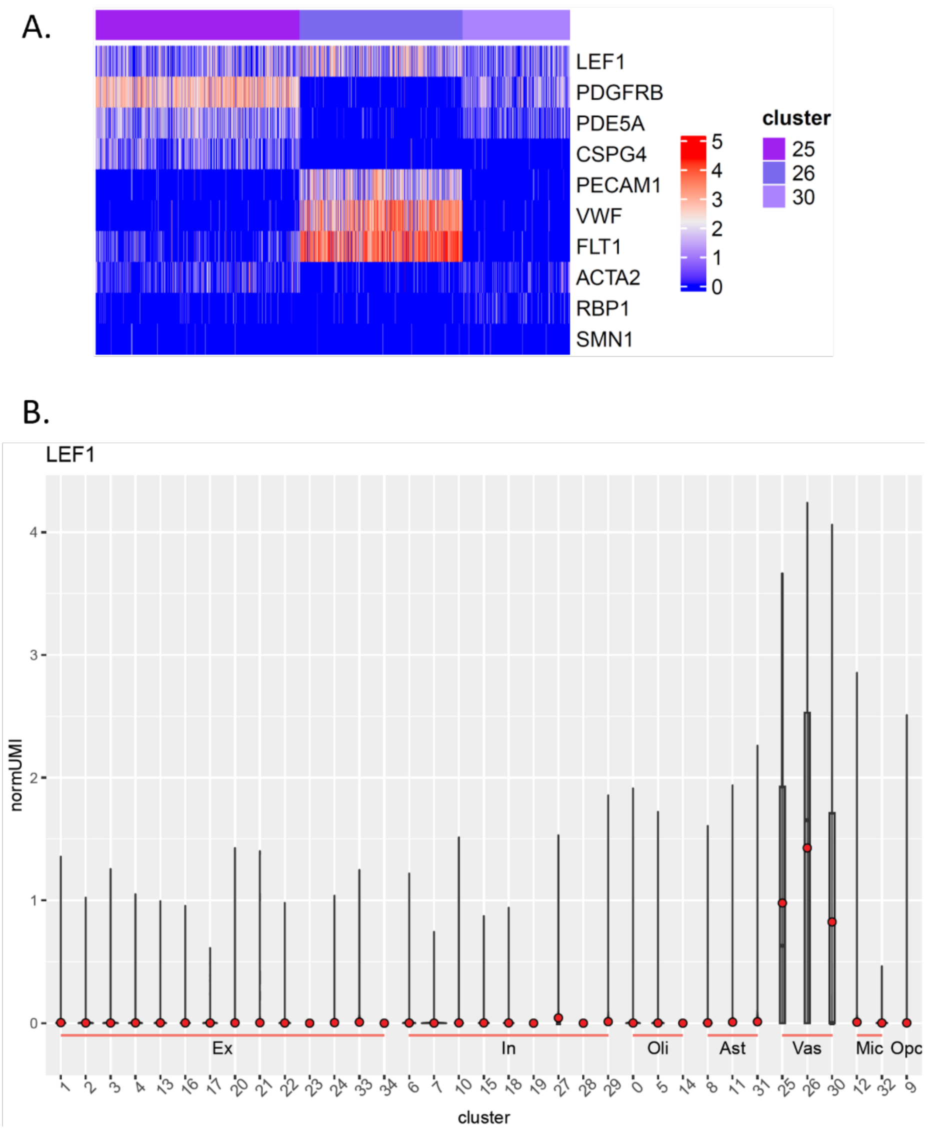

Supplementary Figure 7: Selected signature genes in brain vascular nuclei clusters. A. Expression of selected signature genes in vascular clusters. Cluster 25 (cl.25)=pericyte, cl.26=endothelia, cl.30=perivascular fibroblast. The scale bar indicates the mean log-normalized UMI counts. **B.** Only vascular nuclei clusters demonstrated expression of *LEF1.*

Supplementary Figure 8:

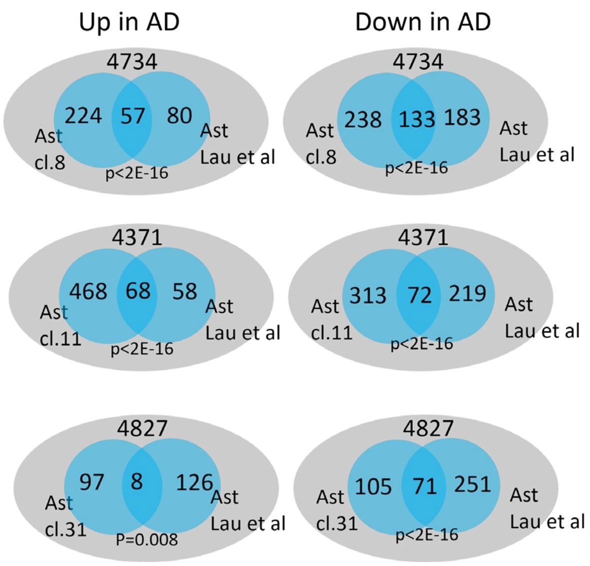

Supplementary Figure 8: Comparison of our study with previous study. Number of overlapping DEGs between astrocytic clusters in our study (cl.8, cl.112 and cl.31) and those from Lau et al. study[4]. The gray circles and the numbers indicate the detected genes in both Lau et al astrocyte cluster and our astrocytic clusters cl.8, cl.11 and cl.31 respectively. Enrichment p-value is from Fisher's exact test. Source data are provided as a Source Data file.

Supplementary Figure 9:

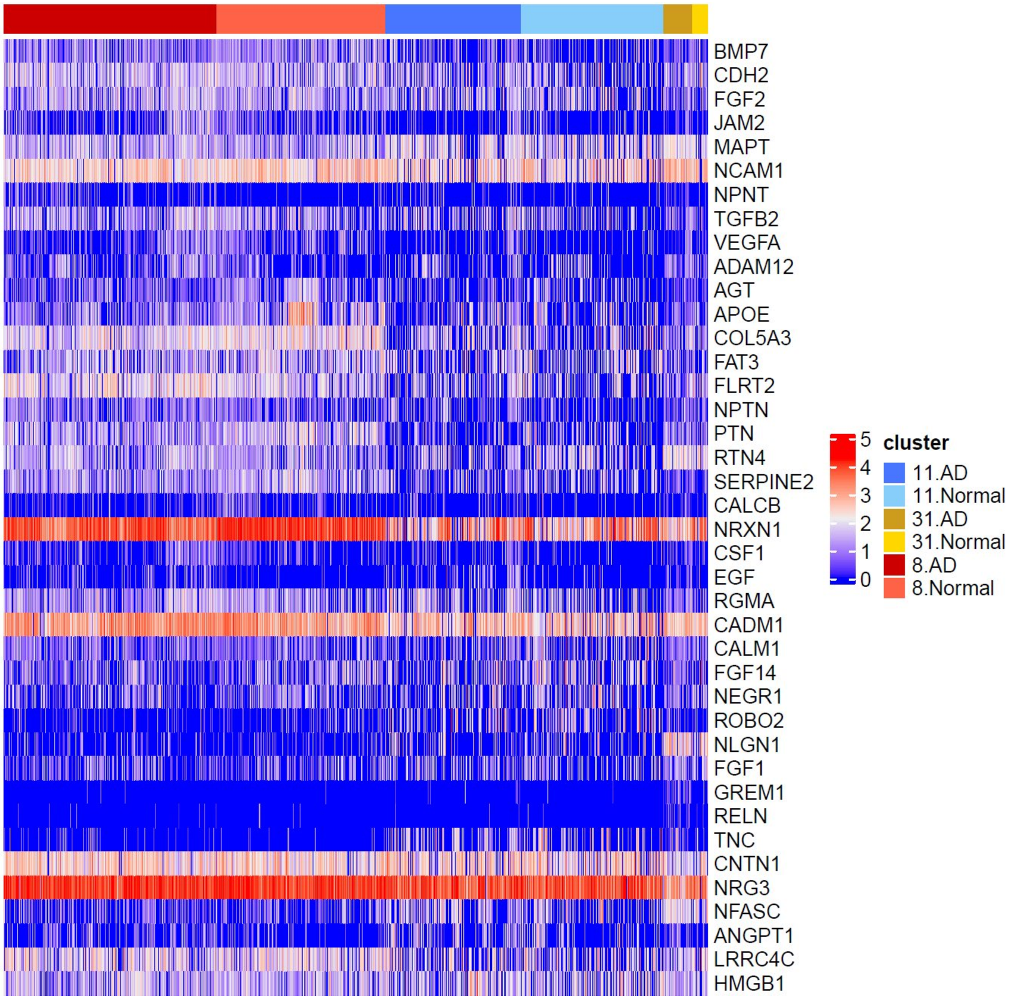

Supplementary Figure 9: Heatmap of 40 astrocyte ligand genes identified through ligand-target interaction analysis platform Nichenet[5]. This analysis used significant DEGs between AD and control tissue identified in astrocyte clusters (cl.8, cl.11 and cl.31) as ligands and those in endothelial clusters (cl.25, cl.26 and cl.30) as potential targets. For more detailed description of this analysis, please see main text and method. Columns are cells arranged by clusters and diagnosis. Values shown in the heatmap are normalized expression.

Supplementary Figure 10:

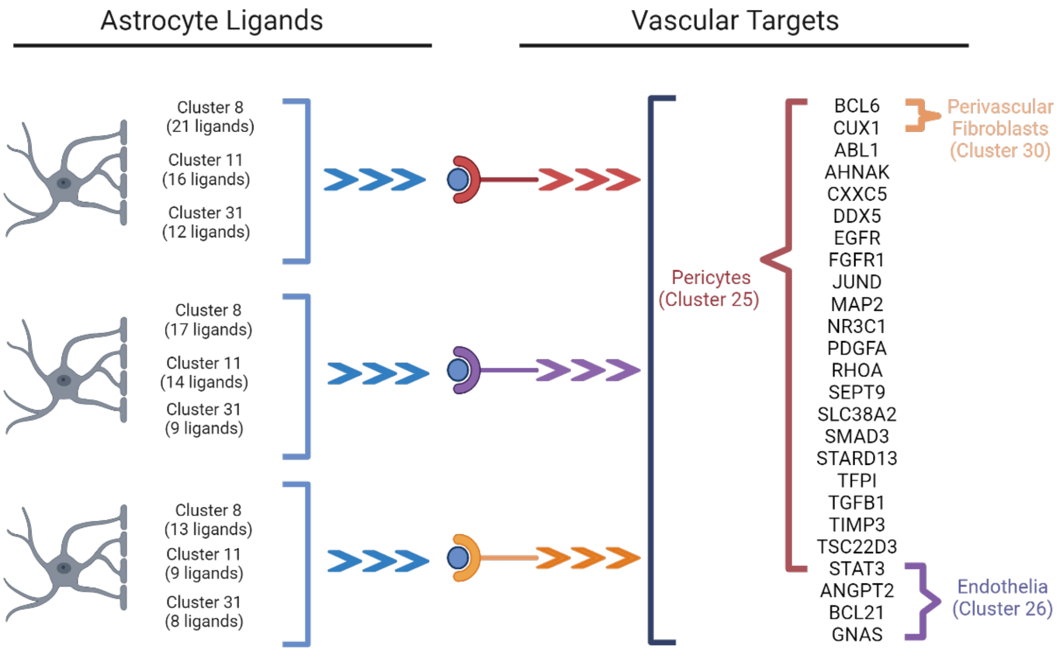

Supplementary Figure 10: Astrocyte ligands and vascular targets. Using Nichenet[5] and focusing on significant DEGs in astrocytic clusters, we identified a combined pool of 40 unique potential ligand genes that have corresponding targets in one or more of the vascular clusters. There were 22, 4 and 2 predicted vascular targets in the pericyte cl.25 (red), endothelial cluster cl.26 (purple) and perivascular fibroblasts cluster cl.30 (orange), respectively, comprising 26 unique target genes (Supplementary Figure 10 Created with BioRender.com released under a Creative Commons Attribution-NonCommercial-NoDerivs license).

Supplementary Figure 11:

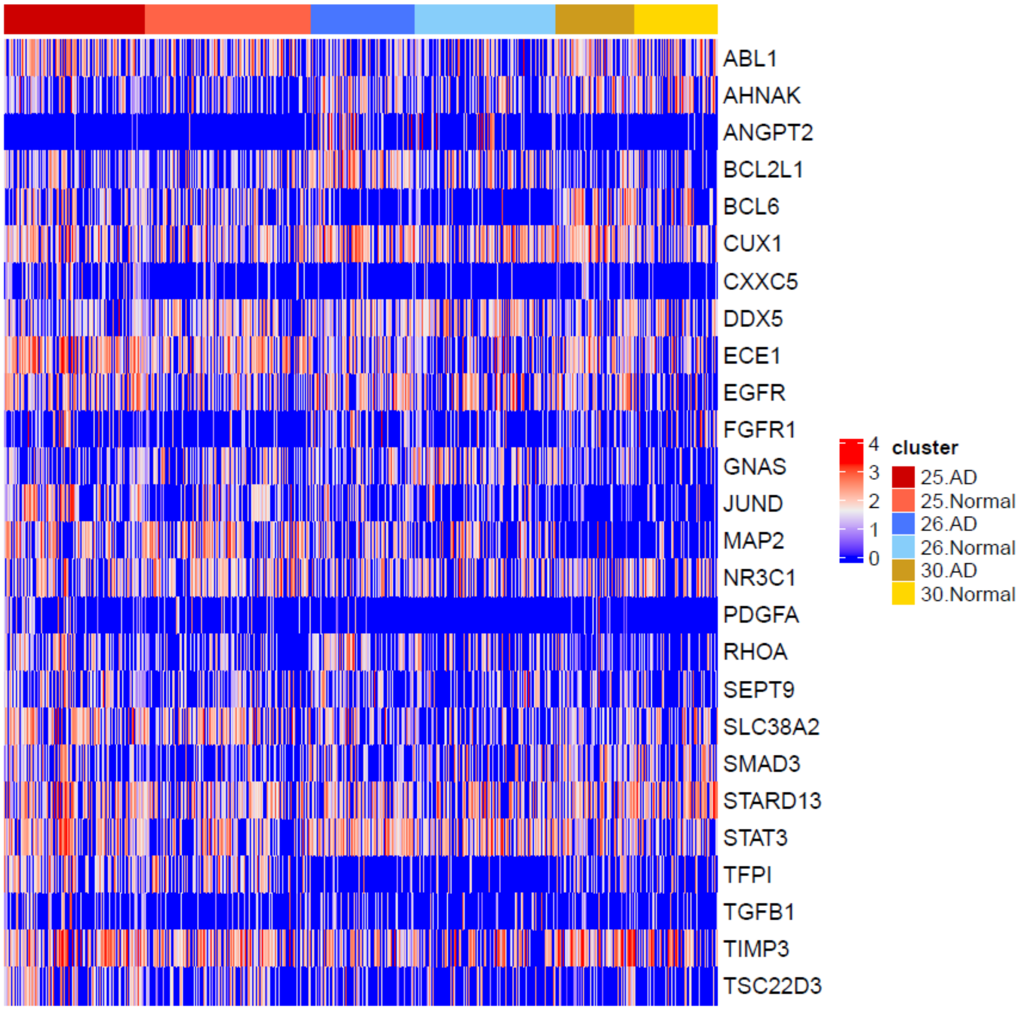

Supplementary Figure 11: Heatmap of 26 vascular target genes identified through ligand-target interaction analysis platform Nichenet[5]. This analysis used DEGs between AD and control tissue identified in astrocyte clusters (cl.8, cl.11 and cl.31) as ligands and those in endothelial clusters (cl.25, cl.26 and cl.30) as potential targets. For more detailed description of this analysis, please see main text and method. Columns are cells arranged by clusters and diagnosis. Values shown in the heatmap are normalized expression.

Supplementary Figure 12:

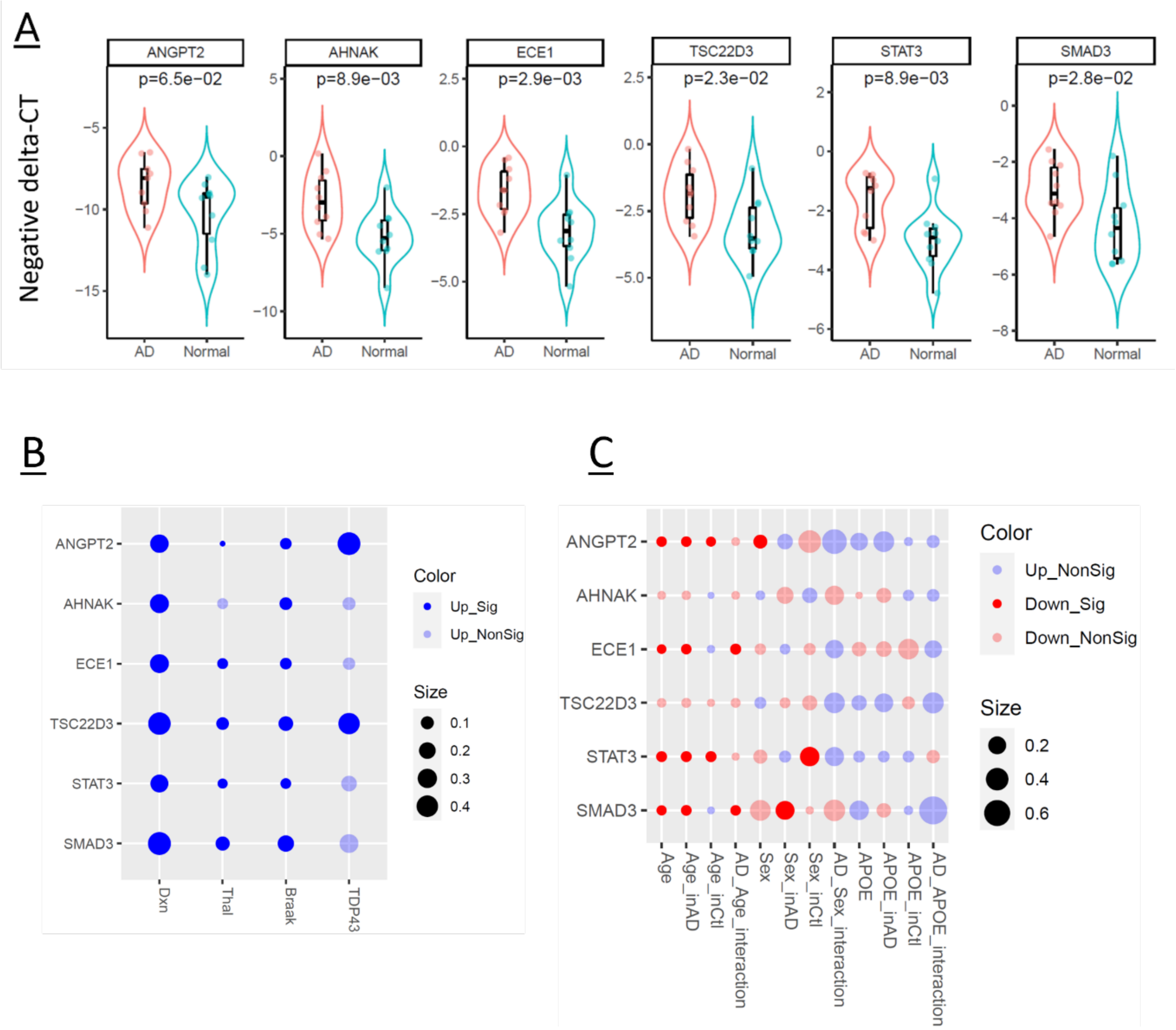

Supplementary Figure 12: Validation of six vascular prioritized genes. Expression of six prioritized vascular target genes were validated via qPCR from nuclear fractions. A) Distribution of negative delta-CT from qPCR experiment of six prioritized vascular target genes, which reflect the expression of these gene. Two-sided Wilcoxon rank sum tests were performed to test whether these genes were expressed higher in AD compared to control nuclei, as observed from snRNAseq. B) The association between AD-related pathologies and snRNAseq expression of six vascular genes. Blue bubble: significant positive association with pathologies at q-value≤0.05. Red bubble: significant negative association with pathologies at q-value≤0.05. Gray-masked bubble: not significant. Size of the bubble reflects the association coefficient. C) The association between age at death, sex and *APOEε4* and expression of six prioritized vascular genes in combined AD and controls, ADs only and controls only, respectively, as shown in the first three columns for each association. The fourth column for each association shows the interaction effects between age, sex or *APOEε4* with diagnosis on gene expression. A significant interaction effect indicates that the association between the risk factor and gene expression is dependent on disease status. Blue bubble: significant positive association at q-value≤0.05. Red bubble:

significant negative association at q-value≤0.05. As males or *APOEε4* carriers were denoted as 1 whereas females or *APOEε4* non-carriers as 0, blue means higher in males or *APOEε4* carriers and red means lower in males or *APOEε4* carriers. Gray-masked bubble: not significant. Size of the bubble reflects the association coefficient. Source data are provided as a Source Data file.

Supplementary Figure 13:

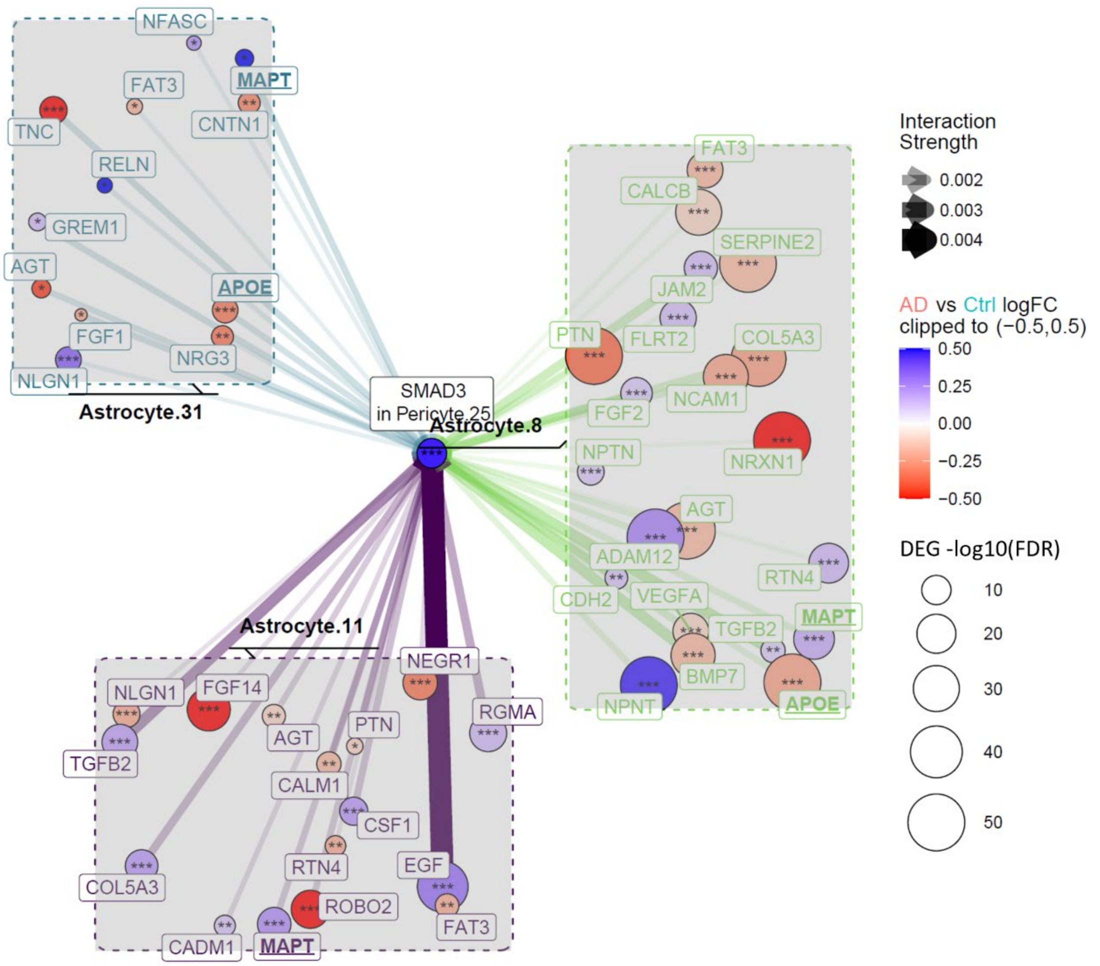

Supplementary Figure 13: *SMAD3* is a predicted target for multiple astrocytic ligands in AD from NicheNet. DEG (differentially expressed gene) is from comparing AD and control nuclei using MAST. The figure outlines the connections between astrocytic ligand genes to SMAD3 based on the interaction strengths in three astrocytic clusters. SMAD3 ligands include well-known AD genes in astrocytes such as APOE, MAPT, PSEN1, and APP. Line thickness: the Astrocytic gene's connection strength to SMAD3; Red Blue gradient heatmap: Fold change of differential expressed gene expression in AD vs control (downregulated (blue) and upregulated (red)); Radius of circle: FDR corrected significance between AD vs controls. Source data are provided as a Source Data file.

Supplementary Figure 14

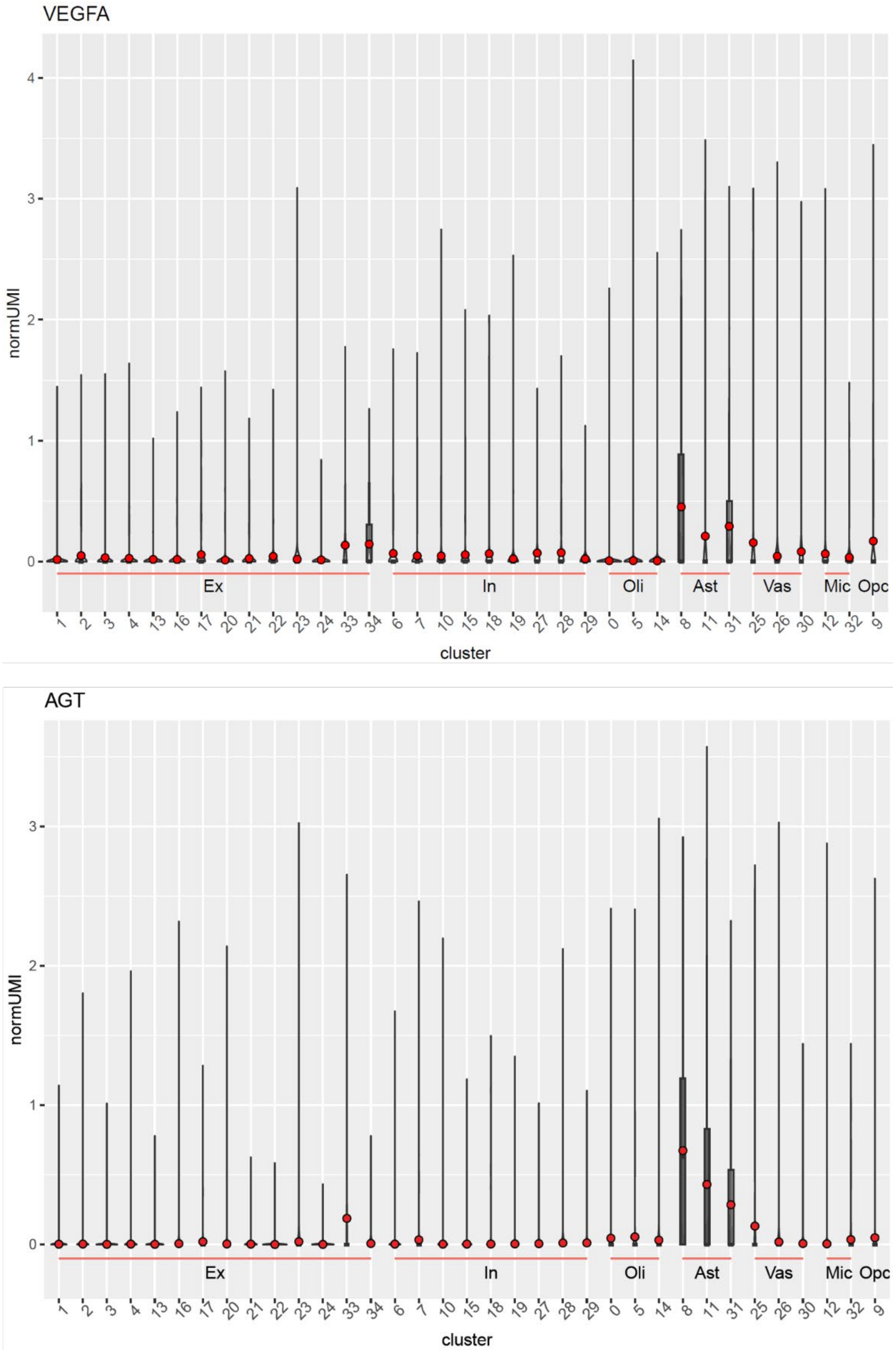

Supplementary Figure 14: **High expression of *VEGFA* is detected in astrocytes.** In human temporal cortex, *AGT* expression is restricted to astrocytes.

Supplementary Figure 15

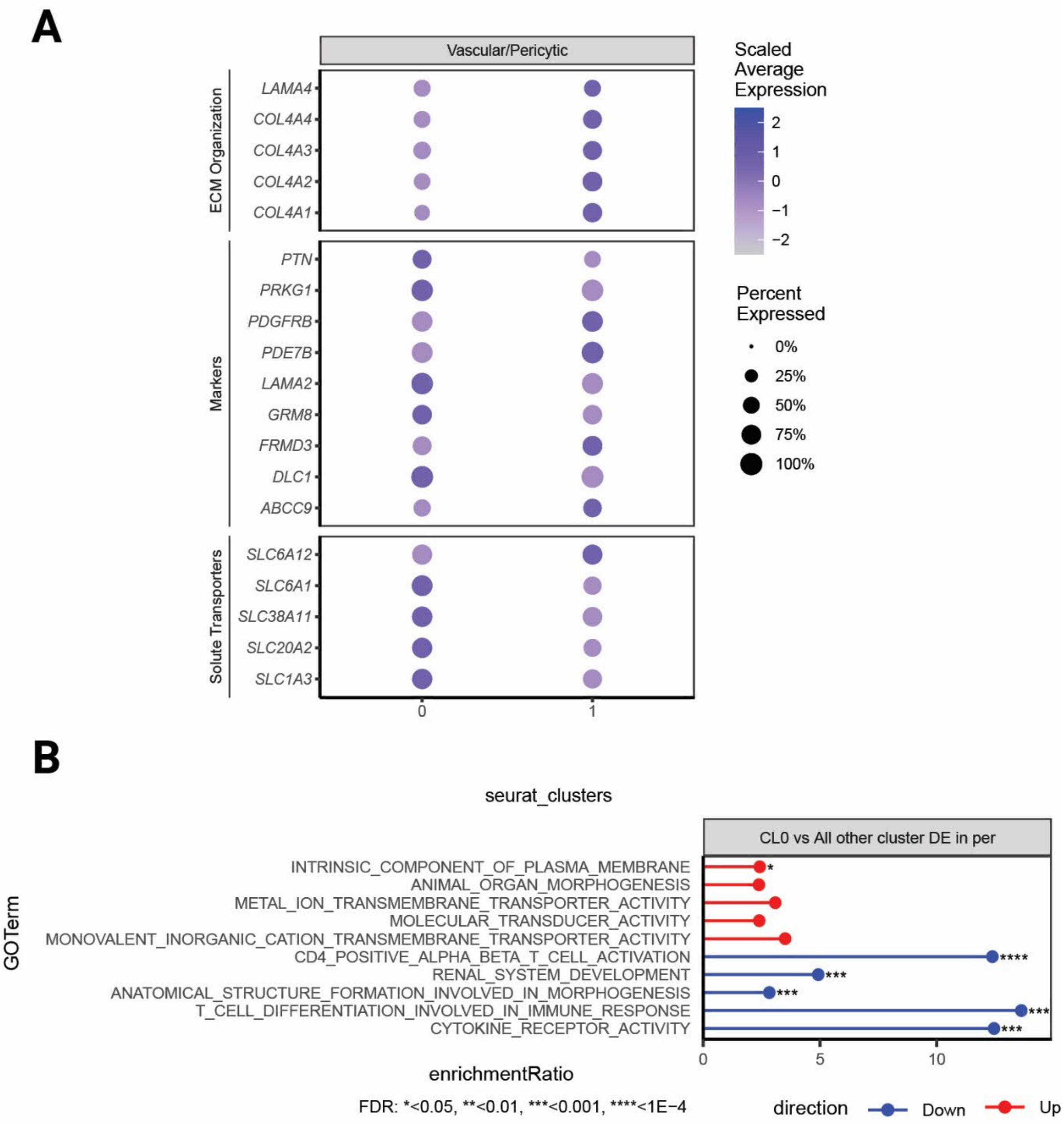

Supplementary Figure 15: Marker gene expression profile of pericytes in integrated datasets. A) Despite highly similar transcription profile, pericyte cluster 0 showed slight upregulation in the genes that regulate solute transporters. **B)** GO Term pathway analysis in pericyte cluster 0 displayed downregulation in the genes that regulate immune functions. Source data are provided as a Source Data file.

Supplementary Figure 16

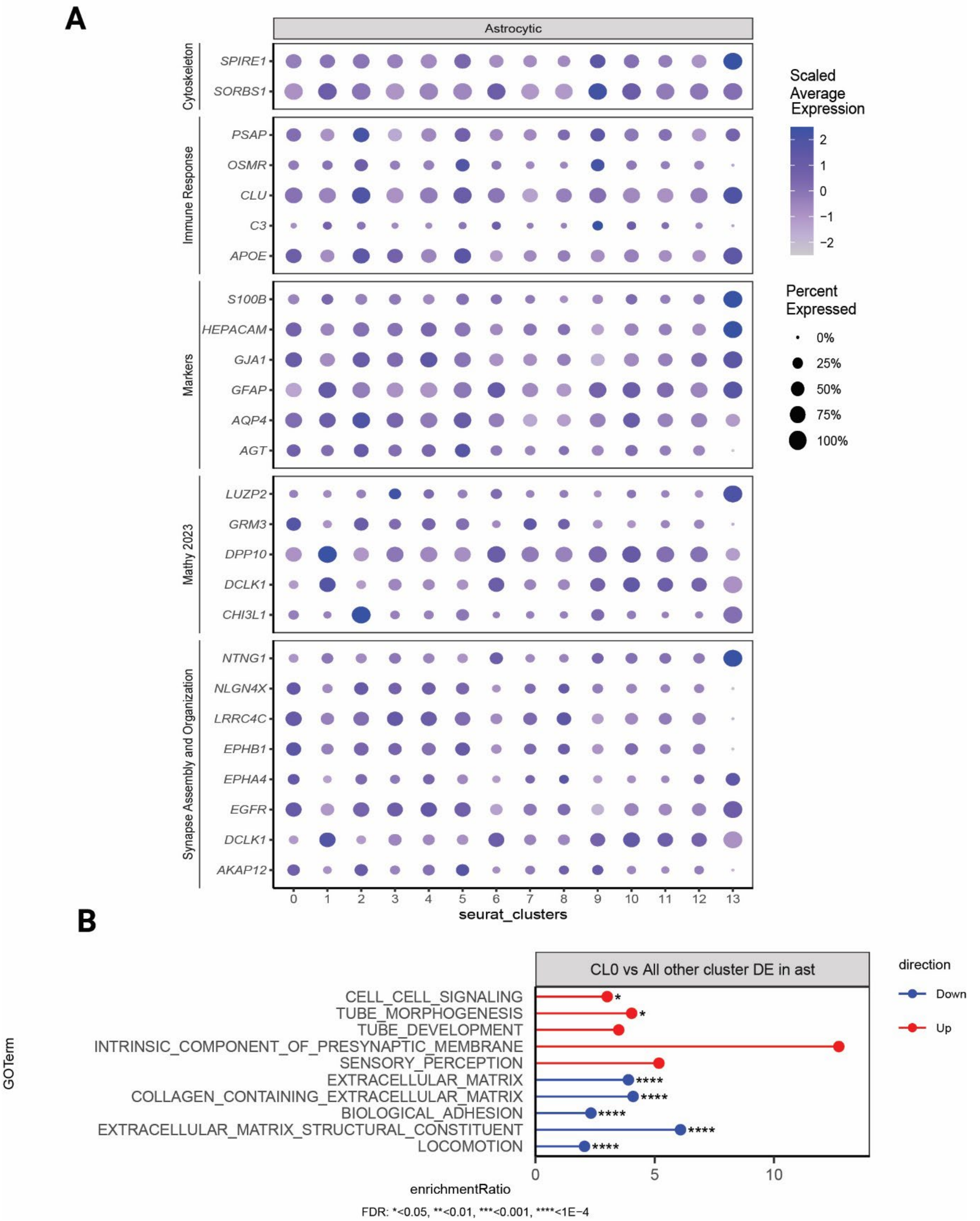

Supplementary Figure 16: Marker gene expression profile of astrocytes in integrated datasets. A) Despite highly similar transcription profile, astrocyte cluster 0 showed slight upregulation in the genes that regulate synapse assembly and organization. B) Top Enriched GO terms of signature genes in Ast.0 compared to all other astrocyte clusters are displayed. Upregulated genes are enriched in cell signalling

and tube morphogenesis and downregulated genes are enriched in the extracellular matrix related pathways. Source data are provided as a Source Data file.

Supplementary Figure 17

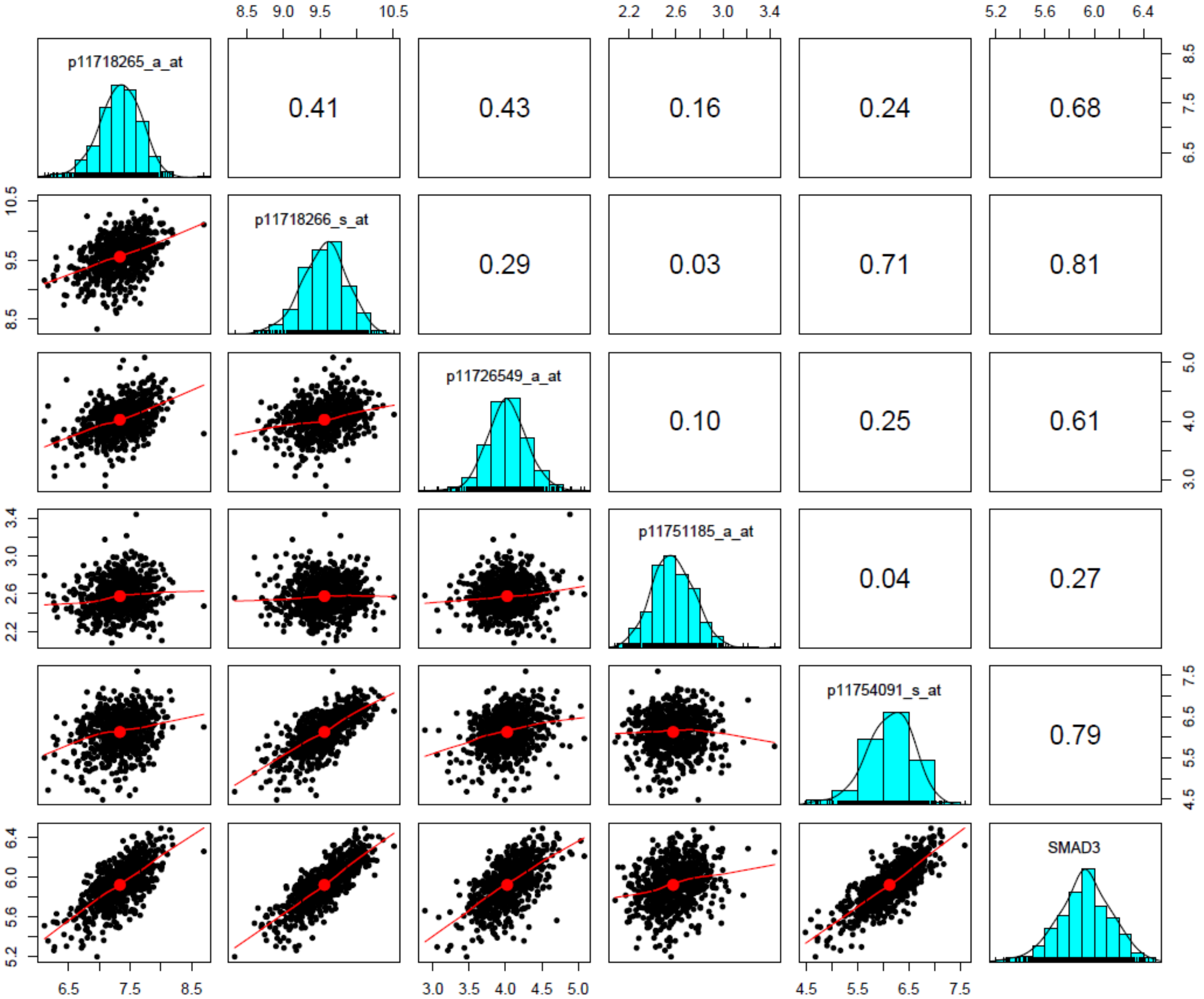

Supplementary Figure 17: **Correlation amongst *SMAD3* probes**. To evaluate the association of genetic variants with *SMAD3* gene expression, blood RNA expression quantified using Affymetrix Human Genome U219 Array (Affymetrix, Santa Clara, CA) for 5 *SMAD3* probes was obtained from 645 ADNI participants[6]. Histograms (teal) showing the distribution of expression quantified by each probe ('p11718265_a_at', 'p11718266_s_at', 'p11726549_a_at', 'p11751185_a_at', 'p11754091_s_at') as well the distribution of the average expression across all probes ('SMAD3') in shown across the diagonal in the figure. Pearson correlation coefficients are shown above the diagonal and scatter plots comparing expression values are shown below. Source data are provided as a Source Data file.

Supplementary Figure 18

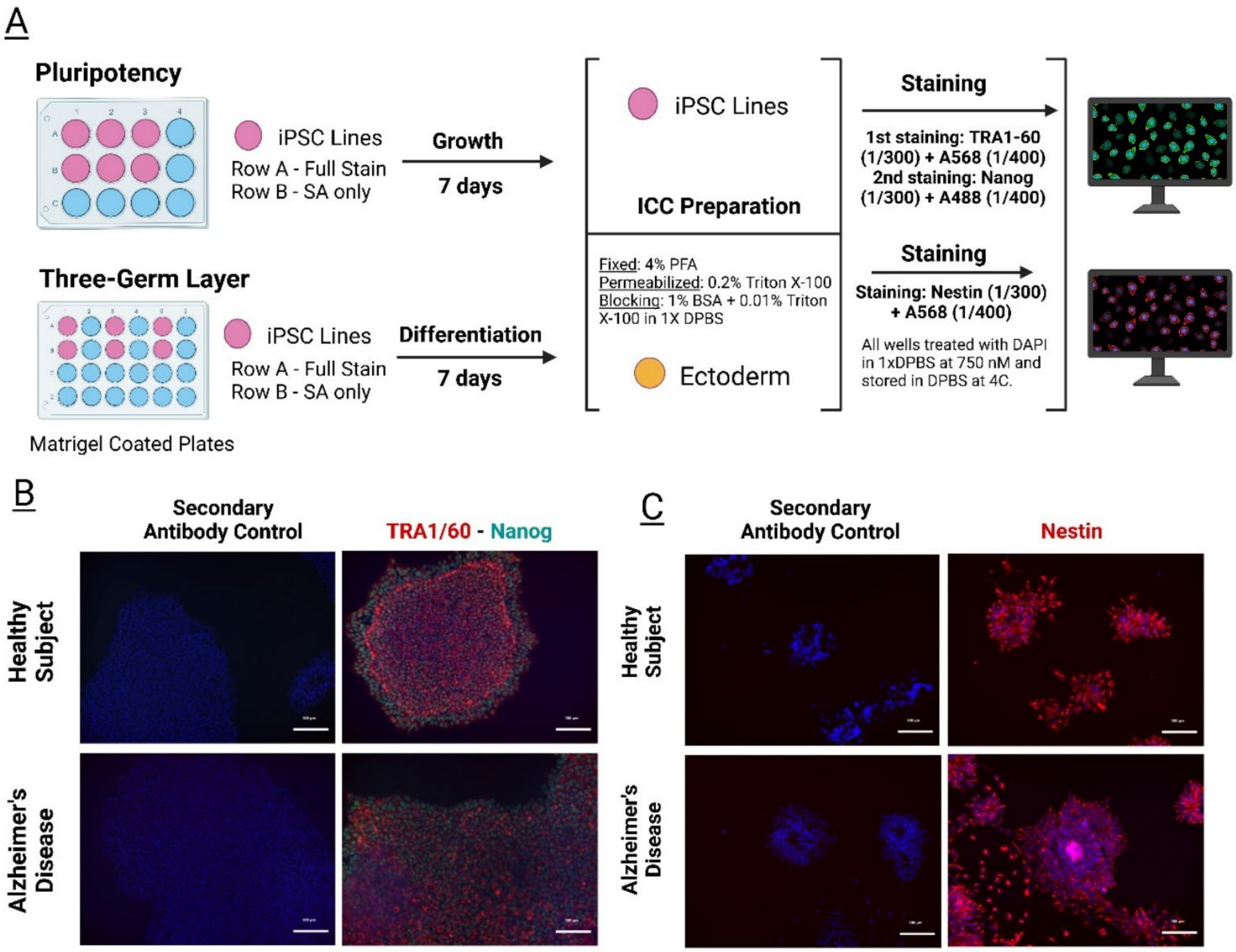

Supplementary Figure 18: QC of utilized IPSCs. A) Depiction of experimental paradigm to validate the pluripotency and ectodermal differentiating capability of IPSCs. This experiment was completed once for each IPSC lines, in a total of 4 cell lines (n=4). **B)** We stained IPSCs for TRA1/60 and Nanog. **C)** We stained differentiated IPSCs with Nestin. (Supplementary Figure 18/panel A Created with BioRender.com released under a Creative Commons Attribution-NonCommercial-NoDerivs license).

Supplementary Figure 19

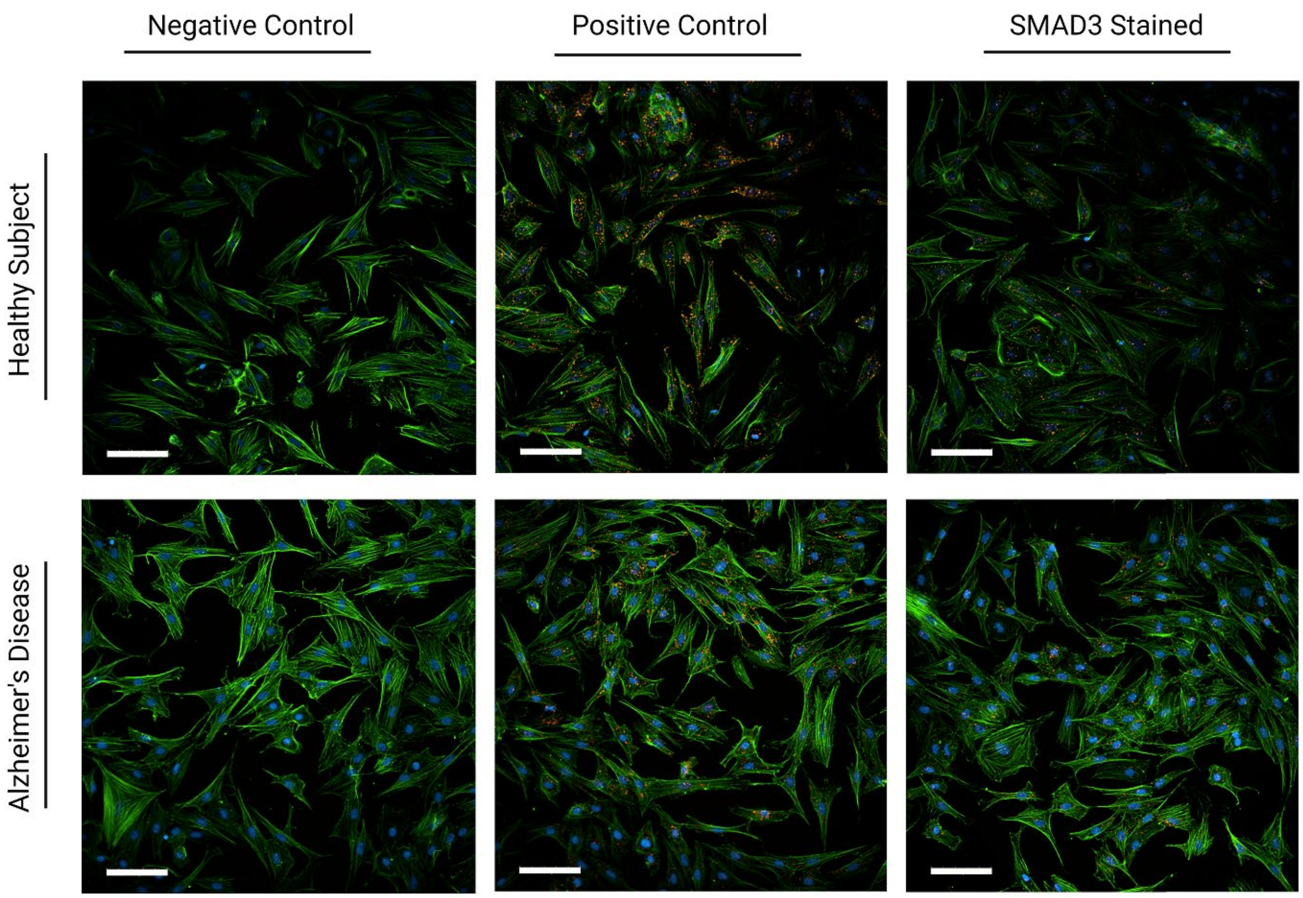

Supplementary Figure 19: Validation of *SMAD3* gene expression through RNAscope at iPSC derived pericytes. Negative Control: Bacterial *dapb*, Positive Control: *PPIB*, Orange: *SMAD3, Green*: Actin, Blue: Nuclei (DAPI). The white scale bar in each panel equals 100 µm.

Supplementary Figure 20

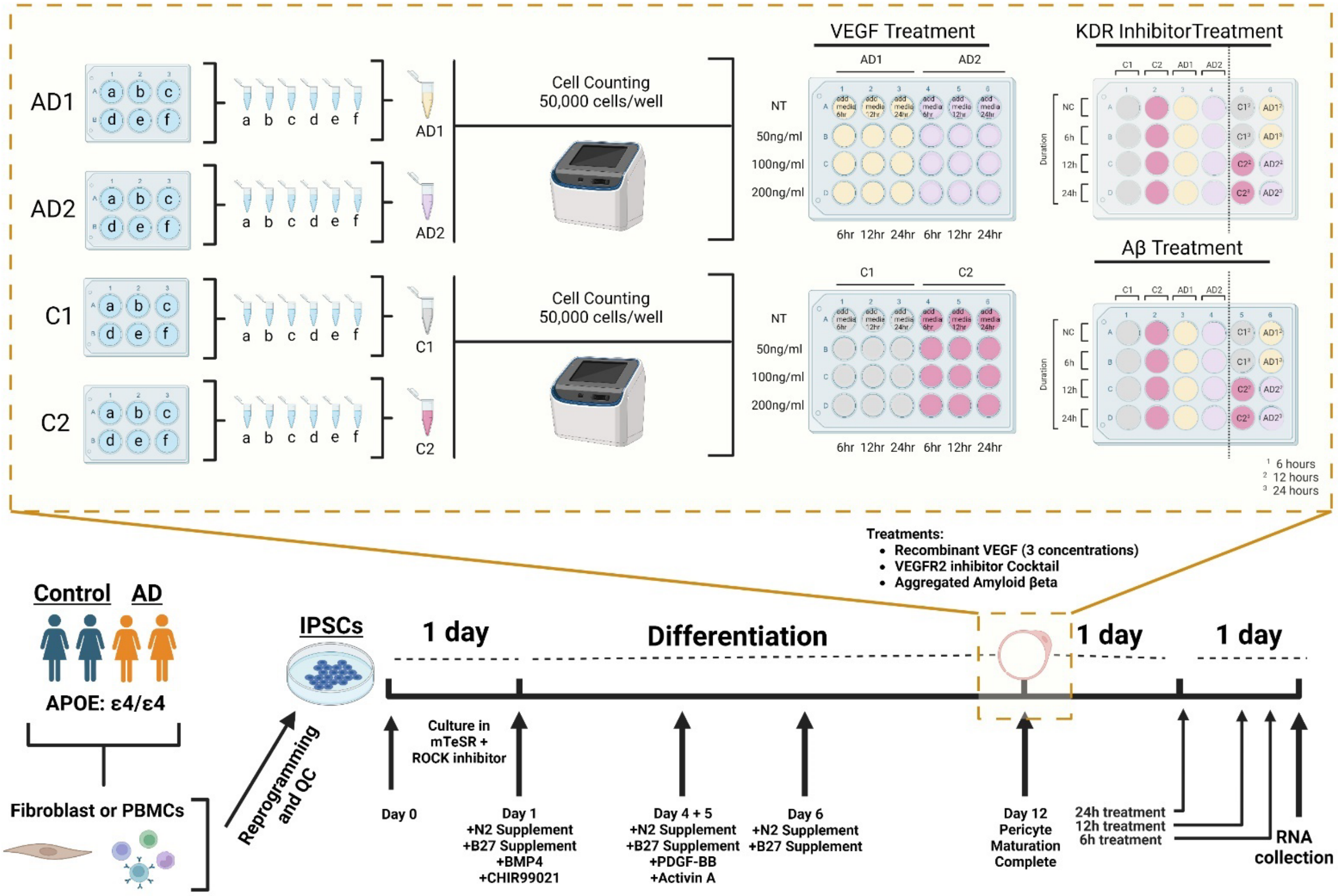

Supplementary Figure 20: Experimental design for the treatment of IPSC derived pericytes. The flowchart outlines the iPSC experimental design. Four patient-derived iPSC lines (n_{AD}=2, $n_{CONTROL}$=2) were used to differentiate into pericytes. Each patient derived IPSC was seeded on all wells of a 6 well plate. Seeded cells were differentiated into pericytes using established protocols as depicted. Cells in each well is accounted as one technical replicate and merged into one single tube to a total of 6 tubes. Collected cells were reseeded onto a 24 well plate for treatment experiments. Concentrations that were used for each stimulant were as follows: 50 ng/mL, 100 ng/mL, 200 ng/mL recombinant VEGF; 250 nM aggregated Aβ; and VEGFR2 inhibitor cocktail that contains (Semaxanib SU5416 (10 uM, SelleckChem S2845), Tivozanib AV- 951 (10 uM, SelleckChem S1207), and ZM 306416 (10 uM, SelleckChem, S2897). Treatment durations were as follows for each treatment: 6 hours, 12 hours, and 24 hours. Following treatment, RNA was collected and expression of selected genes were checked via qPCR. (Supplementary Figure 20 Created with BioRender.com released under a Creative Commons Attribution-NonCommercial-NoDerivs license)).

Supplementary Figure 21

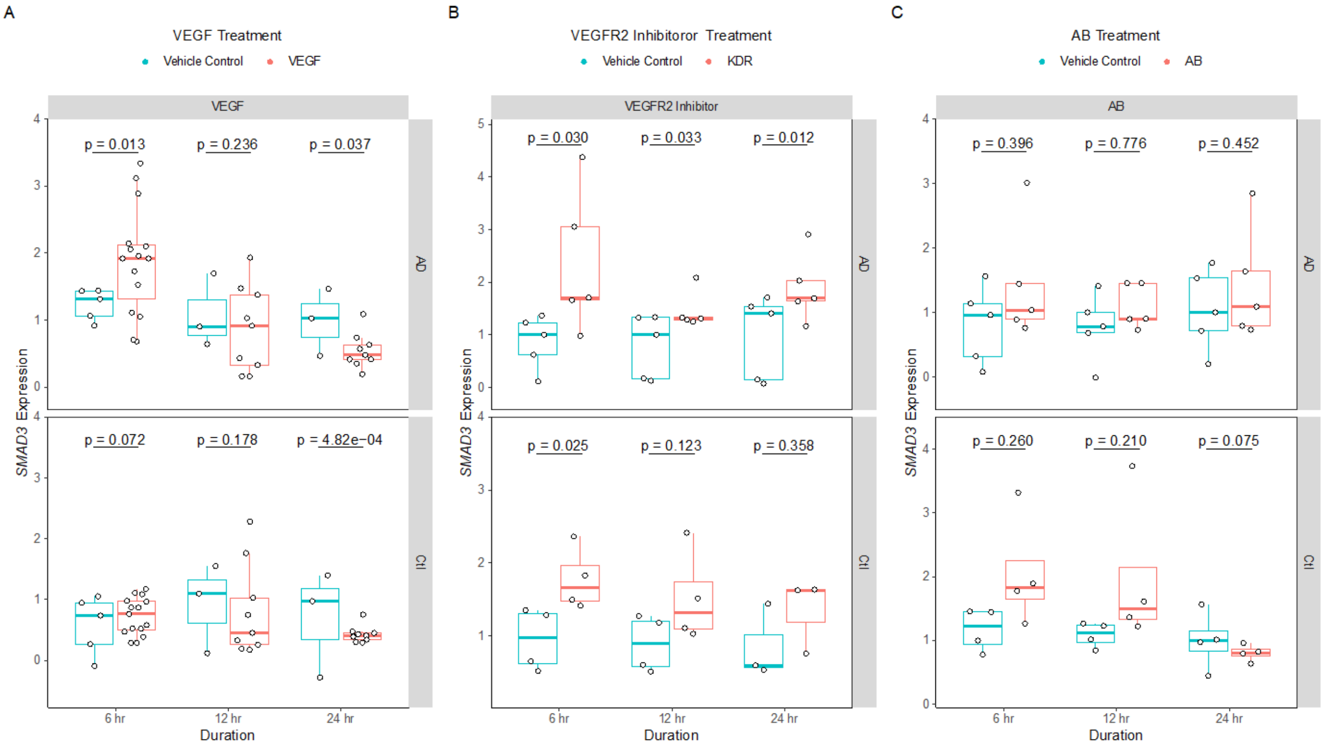

Supplementary Figure 21: Diagnosis stratified response of IPSC derived pericytes towards A) VEGF, B) VEGFR2 inhibitor and C) aggregated Aβ. Source data are provided as a Source Data file.

Supplementary Figure 22

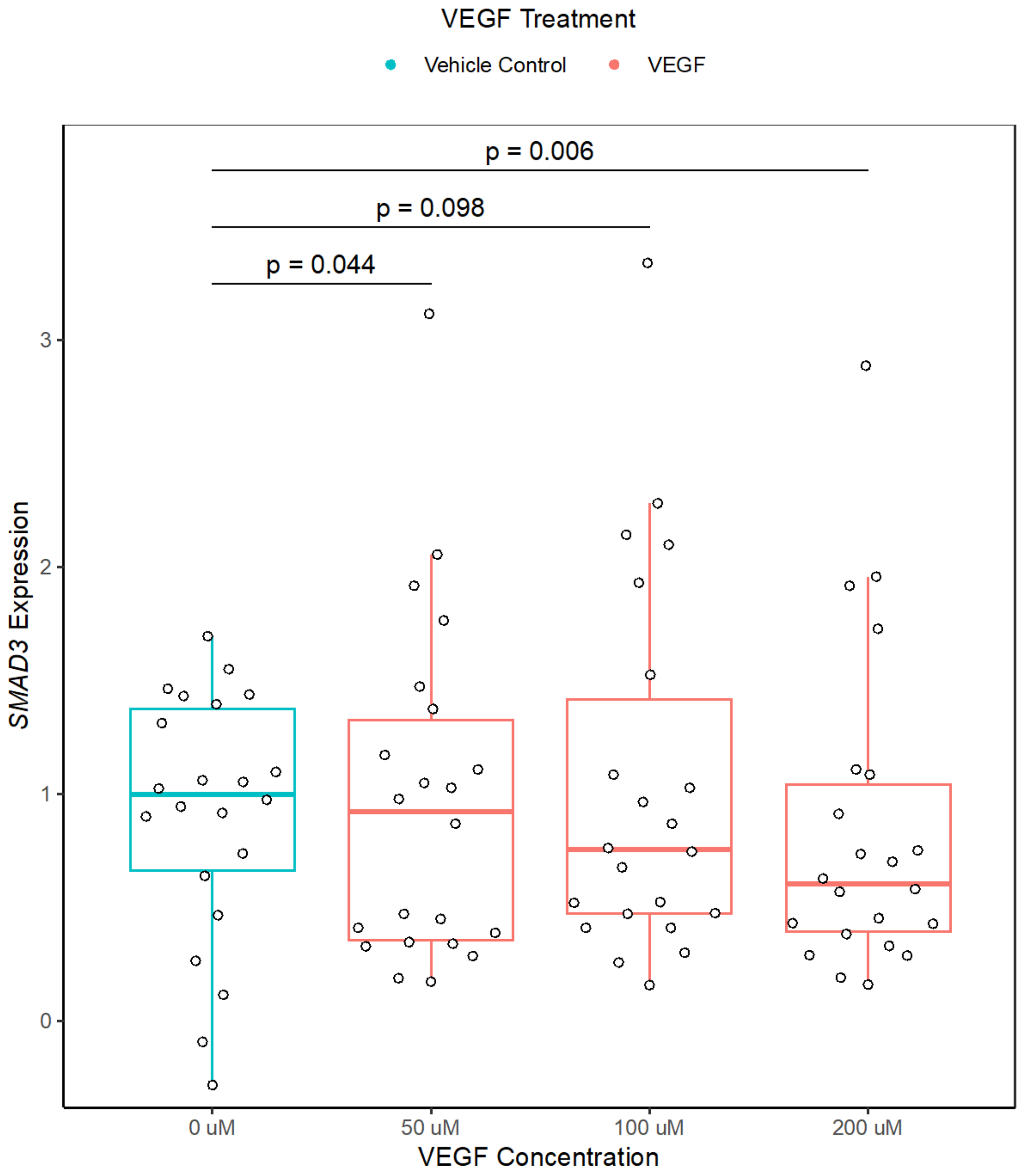

Supplementary Figure 22: Pericyte SMAD3 levels at different VEGF treatment concentrations. All timepoints from treatment with VEGF are compiled together and checked against untreated controls. 50 ng/mL and 100 ng/mL VEGF treatment caused significant downregulation of SMAD3 expression in iPSC derived pericytes compared to control conditions ($p<0.05$). Source data are provided as a Source Data file.

Supplementary Figure 23

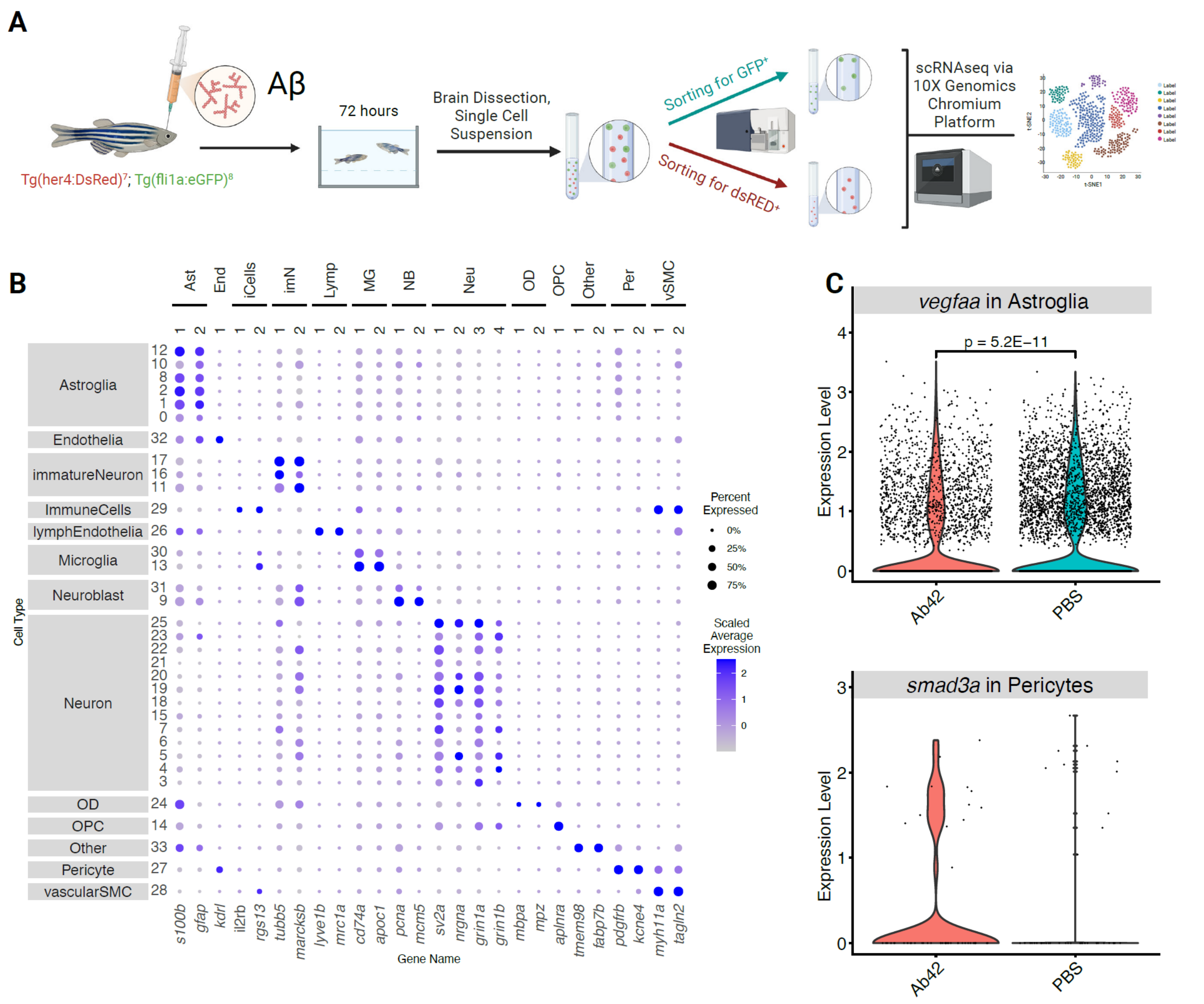

Supplementary Figure 23: Validation of human snRNAseq results on transgenic zebrafish. We confirmed that VEGF regulates SMAD3 and this signaling is altered in zebrafish AD model. A) Amyloid toxicity was induced in the adult telencephalon of double reporter transgenic zebrafish line – Tg(*her4*:dsRED) and Tg(*fli1*:EGFP) as established. B) Single cell clusters were clustered on UMAP and annotated based on marker gene expression. C) Injection of amyloid caused increased expression of smad3a in pericytes and decreased expression in astroglia cluster through scRNAseq. (Supplementary Figure 23/Panel A Created with BioRender.com released under a Creative Commons Attribution-NonCommercial-NoDerivs license).

Supplementary Figure 24

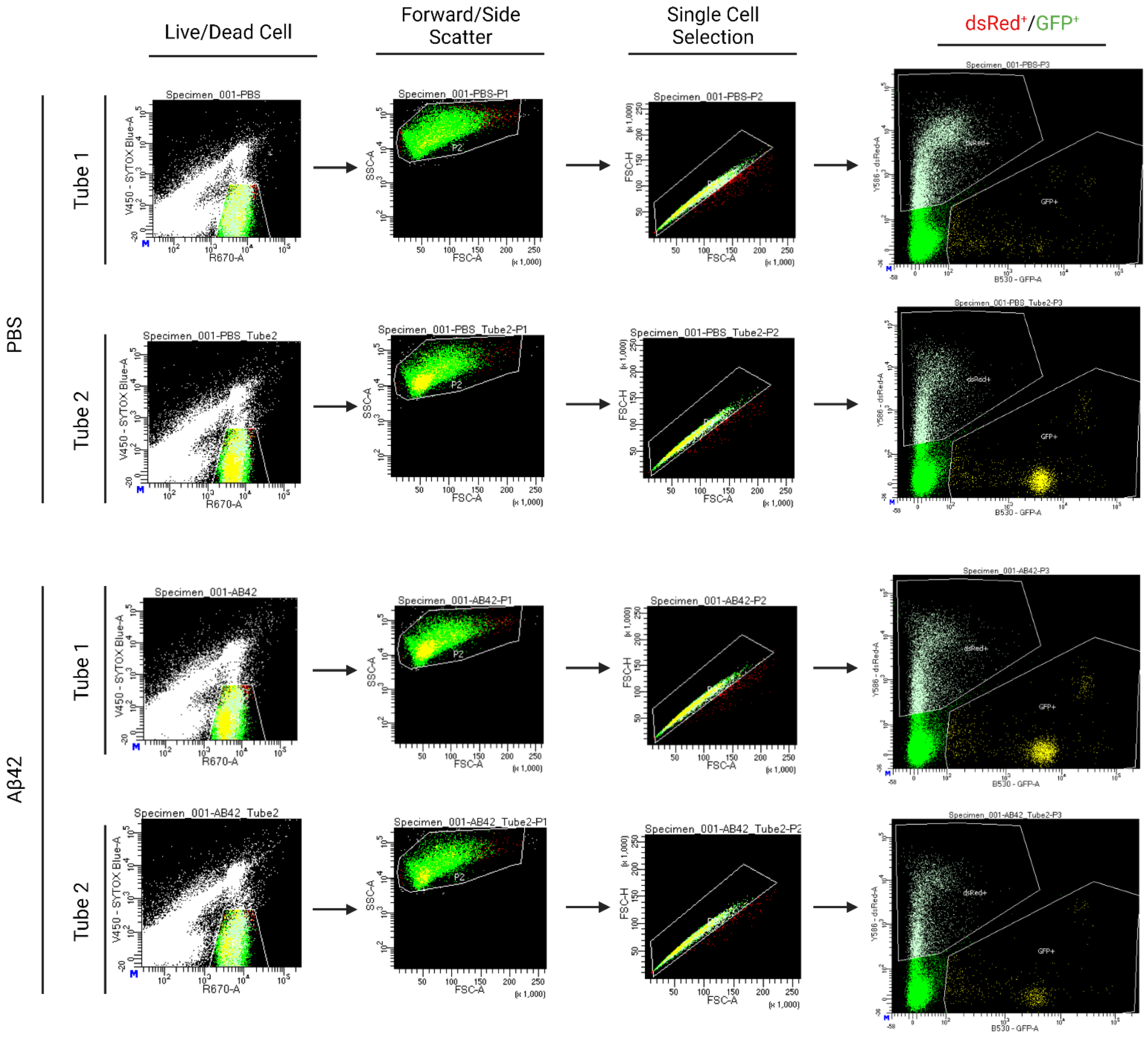

Supplementary Figure 24: FACS Gating strategy of zebrafish brain cells. Dot plots demonstrate gating strategy to obtain GFP$^+$ and dsRED$^+$ populations from PBS and Aβ42 injected Tg(*her4*:dsRED) and Tg(*fli1*:EGFP) zebrafish model. These cells were used to obtain scRNAseq profile from zebrafish brain cells.

Supplementary Figure 25

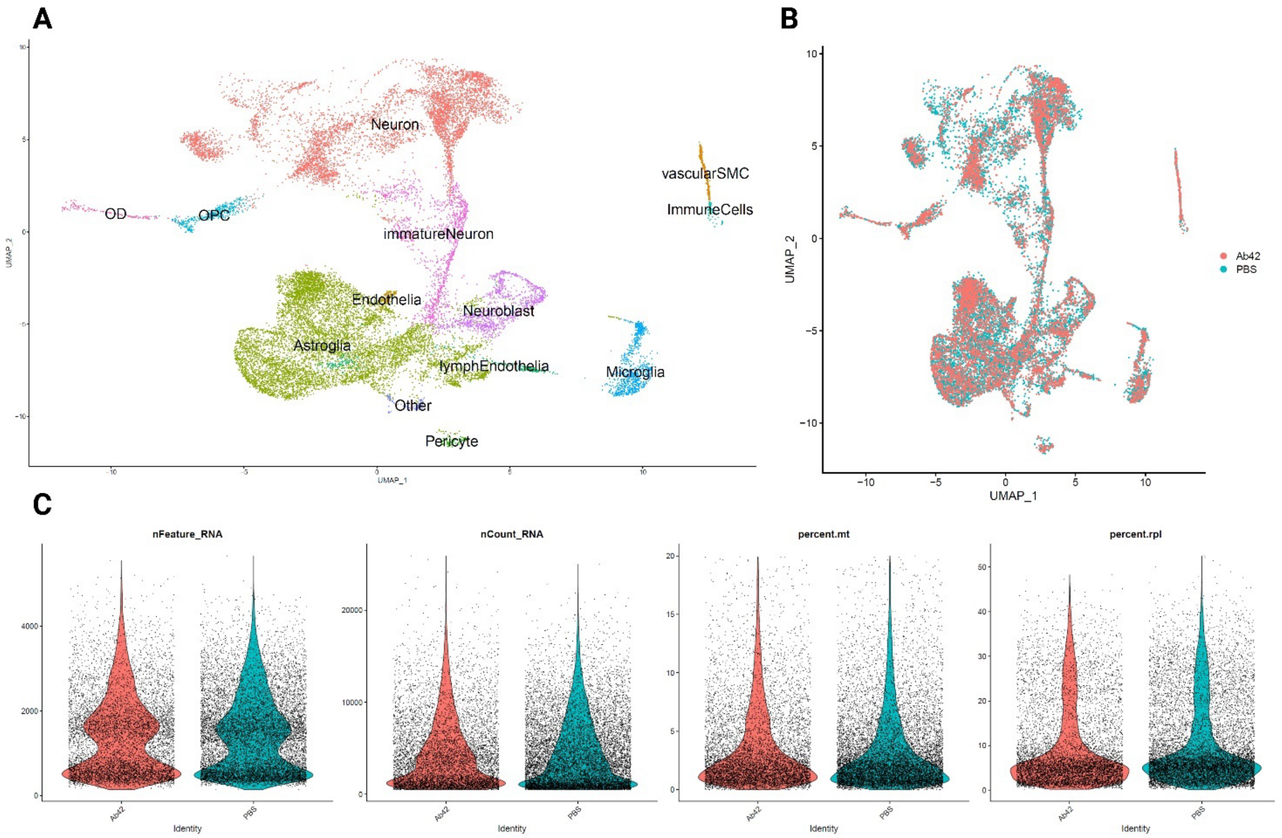

Supplementary Figure 25: QC Results of Aβ and PBS treated transgenic zebrafish results. A) Cell clusters displayed on UMAP reduced dimension space, annotated as brain cell types. Transcriptomic profile of major and minor brain cell types was obtained: Neurons, immature neurons, neuroblast, oligodendrocytes (OD), oligodendrocyte precursor cells (OPCs), endothelia, astroglia, microglia, and pericytes. **B)** Distribution of cell types according to treatment status (Aβ42 or PBS as vehicle) on UMAP reduced dimension space. **C)** Density plots demonstrate cell distributions in two treatment groups according to single cell transcriptomic parameters (nFeature_RNA: the number of genes detected in each cell; nCount_RNA: Total number of molecules detected within a cell; percent.mt: The percentage of reads that map to the mitochondrial genome; percent.rpl: The percentage of reads that map to the ribosomal protein L (rpl) genes).

Supplementary Figure 26

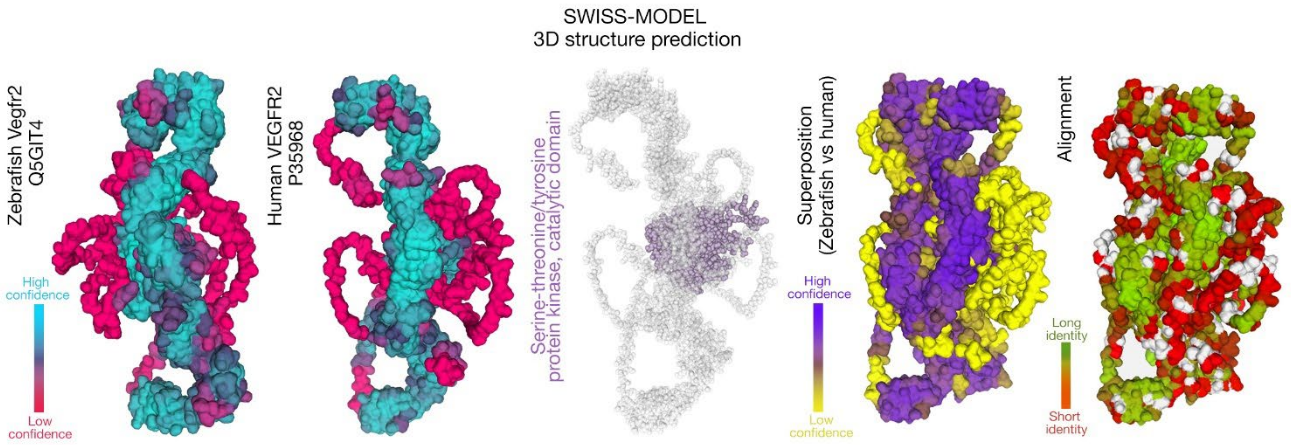

Supplementary Figure 26: Structural comparison of VEGFR2 in human and zebrafish. We made a structural comparison of human VEGFR2 with zebrafish vegfr2 using Swiss 3D Model prediction algorithm. Our *in silico* analysis predicts that the catalytic domain is highly conserved in 3D and drugs targeting this domain would be effective for both species.

Supplementary Figure 27

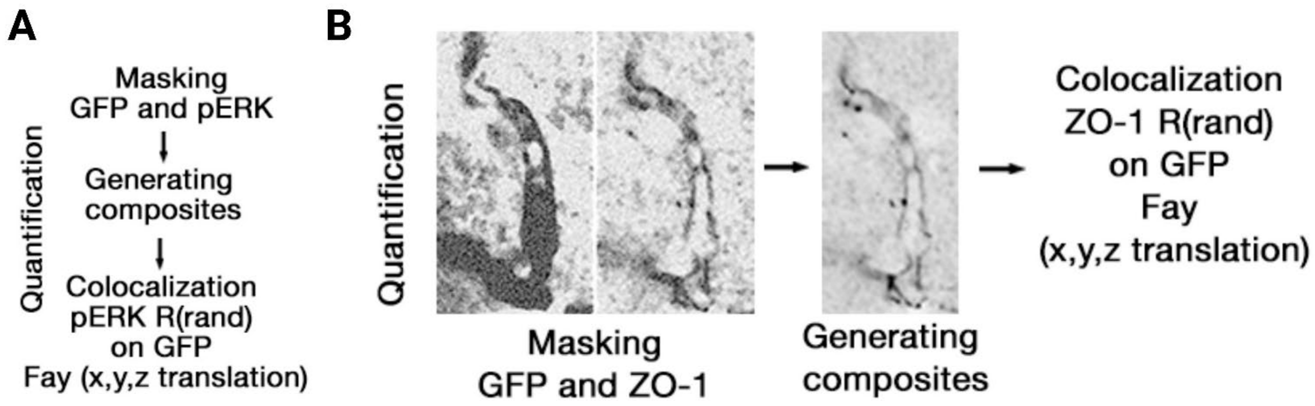

Supplementary Figure 27: Colocalization of A) pERK and B) ZO-1 on GFP fay is visualized. In order to quantify the colocalization of markers, ImageJ software's colocalization module was used to generate two-channel composites, R(and) colocalization analyses, and Fay translation into correlation values. Pairwise comparisons were performed with unpaired parametric t test with Welch's correction.

Supplementary Figure 28

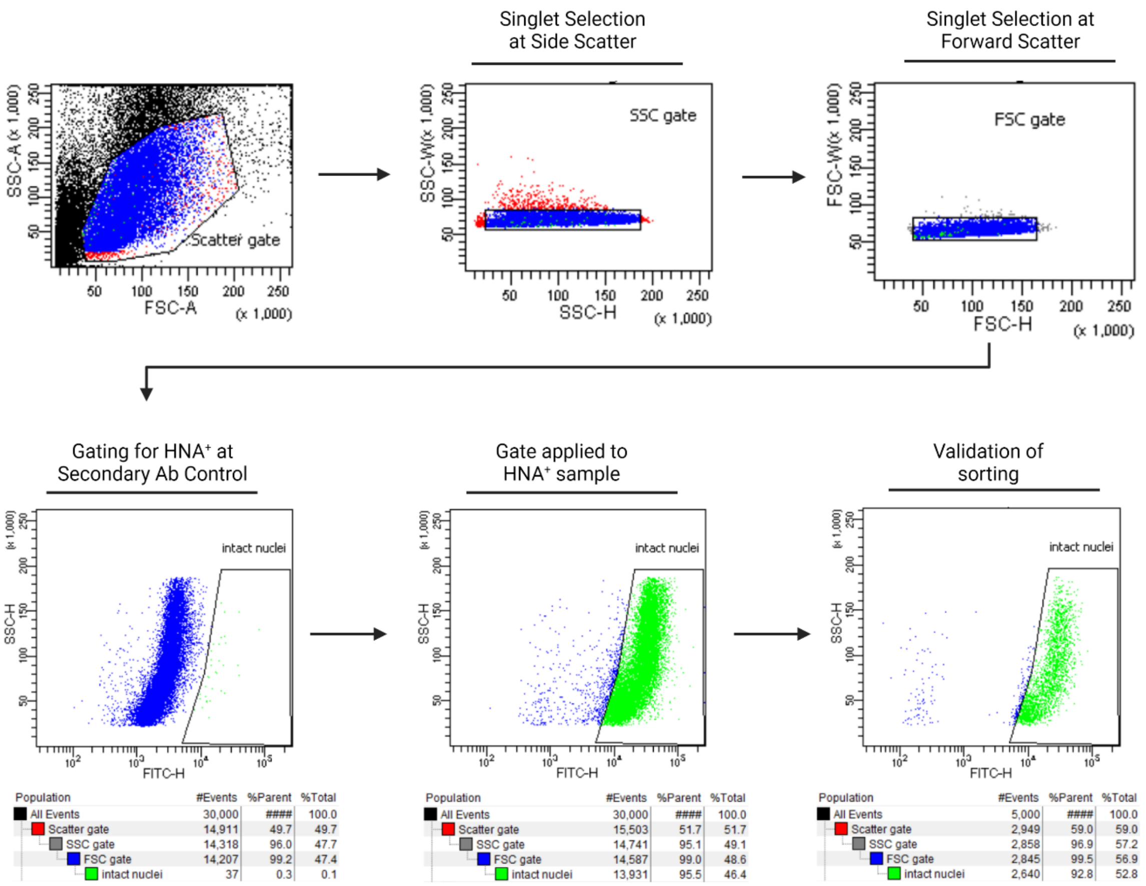

Supplementary Figure 28: **FANS Gating strategy of intact nuclei from frozen human brain.** Dot plots demonstrate gating strategy to obtain HNA⁺ population from nuclear samples. Isolation of nuclei were performed as previously established and purified via FANS as depicted. Sorted nuclei were directly used to generate snRNAseq data, or to seed for validation experiments such as RNAscope and qPCR.

Supplementary Figure 29

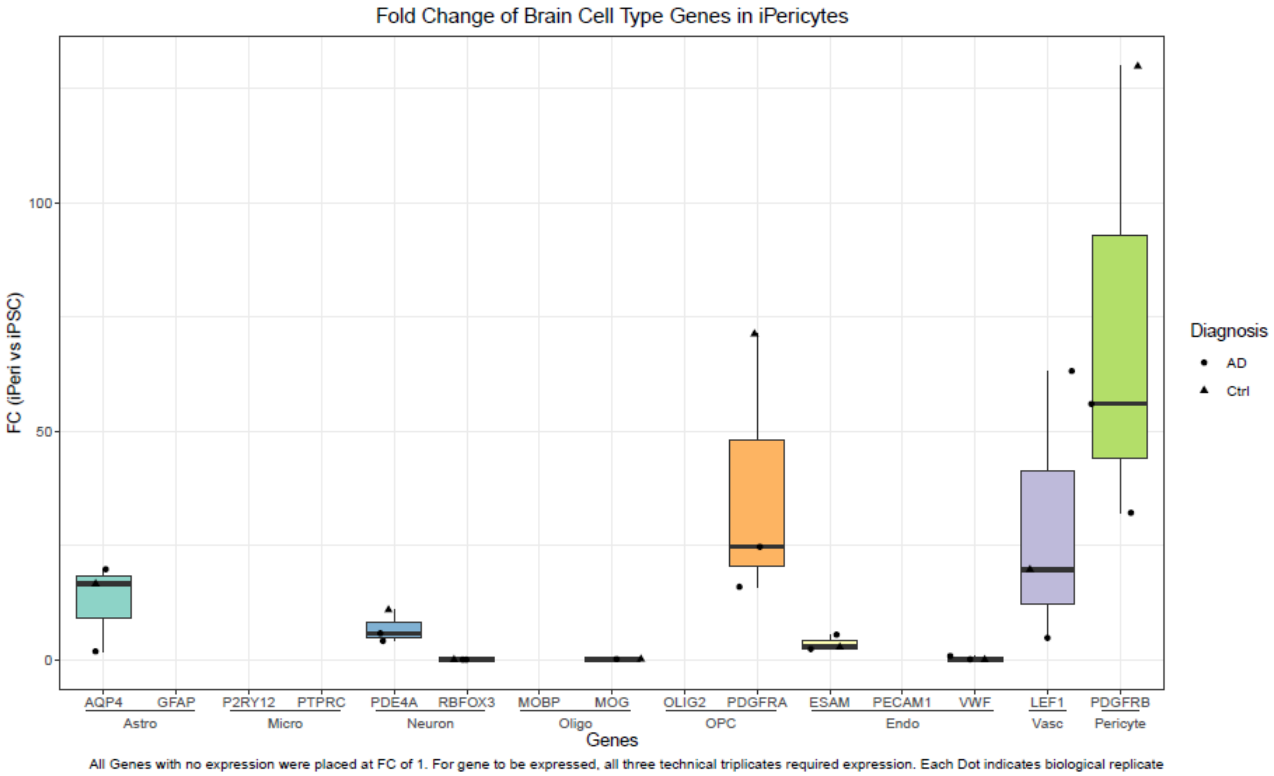

Supplementary Figure 29: Fold change in gene expression profile of iPSC-derived pericytes. To confirm the identity of differentiated cells' profile, expression of brain cell type marker genes was analyzed via qPCR: *AQP4* and *GFAP* for astrocytes; *P2RY12* and *PTPRC* for microglia; *PDE4A* and *RBFOX3* for neurons; *MOBP* and *MOG* for oligodendrocytes; *OLIG2* and *PDGFRA* for OPCs; *ESAM*, *PECAM1* and *VWF* for endothelia; *PDGFRB* for pericytes; and *LEF1* for vascular cells. Source data are provided as a Source Data file.

Supplementary Figure 30

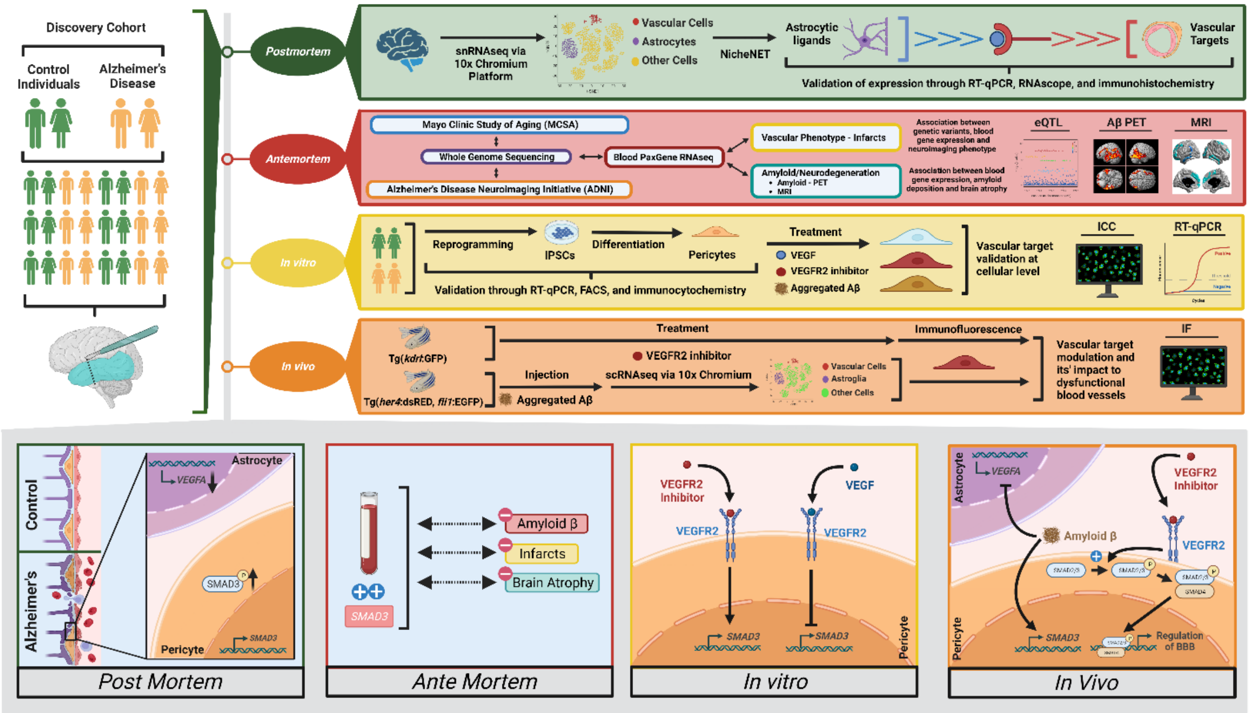

Supplementary Figure 30: Graphical Abstract. Using single nucleus transcriptome from brain tissue of donors with Alzheimer's disease (AD) and elderly controls, we dissected the transcriptional landscape of vascular cells (red) and astrocytes (purple) that form the gliovascular unit (GVU) at the blood brain barrier (BBB) which is disrupted in AD. We performed tiered complementary studies to discover and validate astrocytic ligand – vascular target pairs that contribute to this BBB disruption in AD. *Postmortem:* Our human brain snRNAseq study discovered perturbed vascular and astrocytic transcript pairs, of which pericytic *SMAD3* (up in AD) and astrocytic *VEGFA* (down in AD) were prioritized. These findings were validated with orthogonal quantitative PCR, RNAscope and immunohistochemistry studies and replicated in external human brain snRNAseq data. *Antemortem*: Using *SMAD3* locus genetic variants, we performed expression QTL (eQTL) with blood *SMAD3* levels and associations with neuroimaging phenotypes relevant to AD. Genetic variants associated with higher blood *SMAD3* levels also associated with lower brain infarcts. Further, higher blood *SMAD3* levels associated with less amyloid and cortical atrophy on antemortem imaging. *In Vitro:* We validated *VEGFA-SMAD3* interactions in human iPSC-derived pericytes. Treatment of human pericytes with VEGF (encoded by *VEGFA*) reduces *SMAD3*, and blocking VEGF signalling increases *SMAD3*. ***In Vivo*:** To determine impact of *VEGFA-SMAD3* interactions on the blood-brain-barrier experimentally, we utilized a well-established zebrafish model. Injection of amyloid β42 in this model decreased *vegfaa* (zebrafish ortholog to human *VEGFA*) expression in astroglia. Blocking *vegfaa* signaling pharmacologically increased phosphorylated Smad3, the active form of this signalling molecule and importantly also impaired blood-brain-barrier integrity. Collectively, our findings highlight the vast transcriptome changes in AD at the GVU, prioritize perturbed pericytic *SMAD3*-astrocytic *VEGFA* interactions with cross species experimental validations that provide a mechanistic avenue that contributes to BBB disintegrity in AD.
(Supplementary Figure 30 Created with BioRender.com released under a Creative Commons Attribution-NonCommercial-NoDerivs license).

References:

1 Schilling, S. *et al.* APOE genotype and MRI markers of cerebrovascular disease: systematic review and meta-analysis. *Neurology* **81**, 292-300 (2013). https://doi.org:10.1212/WNL.0b013e31829bfda4

2 Etherton, M. R. *et al.* Sex-specific differences in white matter microvascular integrity after ischaemic stroke. *Stroke Vasc Neurol* **4**, 198-205 (2019). https://doi.org:10.1136/svn-2019-000268

3 Wang, M. *et al.* Guidelines for bioinformatics of single-cell sequencing data analysis in Alzheimer's disease: review, recommendation, implementation and application. *Mol Neurodegener* **17**, 17 (2022). https://doi.org:10.1186/s13024-022-00517-z

4 Lau, S.-F., Cao, H., Fu, A. K. Y. & Ip, N. Y. Single-nucleus transcriptome analysis reveals dysregulation of angiogenic endothelial cells and neuroprotective glia in Alzheimer's disease. *Proceedings of the National Academy of Sciences* **117**, 25800-25809 (2020). https://doi.org:10.1073/pnas.2008762117

5 Browaeys, R., Saelens, W. & Saeys, Y. NicheNet: modeling intercellular communication by linking ligands to target genes. *Nature Methods* **17**, 159-162 (2020). https://doi.org:10.1038/s41592-019-0667-5

6 Saykin, A. J. *et al.* Genetic studies of quantitative MCI and AD phenotypes in ADNI: Progress, opportunities, and plans. *Alzheimers Dement* **11**, 792-814 (2015). https://doi.org:10.1016/j.jalz.2015.05.009

ACKNOWLEDGEMENT LIST FOR ADNI PUBLICATIONS

The Data and Publications Committee, in keeping with the publication policies adopted by the ADNI Steering Committee, here provide lists for standardized acknowledgement. The list consists of three parts: I. ADNI Infrastructure Investigators and Site Investigators, II. DOD ADNI Infrastructure Investigators and Site Investigators and III. ADNI Depression Infrastructure Investigators and Site Investigators. Infrastructure Investigators represent the names responsible for leadership and infrastructure. Site Investigators represent the names of individuals at each recruiting site. All papers, including methodological papers, should have an acknowledgement list that consists of Infrastructure Investigators plus the FULL list.

I. ADNI I, GO, II and III

Part A: Leadership and Infrastructure

Principal Investigator
Michael W. Weiner, MD — University of California, San Francisco

ATRI PI and Director of Coordinating Center Clinical Core
Paul Aisen, MD — University of Southern California
Co PI of Clinical Core Ronald Petersen, MD, PhD — Mayo Clinic, Rochester

Executive Committee

Michael W. Weiner, MD	University of California, San Francisco
Paul Aisen, MD	University of Southern California
Ronald Petersen, MD, PhD	Mayo Clinic, Rochester
Clifford R. Jack, Jr., MD	Mayo Clinic, Rochester
William Jagust, MD	University of California, Berkeley
John Q. Trojanowki, MD, PhD	University of Pennsylvania
Arthur W. Toga, PhD	University of Southern California
Laurel Beckett, PhD	University of California, Davis
Robert C. Green, MD, MPH	Brigham and Women's Hospital/Harvard Medical School
Andrew J. Saykin, PsyD	Indiana University
John C. Morris, MD	Washington University St. Louis
Richard J. Perrin, MD, PhD	Washington University St. Louis
Leslie M. Shaw, PhD	University of Pennsylvania

ADNI External Advisory Board (ESAB)

Zaven Khachaturian, PhD	Prevent Alzheimer's Disease 2020 (Chair)
Maria Carrillo, PhD	Alzheimer's Association
William Potter, MD	National Institute of Mental Health
Lisa Barnes, PhD	Rush University
Marie Bernard, MD	NIA
Hector González	University of California, San Diego
Carole Ho	Denali Therapeutics
John K. Hsiao, MD	NIH
Jonathan Jackson, PhD	Massachusetts General Hospital
Eliezer Masliah, MD	NIA
Donna Masterman, MD	Biogen
Ozioma Okonkwo, PhD	University of Wisconsin, Madison

Richard Perrin, MD	Washington University
Laurie Ryan, PhD	NIA
Nina Silverberg, PhD	NIA

ADNI 3 Private Partner Scientific Board (PPSB)

Adam Fleisher, MD	Eli Lilly (Chair)

Administrative Core - Northern California Institute for Research & Education (NCIRE / The Vererans Health Research Institute)

Michael W. Weiner, MD	University of California, San Francisco
Diana Truran Sacrey,	NCIRE / The Vererans Health Research Institute
Juliet Fockler,	University of California, San Francisco
Cat Conti, BA	NCIRE / The Vererans Health Research Institute
Dallas Veitch, PhD	NCIRE / The Vererans Health Research Institute
John Neuhaus, PhD	University of California, San Francisco
Chengshi Jin, PhD	University of California, San Francisco
Rachel Nosheny, PhD	University of California, San Francisco
Miriam Ashford, PhD	NCIRE / The Vererans Health Research Institute
Derek Flenniken,	NCIRE / The Vererans Health Research Institute
Adrienne Kormos,	NCIRE / The Vererans Health Research Institute

Data and Publications Committee

Robert C. Green, MD, MPH	BWH/HMS (Chair)

Resource Allocation Review Committee

Tom Montine, MD, PhD	University of Washington (Chair)
Cat Conti, BA	NCIRE / The Vererans Health Research Institute

Clinical Core Leaders and Key Personnel

Ronald Petersen, MD, PhD	Mayo Clinic, Rochester (Core PI)
Paul Aisen, MD	University of Southern California (Core PI)
Michael Rafii, MD, PhD	University of Southern California
Rema Raman, PhD	University of Southern California
Gustavo Jimenez, MBS	University of Southern California
Michael Donohue, PhD	University of Southern California
Devon Gessert, BS	University of Southern California
Jennifer Salazar, MBS	University of Southern California
Caileigh Zimmerman, MS	University of Southern California
Yuliana Cabrera, BS	University of Southern California
Sarah Walter, MSc	University of Southern California
Garrett Miller, MS	University of Southern California
Godfrey Coker, MBA, MPH	University of Southern California
Taylor Clanton, MPH	University of Southern California
Lindsey Hergesheimer, BS	University of Southern California
Stephanie Smith, BS	University of Southern California
Olusegun Adegoke, MSc	University of Southern California
Payam Mahboubi, MPH	University of Southern California
Shelley Moore, BA	University of Southern California
Jeremy Pizzola, BA	University of Southern California
Elizabeth Shaffer, BS	University of Southern California

Brittany Sloan, BA
University of Southern California

Biostatistics Core Leaders and Key Personnel

Laurel Beckett, PhD	University of California, Davis (Core PI)
Danielle Harvey, PhD	University of California, Davis
Michael Donohue, PhD	University of Southern California

MRI Core Leaders and Key Personnel

Clifford R. Jack, Jr., MD	Mayo Clinic, Rochester (Core PI)
Arvin Forghanian-Arani, PhD	Mayo Clinic
Bret Borowski, RTR	Mayo Clinic
Chad Ward,	Mayo Clinic
Christopher Schwarz, PhD	Mayo Clinic
David Jones, MD	Mayo Clinic
Jeff Gunter, PhD	Mayo Clinic
Kejal Kantarci, MD	Mayo Clinic
Matthew Senjem, MS	Mayo Clinic
Prashanthi Vemuri, PhD	Mayo Clinic
Robert Reid, PhD	Mayo Clinic
Nick C. Fox, MD	University College London
Ian Malone, PhD	University College London
Paul Thompson, PhD	University of Southern California School of Medicine
Sophia I. Thomopoulos, BS	University of Southern California School of Medicine
Talia M. Nir, PhD	University of Southern California School of Medicine
Neda Jahanshad, PhD	University of Southern California School of Medicine
Charles DeCarli, MD	University of California, Davis
Alexander Knaack, MS	University of California, Davis
Evan Fletcher, PhD	University of California, Davis
Danielle Harvey, PhD	University of California, Davis
Duygu Tosun-Turgut, PhD	University of California, San Francisco
Stephanie Rossi Chen, BA.	NCIRE / The Vererans Health Research Institute
Mark Choe, BS	NCIRE / The Vererans Health Research Institute
Karen Crawford,	University of Southern California School of Medicine
Paul A. Yushkevich, PhD	University of Pennsylvania
Sandhitsu Das, PhD	University of Pennsylvania

PET Core Leaders and Key Personnel

William Jagust, MD	University of California, Berkeley (Core PI)
Robert A. Koeppe, PhD	University of Michigan
Eric M. Reiman, MD	Banner Alzheimer's Institute
Kewei Chen, PhD	Banner Alzheimer's Institute
Chet Mathis, MD	University of Pittsburgh
Susan Landau, PhD	University of California, Berkeley

Neuropathology Core Leaders and Key Personnel

John C. Morris, MD	Washington University St. Louis
Richard Perrin MD	Washington University St. Louis
Nigel J. Cairns, PhD, FRCPath	Washington University St. Louis—Past Investigator
Erin Householder, MS	Washington University St. Louis

Erin Franklin, MS — Washington University St. Louis
Haley Bernhardt, BA, R. EEG T — Washington University St. Louis
Lisa Taylor-Reinwald, BA, HTL (ASCP) – Past Investigator — Washington University St. Louis

Biomarkers Core Leaders and Key Personnel

Leslie M. Shaw, PhD	Perelman School of Medicine, University of Pennsylvania (co-PI)
John Q. Trojanowki, MD, PhD	Perelman School of Medicine, University of Pennsylvania (co-PI)
Magdalena Korecka, PhD	Perelman School of Medicine, University of Pennsylvania
Michal Figurski, PhD	Perelman School of Medicine, University of Pennsylvania

Informatics Core Leaders and Key Personnel

Arthur W. Toga, PhD	University of Southern California (Core PI)
Karen Crawford	University of Southern California
Scott Neu, PhD	University of Southern California

Genetics Core Leaders and Key Personnel

Andrew J. Saykin, PsyD	Indiana University School of Medicine (Core PI)
Kwangsik Nho, PhD	Indiana University School of Medicine
Shannon L. Risacher, PhD	Indiana University School of Medicine
Liana G. Apostolova, MD	Indiana University School of Medicine
Li Shen, PhD	UPenn School of Medicine
Tatiana M. Foroud, PhD	NCRAD/Indiana University School of Medicine
Kelly Nudelman, PhD	NCRAD/Indiana University School of Medicine
Kelley Faber, MS, CCRC	NCRAD/Indiana University School of Medicine
Kristi Wilmes, MS, CCRP	NCRAD/Indiana University School of Medicine

Initial Concept Planning & Development

Michael W. Weiner, MD	University of California, San Francisco
Leon Thal, MD – Past Investigator	University of California, San Diego
Zaven Khachaturian, PhD	Prevent Alzheimer's Disease 2020

NIA

John K. Hsiao, MD	National Institute on Aging

<u>Part B: Investigators By Site</u>

Oregon Health & Science University:
Lisa C. Silbert, MD
Betty Lind, BS
Rachel Crissey
Jeffrey A. Kaye, MD, A – Past Investigator
Raina Carter, BA – Past Investigator
Sara Dolen, BS – Past Investigator
Joseph Quinn, MD – Past Investigator

University of Southern California:
Lon S. Schneider, MD
Sonia Pawluczyk, MD
Mauricio Becerra, MD
Liberty Teodoro, RN
Karen Dagerman, MS
Bryan M. Spann, DO, PhD – Past Investigator

University of California – San Diego:
James Brewer, MD, PhD
Helen Vanderswag, RN

Adam Fleisher, MD – Past Investigator

University of Michigan:
Jaimie Ziolkowski, MA, BS, TLLP
Judith L. Heidebrink, MD, MS
Lisa Zbizek-Nulph, MS
Joanne L. Lord, LPN, BA, CCRC – Past Investigator
Lisa Zbizek-Nulph, MS, CCRP

Mayo Clinic, Rochester:
Ronald Petersen, MD, PhD
Sara S. Mason, RN
Colleen S. Albers, RN
David Knopman, MD
Kris Johnson, RN

Baylor College of Medicine:
Javier Villanueva-Meyer, MD
Valory Pavlik, PhD
Nathaniel Pacini, MA
Ashley Lamb, MA
Joseph S. Kass, MD, LD, FAAN
Rachelle S. Doody, MD, PhD – Past Investigator
Victoria Shibley, MS – Past Investigator
Munir Chowdhury, MBBS, MS – Past Investigator
Susan Rountree, MD – Past Investigator
Mimi Dang, MD – Past Investigator

Columbia University Medical Center:
Yaakov Stern, PhD
Lawrence S. Honig, MD, PhD
Akiva Mintz, MD, PhD

Washington University, St. Louis:
Beau Ances, MD, PhD, MSc
John C. Morris, MD
David Winkfield, BS
Maria Carroll, RN, MSN, GCNS-BC
Georgia Stobbs-Cucchi, RN, CCRP—Past Investigator
Angela Oliver, RN, BSN, MSG – Past Investigator
Mary L. Creech, RN, MSW – Past Investigator
Mark A. Mintun, MD – Past Investigator
Stacy Schneider, APRN, BC, GNP – Past Investigator

University of Alabama - Birmingham:
David Geldmacher, MD
Marissa Natelson Love, MD
Randall Griffith, PhD, ABPP – Past Investigator
David Clark, MD – Past Investigator
John Brockington, MD – Past Investigator
Daniel Marson, JD, PhD – Past Investigator

Mount Sinai School of Medicine:
Hillel Grossman, MD
Martin A. Goldstein, MD
Jonathan Greenberg, BA
Effie Mitsis, PhD – Past Investigator

Rush University Medical Center:
Raj C. Shah, MD
Melissa Lamar, PhD
Patricia Samuels

Wien Center:
Ranjan Duara, MD
Maria T. Greig-Custo, MD
Rosemarie Rodriguez, PhD

Johns Hopkins University:
Marilyn Albert, PhD
Chiadi Onyike, MD
Leonie Farrington, RN
Scott Rudow, BS
Rottislav Brichko, BS
Stephanie Kielb, BS – Past Investigator

University of South Florida: USF Health Byrd Alzheimer's Institute:
Amanda Smith, MD
Balebail Ashok Raj, MD – Past Investigator
Kristin Fargher, MD – Past Investigator

New York University:
Martin Sadowski, MD, PhD
Thomas Wisniewski, MD
Melanie Shulman, MD
Arline Faustin, MD
Julia Rao, PhD
Karen M. Castro, BA
Anaztasia Ulysse, BA
Shannon Chen, BA
Mohammed O. Sheikh, MD – Past Investigator
Jamika Singleton-Garvin, CCRP – Past Investigator

Duke University Medical Center:
P. Murali Doraiswamy, MBBS, FRCP
Jeffrey R. Petrella, MD
Olga James, MD

Terence Z. Wong, MD
Salvador Borges-Neto, MD – Past Investigator

University of Pennsylvania:
Jason H. Karlawish, MD
David A. Wolk, MD
Sanjeev Vaishnavi, MD
Christopher M. Clark, MD – Past Investigator
Steven E. Arnold, MD – Past Investigator

University of Kentucky:
Charles D. Smith, MD

Gregory A. Jicha, MD, PhD
Riham El Khouli, MD
Flavius D. Raslau, MD

University of Pittsburgh:
Oscar L. Lopez, MD
MaryAnn Oakley, MA
Donna M. Simpson, CRNP, MPH

University of Rochester Medical Center:
Anton P. Porsteinsson, MD
Kim Martin, RN
Nancy Kowalski, MS, RNC
Melanie Keltz, RN
Bonnie S. Goldstein, MS, NP – Past Investigator
Kelly M. Makino, BS – Past Investigator
M. Saleem Ismail, MD – Past Investigator
Connie Brand, RN – Past Investigator

University of California Irvine IMIND:
Gaby Thai, MD
Aimee Pierce, MD
Beatriz Yanez, RN
Elizabeth Sosa, PhD
Megan Witbracht, PhD

University of Texas Southwestern Medical School:
Brendan Kelley, MD
Trung Nguyen, MD
Kyle Womack, MD
Dana Mathews, MD, PhD– Past Investigator
Mary Quiceno, MD– Past Investigator

Emory University:
Allan I. Levey, MD, PhD
James J. Lah, MD, PhD
Ihab Hajjar, MD
Janet S. Cellar, DNP, PMHCNS-BC – Past Investigator

University of Kansas, Medical Center:
Jeffrey M. Burns, MD
Russell H. Swerdlow, MD
William M. Brooks, PhD

University of California, Los Angeles:
Daniel H.S. Silverman, MD, PhD
Sarah Kremen, MD
Liana Apostolova, MD – Past Investigator
Kathleen Tingus, PhD – Past Investigator
Po H. Lu, PsyD – Past Investigator
George Bartzokis, MD – Past Investigator
Ellen Woo, PhD – Past Investigator
Edmond Teng, MD, PhD – Past Investigator

Mayo Clinic, Jacksonville:
Neill R Graff-Radford, MBBCH, FRCP (London)
Francine Parfitt, MSH, CCRC
Kim Poki-Walker, BA

Indiana University:
Martin R. Farlow, MD
Ann Marie Hake, MD – Past Investigator
Brandy R. Matthews, MD – Past Investigator
Jared R. Brosch, MD
Scott Herring, RN, CCRC

Yale University School of Medicine:
Christopher H. van Dyck, MD
Adam P. Mecca, MD, PhD
Adam P. Mecca, MD, PhD
Susan P. Good, APRN
Martha G. MacAvoy, PhD
Richard E. Carson, PhD
Pradeep Varma, MD

McGill Univ., Montreal-Jewish General Hospital:
Howard Chertkow, MD
Susan Vaitekunis, MD
Chris Hosein, MEd

Sunnybrook Health Sciences, Ontario:
Sandra Black, MD, FRCPC
Bojana Stefanovic, PhD
Chris (Chinthaka) Heyn, BSC, PhD, MD, FRCPC

U.B.C. Clinic for AD & Related Disorders:
Ging-Yuek Robin Hsiung, MD, MHSc, FRCPC
Ellen Kim, BA
Benita Mudge, BS
Vesna Sossi, PhD
Howard Feldman, MD, FRCPC – Past Investigator
Michele Assaly, MA – Past Investigator

St. Joseph's Health Care:
Elizabeth Finger, MD
Stephen Pasternak, MD
Irina Rachinsky, MD
Andrew Kertesz, MD – Past Investigator
Dick Drost, MD – Past Investigator
John Rogers, MD – Past Investigator

Northwestern University:
Ian Grant, MD
Brittanie Muse, MSPH
Emily Rogalski, PhD
Jordan Robson
M.-Marsel Mesulam, MD – Past Investigator
Diana Kerwin, MD – Past Investigator
Chuang-Kuo Wu, MD, PhD – Past Investigator
Nancy Johnson, PhD – Past Investigator
Kristine Lipowski, MA– Past Investigator
Sandra Weintraub, PhD – Past Investigator
Borna Bonakdarpour, MD– Past Investigator

Nathan Kline Institute:
Nunzio Pomara, MD
Raymundo Hernando, MD
Antero Sarrael, MD

University of California, San Francisco:
Howard J. Rosen, MD
Bruce L. Miller, MD
David Perry, MD

Georgetown University Medical Center:
Raymond Scott Turner, MD, PhD
Kathleen Johnson, NP
Brigid Reynolds, NP
Kelly MCCann, BA
Jessica Poe, BS

Brigham and Women's Hospital:
Reisa A. Sperling, MD
Keith A. Johnson, MD
Gad A. Marshall, MD

Stanford University:
Jerome Yesavage, MD
Joy L. Taylor, PhD
Steven Chao, MD, PhD
Jaila Coleman, BA
Jessica D. White, BA – Past Investigator
Barton Lane, MD – Past Investigator
Allyson Rosen, PhD – Past Investigator
Jared Tinklenberg, MD – Past Investigator

Banner Sun Health Research Institute:
Christine M. Belden, PsyD
Alireza Atri, MD, PhD
Bryan M. Spann, DO, PhD
Kelly A. Clark
Edward Zamrini, MD – Past Investigator
Marwan Sabbagh, MD – Past Investigator

Boston University:
Ronald Killiany, PhD
Robert Stern, PhD
Jesse Mez, MD, MS
Neil Kowall, MD – Past Investigator
Andrew E. Budson, MD – Past Investigator

Howard University:
Thomas O. Obisesan, MD, MPH
Oyonumo E. Ntekim, MD, PhD
Saba Wolday, MSc
Javed I. Khan, MD
Evaristus Nwulia, MD
Sheeba Nadarajah, PhD

Case Western Reserve University:
Alan Lerner, MD
Paula Ogrocki, PhD
Curtis Tatsuoka, PhD
Parianne Fatica, BA, CCRC

University of California, Davis – Sacramento:
Evan Fletcher, PhD
Pauline Maillard, PhD
John Olichney, MD
Charles DeCarli, MD
Owen Carmichael, PhD – Past Investigator

Dent Neurologic Institute:
Vernice Bates, MD

Horacio Capote, MD
Michelle Rainka, PharmD, CCRP

Parkwood Institute:
Michael Borrie, MB ChB
T-Y Lee, PhD
Dr Rob Bartha, PhD

University of Wisconsin:
Sterling Johnson, PhD
Sanjay Asthana, MD
Cynthia M. Carlsson, MD, MS

Banner Alzheimer's Institute:
Allison Perrin, PhD
Anna Burke, PhD – Past Investigator

Ohio State University:
Douglas W. Scharre, MD
Maria Kataki, MD, PhD
Rawan Tarawneh, MD
Brendan Kelley, MD – Past Investigator

Albany Medical College:
David Hart, MD
Earl A. Zimmerman, MD
Dzintra Celmins, MD

University of Iowa College of Medicine
Delwyn D. Miller, PharmD, MD
Laura L. Boles Ponto, PhD
Karen Ekstam Smith, RN
Hristina Koleva, MD
Hyungsub Shim, MD
Ki Won Nam, MD – Past Investigator
Susan K. Schultz, MD – Past Investigator

Wake Forest University Health Sciences:
Jeff D. Williamson, MD, MHS
Suzanne Craft, PhD
Jo Cleveland, MD
Mia Yang, MD– Past Investigator
Kaycee M. Sink, MD, MAS – Past Investigator

Rhode Island Hospital:
Brian R. Ott, MD
Jonathan Drake, MD
Geoffrey Tremont, PhD
Lori A. Daiello, Pharm.D, ScM
Jonathan D. Drake, MD

Cleveland Clinic Lou Ruvo Center for Brain Health:
Marwan Sabbagh, MD
Aaron Ritter, MD
Charles Bernick, MD, MPH – Past Investigator
Donna Munic, PhD – Past Investigator
Akiva Mintz, MD, PhD – Past Investigator

Roper St. Francis Healthcare:
Abigail O'Connelll, MS, APRN, FNP-C
Jacobo Mintzer, MD, MBA
Arthur Wiliams, BS

Houston Methodist Neurological Institute:
Joseph Masdeu, PhD

Barrow Neurological Institute:
Jiong Shi, MD, PhD
Angelica Garcia, BS
Marwan Sabbagh – Past Investigator

Vanderbilt University Medical Center:
Paul Newhouse, PhD

Long Beach VA Neuropsychiatric Research Program:
Steven Potkin, PhD

Butler Hospital Memory and Aging Program:
Stephen Salloway, MD, MS
Paul Malloy, PhD
Stephen Correia, PhD

Neurological Care of CNY:
Smita Kittur, MD – Past Investigator

Hartford Hospital, Olin Neuropsychiatry Research Center:
Godfrey D. Pearlson, MD – Past Investigator
Karen Blank, MD – Past Investigator
Karen Anderson, RN – Past Investigator

Dartmouth-Hitchcock Medical Center:
Laura A. Flashman, PhD – Past Investigator
Marc Seltzer, MD – Past Investigator
Mary L. Hynes, RN, MPH – Past Investigator
Robert B. Santulli, MD – Past Investigator

Cornell University
Norman Relkin, MD, PhD – Past Investigator
Gloria Chiang, MD – Past Investigator
Athena Lee, PhD

Michael Lin, MD – Past Investigator
Lisa Ravdin, PhD – Past Investigator

II. DOD ADNI

Part A: Leadership and Infrastructure

Principal Investigator

Michael W. Weiner, MD	University of California, San Francisco

ATRI PI and Director of Coordinating Center Clinical Core

Paul Aisen, MD	University of Southern California
Co Director Clinical Core Ron Petersen	Mayo Clinic

Executive Committee

Michael W. Weiner, MD	University of California, San Francisco
Paul Aisen, MD	University of Southern California
Ronald Petersen, MD, PhD	Mayo Clinic, Rochester
Robert C. Green, MD, MPH	Brigham and Women's Hospital/ Harvard Medical School
Danielle Harvey, PhD	University of California, Davis
Clifford R. Jack, Jr., MD	Mayo Clinic, Rochester
William Jagust, MD	University of California, Berkeley
John C. Morris, MD	Washington University St. Louis
Andrew J. Saykin, PsyD	Indiana University
Leslie M. Shaw, PhD	Perelman School of Medicine, University of Pennsylvania
Arthur W. Toga, PhD	University of Southern California
John Q. Trojanowski, MD, PhD	Perelman School of Medicine, University of Pennsylvania

Psychological Evaluation/PTSD Core

Thomas Neylan, MD	University of California, San Francisco

Traumatic Brain Injury/TBI Core

Jordan Grafman, PhD	Rehabilitation Institute of Chicago, Feinberg School of Medicine, Northwestern University

Data and Publication Committee (DPC)

Robert C. Green, MD, MPH	BWH/HMS (Chair)

Resource Allocation Review Committee

Tom Montine, MD, PhD	University of Washington (Chair)

Clinical Core Leaders and Key Personnel

Michael W. Weiner MD	Core PI
Ronald Petersen, MD, PhD	Mayo Clinic, Rochester (Core PI)
Paul Aisen, MD	University of Southern California (Core PI)
Gustavo Jimenez, MBS	University of Southern California
Michael Donohue, PhD	University of Southern California
Devon Gessert, BS	University of Southern California
Jennifer Salazar, MBS	University of Southern California
Caileigh Zimmerman, MS	University of Southern California
Sarah Walter, MSc	University of Southern California
Olusegun Adegoke, MSc	University of Southern California
Payam Mahboubi, MPH	University of Southern California

	Lindsey Hergesheimer, BS
University of Southern California	
Sarah Danowski, MA	University of Southern California
Godfrey Coker, MBA, MPH	University of Southern California
Taylor Clanton, MPH	University of Southern California
Jeremy Pizzola, BA	University of Southern California
Elizabeth Shaffer, BS	University of Southern California
Catherine Nguyen-Barrera, MS	University of Southern California

San Francisco Veterans Affairs Medical Center

Thomas Neylan, MD	University of California, San Francisco
Jacqueline Hayes	University of California, San Francisco
Shannon Finley	University of California, San Francisco

Biostatistics Core Leaders and Key Personnel

Danielle Harvey, PhD	University of California, Davis (Core PI)
Michael Donohue, PhD	University of California, San Diego

MRI Core Leaders and Key Personnel

Clifford R. Jack, Jr., MD	Mayo Clinic, Rochester (Core PI)
Matthew Bernstein, PhD	Mayo Clinic, Rochester
Bret Borowski, RT	Mayo Clinic
Jeff Gunter, PhD	Mayo Clinic
Matt Senjem, MS	Mayo Clinic
Kejal Kantarci	Mayo Clinic
Chad Ward	Mayo Clinic
Duygu Tosun-Turgut, PhD	University of California, San Francisco
Stephanie Rossi Chen, BA	NCIRE / The Vererans Health Research Institute

PET Core Leaders and Key Personnel

Susan Landau, PhD	University of California, Berkeley Core PI
Robert A. Koeppe, PhD	University of Michigan
Norm Foster, MD	University of Utah
Eric M. Reiman, MD	Banner Alzheimer's Institute
Kewei Chen, PhD	Banner Alzheimer's Institute

Neuropathology Core Leaders

John C. Morris, MD	Washington University St. Louis
Richard J. Perrin, MD, PhD	Washington University St. Louis
Erin Franklin, MS	Washington University St. Louis

Biomarkers Core Leaders and Key Personnel

Leslie M. Shaw, PhD	Perelman School of Medicine, University of Pennsylvania
John Q. Trojanowski, MD, PhD	Perelman School of Medicine, University of Pennsylvania
Magdalena Korecka, PhD	Perelman School of Medicine, University of Pennsylvania
Michal Figurski, PhD	Perelman School of Medicine, University of Pennsylvania

Informatics Core Leaders and Key Personnel

Arthur W. Toga, PhD	University of Southern California (Core PI)
Karen Crawford	University of Southern California

Scott Neu, PhD University of Southern California

Genetics Core Leaders and Key Personnel

Andrew J. Saykin, PsyD University
Tatiana M. Foroud, PhD Indiana University
Steven Potkin, MD UC UC Irvine
Li Shen, PhD Indiana University

Kelley Faber, MS, CCRC Indiana University
Sungeun Kim, PhD Indiana University
Kwangsik Nho, PhD Indiana University
Kristi Wilmes, MS, CCRP NCRAD

Part B: Investigators By Site

University of Southern California:
Lon S. Schneider, MD
Sonia Pawluczyk, MD
Mauricio Becerra, MD
Liberty Teodoro, RN
Karen Dagerman, MS
Bryan M. Spann, DO, PhD – Past Investigator

University of California, San Diego:
James Brewer, MD, PhD
Helen Vanderswag, RN
Adam Fleisher, MD – Past Investigator

Columbia University Medical Center:
Yaakov Stern, PhD
Lawrence S. Honig, MD, PhD
Akiva Mintz, MD, PhD

Rush University Medical Center:
Raj C. Shah, MD
Ajay Sood, MD, PhD
Kimberly S. Blanchard, DNP, APRN, NP-C
Debra Fleischman, PhD – Past Investigator
Konstantinos Arfanakis, PhD – Past Investigator

Wien Center:
Dr. Ranjan Duara MD PI
Dr. Daniel Varon MD Co-PI
Maria T Greig HP Coordinator

Duke University Medical Center:
P. Murali Doraiswamy, MBBS, FRCP
Jeffrey R. Petrella, MD
Olga James, MD– Past Investigator
Salvador Borges-Neto, MD
Terence Z. Wong, MD

University of Rochester Medical Center:
Anton P. Porsteinsson, MD
Bonnie Goldstein, MS, NP
Kimberly S. Martin, RN

University of California, Irvine:
Gaby Thai, MD
Aimee Pierce, MD
Christopher Reist, MD
Beatriz Yanez, RN
Elizabeth Sosa, PhD
Megan Witbracht, PhD

Premiere Research Inst (Palm Beach Neurology):
Carl Sadowsky, MD
Walter Martinez, MD
Teresa Villena, MD

University of California, San Francisco:
Howard Rosen, MD
David Perry

Georgetown University Medical Center:
Raymond Scott Turner, MD, PhD
Kathleen Johnson, NP
Brigid Reynolds, NP
Kelly MCCann, BA
Jessica Poe, BS

Brigham and Women's Hospital:
Reisa A. Sperling, MD
Keith A. Johnson, MD

Gad Marshall, MD

Banner Sun Health Research Institute:
Christine M. Belden, PsyD
Alireza Atri, MD, PhD
Bryan M. Spann, DO, PhD
Kelly A. Clark
Edward Zamrini, MD – Past Investigator
Marwan Sabbagh, MD – Past Investigator

Howard University:
Thomas O. Obisesan, MD, MPH
Oyonumo E. Ntekim, MD, PhD
Saba Wolday, MSc
Evaristus Nwulia, MD
Sheeba Nadarajah, PhD, RN

University of Wisconsin:
Sterling Johnson, PhD
Sanjay Asthana, MD
Cynthia M. Carlsson, MD, MS

University of Washington:
Elaine R. Peskind, MD
Eric C. Petrie, MD, MS
Gail Li, MD, PhD

Stanford University:
Jerome Yesavage, MD
Joy L. Taylor, PhD
Steven Chao, MD, PhD
Jaila Coleman, BA
Jessica D. White, BA – Past Investigator
Barton Lane, MD – Past Investigator
Allyson Rosen, PhD – Past Investigator
Jared Tinklenberg, MD – Past Investigator

Cornell University:
Michael Lin, PhD
Gloria Chiang, MD
Lisa Ravdin, PhD
Norman Relkin, MD, PhD – Past Investigator

Roper St. Francis Healthcare:
Abigail O'Connelll, MS, APRN, FNP-C
Jacobo Mintzer, MD, MBA
Arthur Wiliams, BS

III. ADNI Depression

Part A: Leadership and Infrastructure

Principal Investigator

Scott Mackin, PhD	University of California, San Francisco

ATRI Coordinating Center Clinical Core

Paul Aisen, MD	University of Southern California
Rema Raman, PhD	University of Southern California
Gustavo Jimenez-Maggiora, MBS	University of Southern California
Michael Donohue, PhD	University of Southern California
Devon Gessert, BS	University of Southern California
Jennifer Salazar, MBS	University of Southern California
Caileigh Zimmerman, MS	University of Southern California
Sarah Walter, MSc	University of Southern California
Olusegun Adegoke, MSc	University of Southern California
Payam Mahboubi, MPH	University of Southern California

Executive Committee

Scott Mackin, PhD	University of California, San Francisco
Michael W. Weiner, MD	University of California, San Francisco
Paul Aisen, MD	University of Southern California
Rema Raman, PhD	University of Southern California
Clifford R. Jack, Jr., MD	Mayo Clinic, Rochester
Susan Landau, PhD	University of California, Berkeley
Andrew J. Saykin, PsyD	Indiana University
Arthur W. Toga, PhD	University of Southern California
Charles DeCarli, MD	University of California, Davis
Robert A. Koeppe, PhD	University of Michigan

Data and Publication Committee (DPC)

Robert C. Green, MD, MPH	BWH/HMS (Chair)
Erin Drake, MA	BWM/HMS (Director)

Clinical Core Leaders

Michael W. Weiner MD	Core PI
Paul Aisen, MD	University of Southern California
Rema Raman, PhD	University of Southern California
Mike Donohue, PhD	University of Southern California

Psychiatry Site Leaders and Key Personnel

Scott Mackin, PhD	University of California, San Francisco
Craig Nelson, MD	University of California, San Francisco
David Bickford, BA	University of California, San Francisco
Meryl Butters, PhD	University of Pittsburgh
Michelle Zmuda, MA	University of Pittsburgh

MRI Core Leaders and Key Personnel

Clifford R. Jack, Jr., MD Mayo Clinic, Rochester (Core PI)
Matthew Bernstein, PhD Mayo Clinic, Rochester
Bret Borowski, RT Mayo Clinic, Rochester
Jeff Gunter, PhD Mayo Clinic, Rochester
Matt Senjem, MS Mayo Clinic, Rochester
Kejal Kantarci, MD Mayo Clinic, Rochester
Chad Ward, BA Mayo Clinic, Rochester
Denise Reyes, BS Mayo Clinic, Rochester

PET Core Leaders and Key Personnel
Robert A. Koeppe, PhD University of Michigan
Susan Landau, PhD University of California, Berkeley

Informatics Core Leaders and Key Personnel
Arthur W. Toga, PhD University of Southern California (Core PI)
Karen Crawford University of Southern California
Scott Neu, PhD University of Southern California

Genetics Core Leaders and Key Personnel
Andrew J. Saykin, PsyD Indiana University
Tatiana M. Foroud, PhD Indiana University
Kelley M. Faber, MS, CCRC Indiana University
Kwangsik Nho, PhD Indiana University
Kelly N. Nudelman Indiana University

Part B: Investigators By Site

University of California, San Francisco:
Scott Mackin, PhD
Howard Rosen, MD
Craig Nelson, MD
David Bickford, BA
Yiu Ho Au, BA
Kelly Scherer, BS
Daniel Catalinotto, BA
Samuel Stark, BA
Elise Ong, BA
Dariella Fernandez, BA

University of Pittsburgh:
Meryl Butters, PhD
Michelle Zmuda, BS
Oscar L. Lopez, MD
MaryAnn Oakley, MA
Donna M. Simpson, CRNP, MPH
