# Supplementary material for: Gliovascular transcriptional perturbations in Alzheimer’s disease reveal molecular mechanisms of blood brain barrier dysfunction
Source: Nat Commun. 2024 Jun 20;15:4758. doi: 10.1038/s41467-024-48926-6 (PMC11190273; doi:10.1038/s41467-024-48926-6)
Supplement: Supplementary file 3 — Description of Additional Supplementary Files [file 41467_2024_48926_MOESM3_ESM.pdf]

Description of Additional Supplementary Files

File Name: Supplementary Data 1 – 45
Description:

Supplementary Data 1. Postmortem snRNAseq donor information table. Information for each of the 24 brain donors' in the postmortem snRNAseq study is listed. Patient demographics include sex, age of death, *APOE* genotype and race. Postmortem pathology information include brain weight at death, Braak stage, Thal phase, presence of TDP43 aggregates, α-synuclein and vascular dementia. For AD patients, duration of dementia is also included. RNA integrity number (RIN), that was utilized to check the quality of brain tissue samples is included. Raw snRNAseq measures such as total number of reads, estimated number of cells, mean reads per cells, and median genes per cell for each donor is also listed.

Supplementary Data 2. Cell type assignment of each cluster. Number of post-QC nuclei per cluster for diagnosis groups within all 35 clusters in postmortem snRNAseq study were listed. Annotation of each cluster by brain cell type is included (OL: oligodendrocytes; neu: neurons; ast: astrocyte; OPCs: oligodendrocyte precursor cells; mic: microglia; ex: excitatory neurons; in: inhibitory neurons).

Supplementary Data 3. Number of cells in each cluster per donor. Upper table depicts the number of post-QC nuclei per cluster for each donor group for all 35 clusters in the postmortem snRNAseq study. Lower table depicts the representation of each donor from this study in the astrocyte and pericyte clusters integrated with external datasets shown in Supplementary Data 31.

Supplementary Data 4. Cluster cell proportions in AD and control brains. Number of post-QC nuclei per cluster for diagnosis groups within all 35 clusters in postmortem snRNAseq study were listed. In order to test whether AD diagnosis has impact on the clusters, the number of

cells in an individual for that cluster was divided by the total number of cells in all clusters for that individual. The resulting ratio was used to test for association with diagnosis using a Wilcoxon rank sum test for binary variables. All statistical tests were two-sided. P-values <0.05 were considered statistically significant. Any significant results are highlighted in yellow.

Supplementary Data 5. Cluster cell proportions by demographic factors. Number of post-QC nuclei per cluster for demographic factors such as *APOE* genotype, sex, and age within all 35 clusters in postmortem snRNAseq study were listed. For each cluster, the number of cells in an individual for that cluster, was divided by the total number of cells in all clusters for that individual. The resulting ratio gives the cell distribution that was used to test for association with each demographic factor using a Wilcoxon rank sum test for binary variables (male vs. female, *APOEε4* positive vs. negative) or Spearman's test of correlation for quantitative/semi-quantitative variables (age at death). All statistical tests were two-sided. P-values <0.05 were considered statistically significant. Any significant results are highlighted in yellow.

Supplementary Data 6. Cluster cell proportions by pathologic variables. Number of post-QC nuclei per cluster for neuropathology scores such as Braak stage, Thal phase, and presence of TDP43 aggregates within all 35 clusters in postmortem snRNAseq study were listed. For each cluster, the number of cells in an individual for that cluster, was divided by the total number of cells in all clusters for that individual. The resulting ratio gives the cell distribution that was used to test for association with characteristics using a Wilcoxon rank sum test for binary variables (TDP-43 positive vs. negative) or Spearman's test of correlation for quantitative/semi-quantitative variables (Thal phase, and Braak stage). All statistical tests were two-sided. P-values <0.05 were considered statistically significant. Any significant results are highlighted in yellow.

Supplementary Data 7. Percent intermediate or ambiguous cells identified by random forest classification. Analyses were done for each of the vascular or astrocyte clusters against all other clusters. For each pair of clusters, cells from the two clusters were divided into four groups. For

each group, cells in the other three were used as training data, and the cells were classified as one of the two clusters. Therefore, each cell was classified 100 times, and this classification procedure was repeated. If a cell was misclassified > 25 times, it was considered as "ambiguous" or "intermediate". The percentage of intermediate cells was calculated to be 100* (num of intermediate cells)/(num.cell.clusterA + num.cell.clusterB).

Supplementary Data 8. Signature genes for the three brain vascular cell clusters. Signature genes were defined as those that were detected in at least 50% cells in one cluster, had average expression >= 2.0X that of either of the other two clusters with Bonferroni p-value < 0.05. This resulted in 102, 174 and 80 signature genes for cl.25, cl.26 and cl.30 respectively.

Supplementary Data 9. GO term enrichment for vascular cluster 25 (pericyte) signature genes. MSigDB v7.0 was used for Gene Ontology enrichment analyses. The enrichment of selected genes in MSigDB C5 category (i.e., gene ontology or GO) was performed using R enRichment package. Fifteen GO terms were enriched for this cluster with FDR<0.05.

Supplementary Data 10. GO term enrichment for vascular cluster 26 (endothelia) signature genes. MSigDB v7.0 was used for Gene Ontology enrichment analyses. The enrichment of selected genes in MSigDB C5 category (i.e., gene ontology or GO) was performed using R enRichment package. 57 GO terms were enriched for this cluster with FDR<0.05.

Supplementary Data 11. GO term enrichment for vascular cluster 30 (perivascular fibroblasts) signature genes. MSigDB v7.0 was used for Gene Ontology enrichment analyses. The enrichment of selected genes in MSigDB C5 category (i.e., gene ontology or GO) was performed using R enRichment package. 17 GO terms were enriched for this cluster with FDR<0.05.

Supplementary Data 12. Signature genes for the three brain astrocyte cell clusters. Signature genes were defined as those that were detected in at least 50% cells in one cluster, had average

expression >= 2.0X that of either of the other two clusters with Bonferroni p-value < 0.05. This resulted in 20, 12 and 274 signature genes for cl.8, cl.11 and cl.31 respectively.

Supplementary Data 13 GO term enrichment for astrocyte cluster 31 signature genes. Cluster 31 showed a higher number of signature genes compared to the other two astrocyte clusters, which in turn, enabled Gene Ontology enrichment analyses. The enrichment of selected genes in MSigDB C5 category (i.e., gene ontology or GO) was performed using R enRichment package. 69 GO terms were enriched for this cluster with FDR<0.05.

Supplementary Data 14. Genes with significant differential expression in AD vs. control brain vascular clusters. Genes with significant differential expression in AD vs. control brain vascular clusters are shown. MAST R package was used to detect differentially expressed genes (DEGs). Imposing q value < 0.05, absolute log (fold change) > 0.1 and detection of gene expression in >= 20% cells, 220 (156 up, 64 down), 44 (34 up, 10 down), and 14 (8 up and 6 down) DEGs were identified in cl.25, cl.26 and cl.30 respectively.

Supplementary Data 15. GO term enrichment for vascular cluster 25 (pericyte) genes upregulated in AD. MSigDB v7.0 was used for Gene Ontology enrichment analyses in 156 upregulated genes in cluster 25. The enrichment of selected genes in MSigDB C5 category (i.e., gene ontology or GO) was performed using R enRichment package. 8 GO terms that include hormone receptor signaling were enriched for this cluster with FDR<0.05.

Supplementary Data 16. GO term enrichment for vascular cluster 25 (pericyte) genes downregulated in AD. MSigDB v7.0 was used for Gene Ontology enrichment analyses in 64 downregulated genes in cluster 25. The enrichment of selected genes in MSigDB C5 category (i.e., gene ontology or GO) was performed using R enRichment package. None of the GO terms reached significance with FDR<0.05.

Supplementary Data 17. GO term enrichment for vascular cluster 26 (endothelia) genes upregulated in AD. MSigDB v7.0 was used for Gene Ontology enrichment analyses in 34 upregulated genes in cluster 26. The enrichment of selected genes in MSigDB C5 category (i.e., gene ontology or GO) was performed using R enRichment package. None of the GO terms reached significance with FDR<0.05.

Supplementary Data 18. Genes with significant differential expression in AD vs. control brain astrocyte clusters. Genes with significant differential expression in AD vs. control brain astrocytic clusters. MAST R package was used to detect differentially expressed genes (DEGs). Imposing q value < 0.05, absolute log (fold change) > 0.1 and detection of gene expression in >= 20% cells, 696 (312 up, 384 down), 822 (573 up, 249 down), 328 (139 up, 189 down) DEGs were identified in cl.8, cl.11 and cl.31 respectively.

Supplementary Data 19. GO term enrichment for astrocytic cluster 8 genes upregulated in AD. MSigDB v7.0 was used for Gene Ontology enrichment analyses in 312 upregulated genes in cluster 8. The enrichment of selected genes in MSigDB C5 category (i.e., gene ontology or GO) was performed using R enRichment package. 6 GO terms were enriched for this cluster with FDR<0.05.

Supplementary Data 20. GO term enrichment for astrocytic cluster 11 genes upregulated in AD. MSigDB v7.0 was used for Gene Ontology enrichment analyses in 573 upregulated genes in cluster 11. The enrichment of selected genes in MSigDB C5 category (i.e., gene ontology or GO) was performed using R enRichment package. 108 GO terms were enriched for this cluster with FDR<0.05.

Supplementary Data 21. GO term enrichment for astrocytic cluster 31 genes upregulated in AD. MSigDB v7.0 was used for Gene Ontology enrichment analyses in 139 upregulated genes in cluster 31. The enrichment of selected genes in MSigDB C5 category (i.e., gene ontology or GO)

was performed using R enRichment package. 35 GO terms were enriched for this cluster with FDR<0.05.

Supplementary Data 22. GO term enrichment for astrocytic cluster 8 genes downregulated in AD. MSigDB v7.0 was used for Gene Ontology enrichment analyses in 384 downregulated genes in cluster 8. The enrichment of selected genes in MSigDB C5 category (i.e., gene ontology or GO) was performed using R enRichment package. 62 GO terms were enriched for this cluster with FDR<0.05.

Supplementary Data 23. GO term enrichment for astrocytic cluster 11 genes downregulated in AD. MSigDB v7.0 was used for Gene Ontology enrichment analyses in 249 downregulated genes in cluster 11. The enrichment of selected genes in MSigDB C5 category (i.e., gene ontology or GO) was performed using R enRichment package. 14 GO terms were enriched for this cluster with FDR<0.05.

Supplementary Data 24. GO term enrichment for astrocytic cluster 31 genes downregulated in AD. MSigDB v7.0 was used for Gene Ontology enrichment analyses in 189 downregulated genes in cluster 31. The enrichment of selected genes in MSigDB C5 category (i.e., gene ontology or GO) was performed using R enRichment package. None of the GO terms reached significance with FDR<0.05.

Supplementary Data 25. NicheNet astrocyte ligand-vascular target interactions. NicheNet astrocyte ligand-vascular target interactions are shown in the columns. Column header descriptions: ligand-Ast.cluster-number: Ligands from a specific astrocytic cluster denoted by its number, for which there is one or more qualifying target gene in corresponding vascular cluster. target-Vasc.cluster-number: Target genes in vasculer cluster for the corresponding astrocytic ligand. weight: The regulation strength of ligand for target according to NicheNet prior model.

Supplementary Data 26. Vascular prioritized target genes. 27 vascular target genes were identified through NicheNet analysis. Six vascular target genes that are selected for validation studies are highlighted in yellow. (Column header descriptions: cell type: cell type assigned to gene according to BRETIGEA; biological function: Known function of gene/protein based on literature; target_vascular cluster-number: the cluster in which the gene resides and is identified as a target (1=yes, 0=no); vascular cluster-number.coef: coefficient for differential gene expression analysis between AD and control brain tissue nuclei in that vascular cluster; vascular cluster-number.qValue: q value of differential gene expression analysis between AD and control brain tissue nuclei in that vascular cluster; Sum.vascular-cluster-number.reg: sum of regulation strength (according to NicheNet prior model) of this gene by ligands in all 3 astrocyte clusters; Num.vascular-cluster-number.reg: number of of known regulations (according to NicheNet prior model) between this gene and ligands in 3 astrocyte clusters.

Supplementary Data 27. Expression of six prioritized vascular target genes in isolated nuclei. Expression of six prioritized vascular target genes (*AHNAK, ANGPT2, ECE1, SMAD3, STAT3*, and *TSC22D3*) were validated via qPCR from isolated nuclei of 20 out of 24 donors of postmortem snRNAseq cohort, where extra brain tissue was available. Negative delta-CT values from qPCR measurements of six prioritized vascular target genes are shown.

Supplementary Data 28. Percentage of VEGFA positivity in AGT positive nuclei. Nuclei that were isolated from human temporal cortex of 9 AD and 9 control donors were co-stained with *VEGFA* and *AGT* RNAscope probe pairs. Images were captured via Operetta CLS high content imager (Perkin Elmer) and processed through custom RNAscope pipeline on Cell Profiler to analyze a total number of 50,946 DAPI$^+$ nuclei (AD: 25,055, Control: 25,891) for astrocytic staining. Out of these, 5,370 nuclei (AD: 2,769 Control: 2,601) were annotated as AGT$^+$ from 16 images per donor. VEGFA$^+$ staining in AGT$^+$ nuclei ranged from 10 % to 68 % (Median = 40.89).

Supplementary Data 29. Percentage of SMAD3 positivity in LEF1 positive nuclei. Nuclei that were isolated from human temporal cortex of 9 AD and 9 control donors were co-stained with

SMAD3 and *LEF1* RNAscope probe pairs. Images were captured via Operetta CLS high content imager (Perkin Elmer) and processed through custom RNAscope pipeline on Cell Profiler to analyze a total number of 62,072 nuclei (AD: 32,442, Control: 29,630) for vascular staining. Out of these, 1,486 nuclei (AD: 755 Control: 731 were annotated as LEF1$^+$ from 16 images per donor. SMAD3$^+$ staining in AGT$^+$ nuclei ranged from 14% to 62% (Median = 41.78).

Supplementary Data 30. pSMAD3 immunoreactivity in postmortem brain from AD and control donors. pSMAD3 immunoreactivity was measured in 10 AD and 10 Control donors. AD pericytes showed significantly increased (p<0.01) pSMAD3 reactivity compared to control donors (EC: Entorhinal cortex). Three blood vessels per case in each donor (specifically, blood vessels located in the entorhinal cortex and the adjacent white matter) were manually annotated using the Aperio ImageScope software (ECvessel: Blood vessels in entorhinal cortex). AD pericytes showed significantly increased (p<0.01) pSMAD3 reactivity compared to control donors.

Supplementary Data 31. Summary of external snRNAseq datasets used in this study. *SMAD3* and *VEGFA* expression were analyzed in external datasets in AD pericytes and astrocytes, respectively. Analysis included 4,730 pericyte and 150,664 astrocyte nuclei from Mathys et al.[14], Mathys et al.[20], Grubman et al.[15], Yang et al.[22], Sun et al.[21], Zhang et al[23] and this study. The brain regions which were utilized for analysis are listed (EC: Entorhinal cortex, DLPFC: Dorsolateral prefrontal cortex, HC: Hippocampus, SFX: Superior frontal cortex, PFC: Prefrontal cortex, AG: Angular gyrus, MTX: Midtemporal cortex, TH: Thalamus).

Supplementary Data 32. Differential expression results of *SMAD3* in pericyte nuclei from integrated analysis of multiple studies. Within integrated pericyte dataset clusters, *SMAD3* differential expression is analyzed between AD and control donors for the integrated pericyte clusters for each study and all studies combined. Negative binomial generalized linear mixed effects model was used for differential expression analysis. *SMAD3* is significantly upregulated in the largest integrated pericyte cluster (p<0.05).

Supplementary Data 33. Differential expression results of *VEGFA* in astrocyte nuclei from integrated analysis of multiple studies. Within integrated astrocyte dataset clusters, *VEGFA* differential expression is analyzed between AD and control donors for the integrated astrocyte clusters for each study and all studies combined. Negative binomial generalized linear mixed effects model was used for differential expression analysis. *VEGFA* is significantly downregulated in the two largest astrocyte clusters and all clusters combined ($p<0.05$).

Supplementary Data 34. Signature genes for Pericyte Cluster0 and Astrocyte Cluster0 in integrated single nuclei dataset. Signature genes for Pericyte Cluster0 and Astrocyte Cluster0 in the integrated single nuclei dataset are shown. Signature genes are those that were detected in at least 50% cells in one cluster, had average expression >= 2.0X that of either of the other clusters with Bonferroni p-value < 0.05.

Supplementary Data 35. Association of infarcts with 6 *SMAD3* variants. Presence or absence of infarcts on MRI were detected from T2-weighted images. 1,508 MCSA and 1,080 ADNI participants were analyzed for associations of infarcts with *SMAD3* locus variants. 6 intronic *SMAD3* variants (rs71400360, rs12904527, rs12909923, rs71400361, rs35779650, rs28564777) had nominally significant associations ($p<0.05$) with lower brain infarcts in MCSA, and meta-analyzed cohorts with lower estimates in ADNI that did not reach statistical significance.

Supplementary Data 36. Association of *SMAD3* variants blood *SMAD3* gene expression. Association of the 6 *SMAD3* variants with blood *SMAD3* gene expression in the primary model adjusting for *APOE* and secondary model without *APOE* adjustment. Gene expression in MCSA was measured with RNAseq and that in ADNI using 5 expression arrays probes for *SMAD3*. Results are shown for MCSA, ADNI and their meta-analysis for these eQTL analyses.

Supplementary Data 37. *SMAD3* expression in iPSC derived pericyte experiments. Four patient derived iPSCs were differentiated into pericytes and used to assess *SMAD3* expression changes after treatment with VEGF, VEGFR2 inhibitor and aggregated Aβ. DeltaCt values for *SMAD3* are shown for each treatment condition, along with the experimental condition identifiers.

Supplementary Data 38. Zebrafish scRNAseq clusters. ScRNAseq data obtained from the adult zebrafish telencephalon of double reporter transgenic zebrafish line (fli1a:GFP, her4:DsRed), treated with Aβ vs. vehicle is shown for the 34 cell type clusters.

Supplementary Data 39. Zebrafish scRNAseq cluster cell type numbers in Abeta treated vs vehicle treated conditions. Total number of brain cell types were listed. The highest number of cells that were obtained was from astrocytes (Aβ42: 3,249, PBS: 5,280).

Supplementary Data 40. iPSC Donor information. The demographics of iPSC donors are listed. Previously fully characterized, 2 AD and 2 control donors with *APOE* e4/e4 genotype were selected for iPSCs experiments**.**

Supplementary Data 41. Mycoplasma contamination measurements in iPSCs. Mycoplasma contamination in iPSCs were checked via MycoAlert® PLUS Mycoplasma Detection Kit. All of our readings were below 1, which is an indication of lack of mycoplasma contamination. This is also confirmed by the very high reading from the positive control sample.

Supplementary Data 42. Testing for over-representations of nuclei from donor(s) in the integrated astrocyte dataset. To test if there is over-representation of nuclei from certain donor(s) in each integrated astrocyte cluster, we performed one-sided Fisher's Exact Test (FET). We applied a corrected FET p-value < 0.05 and enrichment fold change (EFC) > 4 to identify donor enrichment per cluster where enriched=TRUE and not enriched=FALSE.

Supplementary Data 43. Testing for over-representations of nuclei from donor(s) in integrated pericyte dataset. To test if there is over-representation of nuclei from certain donor(s) in each integrated pericyte cluster, we performed one-sided Fisher's Exact Test (FET). We applied a corrected FET p-value < 0.05 and enrichment fold change (EFC) > 4 to identify donor enrichment per cluster where enriched=TRUE and not enriched=FALSE.

Supplementary Data 44. Statistical models used in the snRNAseq analysis. Both the analytic models and the # of donors used in the various analyses are shown.

Supplementary Data 45. *VEGFA* expression in an astrocyte-enriched snRNAseq study. Differential expression results of *VEGFA* expression between AD and control donors from Sadick et al[102]. is shown.
