# Supplementary material for: Gliovascular transcriptional perturbations in Alzheimer’s disease reveal molecular mechanisms of blood brain barrier dysfunction
Source: Nat Commun. 2024 Jun 20;15:4758. doi: 10.1038/s41467-024-48926-6 (PMC11190273; doi:10.1038/s41467-024-48926-6)
Supplement: Supplementary file 5 — Reporting Summary [file 41467_2024_48926_MOESM5_ESM.pdf]

nature portfolio

Corresponding author(s): Nilufer Ertekin-Taner

Last updated by author(s): Apr 8, 2024

Reporting Summary

Nature Portfolio wishes to improve the reproducibility of the work that we publish. This form provides structure for consistency and transparency in reporting. For further information on Nature Portfolio policies, see our Editorial Policies and the Editorial Policy Checklist.

Statistics

For all statistical analyses, confirm that the following items are present in the figure legend, table legend, main text, or Methods section.

n/a	Confirmed	
☐	☒	The exact sample size (*n*) for each experimental group/condition, given as a discrete number and unit of measurement
☐	☒	A statement on whether measurements were taken from distinct samples or whether the same sample was measured repeatedly
☐	☒	The statistical test(s) used AND whether they are one- or two-sided<br>*Only common tests should be described solely by name; describe more complex techniques in the Methods section.*
☐	☒	A description of all covariates tested
☐	☒	A description of any assumptions or corrections, such as tests of normality and adjustment for multiple comparisons
☐	☒	A full description of the statistical parameters including central tendency (e.g. means) or other basic estimates (e.g. regression coefficient) AND variation (e.g. standard deviation) or associated estimates of uncertainty (e.g. confidence intervals)
☐	☒	For null hypothesis testing, the test statistic (e.g. *F*, *t*, *r*) with confidence intervals, effect sizes, degrees of freedom and *P* value noted<br>*Give P values as exact values whenever suitable.*
☒	☐	For Bayesian analysis, information on the choice of priors and Markov chain Monte Carlo settings
☒	☐	For hierarchical and complex designs, identification of the appropriate level for tests and full reporting of outcomes
☐	☒	Estimates of effect sizes (e.g. Cohen's *d*, Pearson's *r*), indicating how they were calculated

Our web collection on statistics for biologists contains articles on many of the points above.

Software and code

Policy information about availability of computer code

Data collection

The following software were used to collect data:

- Aperio ImageScope software (Leica Biosystems, ver 12.4.2.7000) was used to capture IHC images from postmortem brain samples.
- ZEN software (version blue edition, v3.2, Carl Zeiss, Jena, Germany) was used to capture IF images from postmortem brain samples.
- BD FACSDiva™ Software (BD Biosciences, V8) was used to set parameters to gate and sort nuclear fractions from frozen brain
- Harmony (Perkin-Elmer, v4.9) was used to acquire images for RNAscope assays
- Attune Cytometric Software (Thermo Fisher, v5.2.0) was used to set parameters to gate in flow cytometry experiments
- QuantStudio Real-Time PCR Software (Thermo Fisher, v1.3) was used to obtain RT-qPCR profile.
- BZ-X800 software: was used to capture ICC images from ICC images through Keyence microscope.

Data analysis

The following software/packages were used to analyze data:

- Cell Ranger v3.1.0, and v6.1.2 were used for human snRNAseq and zebrafish scRNAseq analysis
- R v4.2.2.10, v4.0.3 was used to visualize data and perform statistical analysis
- Python v3.10.11 was utilized to run srublet
- Seurat v5.0.1 and v4.3.0 was used for both human snRNAseq and zebrafish scRNAseq analysis, respectively
- srublet v0.2.1 was used to identify doublets in human snRNAseq dataset
- DoubletFinder v2.0 was used to identify doublets in zebrafish scRNAseq analysis
- MAST v1.24.1 package was used for differential gene expression analysis of human snRNAseq data
- glmmTMB v1.1.8 package was used for differential gene expression analysis of snRNAseq data

- nichenetr v1.1.1 package was used for NicheNet for ligand-target analysis between gliovascular clusters in human snRNAseq data
- PLINK v1.9 was used for genetic data analysis
- Cell Profiler v4.2.5 was to quantify RNAscope, and ICC analysis in human studies
- harmony v1.2.0 package was used for integration of scRNAseq and snRNAseq data
- ScType v1.0 package was used for cell type assignment in snRNAseq data
- MSigDB v7.0 was used to perform GO term enrichment analysis in human snRNAseq data
- anRichment v1.22 was used to perform GO term enrichment analysis in human snRNAseq data
- BRETIGEA v1.0.3 was used to annotate cell type marker genes
- MAP-RSeq v3.0 was used to QC Mayo MCSA blood RNAseq dataset
- FreeSurfer v5.1 was used to analyze MRI scans
- SurfStat math.mcgill.ca/keith/surfstat was used to perform multivariable analysis of MRI scans
- SPM12 (fil.ion.ucl.ac.uk/spm/) was used to perform statistical analysis of processed PET images

For manuscripts utilizing custom algorithms or software that are central to the research but not yet described in published literature, software must be made available to editors and reviewers. We strongly encourage code deposition in a community repository (e.g. GitHub). See the Nature Portfolio guidelines for submitting code & software for further information.

Data

Policy information about availability of data

All manuscripts must include a data availability statement. This statement should provide the following information, where applicable:

- Accession codes, unique identifiers, or web links for publicly available datasets
- A description of any restrictions on data availability
- For clinical datasets or third party data, please ensure that the statement adheres to our policy

All generated human snRNAseq data in this manuscript is available via the AD Knowledge Portal (https://adknowledgeportal.synapse.org). The AD Knowledge Portal is a platform for accessing data, analyses and tools generated by the Accelerating Medicines Partnership (AMP AD) Target Discovery Program and other National Institute on Aging (NIA)-supported programs to enable open-science practices and accelerate translational learning. Data is available for general research use according to the following requirements for data access and data attribution (https://adknowledgeportal.synapse.org/DataAccess/Instructions). An overview of all the data generated and used in this study can be found on the manuscript landing page (https://www.synapse.org/#!Synapse:syn52669545/datasets/). The single-nucleus RNAseq data generated in this study have been deposited in the AD Knowledge Portal under the Mayo snRNAseq study (accession ID: syn31511712). The zebrafish gliovascular single cell transcriptomics dataset can be accessed at NCBI's Gene Expression Omnibus (GEO) with the accession number GSE225721. The full complement of clinical and demographic data for the ADNI cohorts are hosted on the LONI data sharing platform and can be requested at http://adni.loni.usc.edu/data-samples/access-data/. The additional data generated in this study are provided in the Supplementary Information/Source Data file.

In addition this study utilizes following publicly available datasets.

- Grubman et al., 2019 Nature Neuroscience (adsn.ddnetbio.com)
- Mathys et al., 2019 Nature Neuroscience (synapse.org/#!Synapse:syn21261143)
- Yang et al., 2022 Nature (cells-test.gi.ucsc.edu/?ds=brain-vasc-atlas)
- Mathys et al., Cell 2023 (compbio.mit.edu/ad_aging_brain)
- Mathys et al., Cell 2023 (multi-region data; compbio.mit.edu/ad_aging_brain)
- Sun et al., Nature Neuroscience (compbio.mit.edu/scADbbb)
- Zhang et al., Medrxiv 2023 (GSE188545)

Research involving human participants, their data, or biological material

Policy information about studies with human participants or human data. See also policy information about sex, gender (identity/presentation), and sexual orientation and race, ethnicity and racism.

Reporting on sex and gender	We utilized sex as biological variable during analyses since sex was demonstrated to be a risk factor to develop and propagate Alzheimer's disease by some studies. Findings in this study were applicable to both sexes. We analyzed both sexes separately and collectively.
Reporting on race, ethnicity, or other socially relevant groupings	Samples were primarily collected from a brain bank and all samples were Non-Hispanic White. Samples in the ADNI and MCSA datasets are likewise predominantly NHW. During quality control steps in the analysis of genetic data associated with ADNI and MCSA datasets population outliers were removed using Eigenstrat.
Population characteristics	Age and sex matched individuals were selected for the snRNAseq study. 12 female and 12 male subjects that comprise matching number of Non-Hispanic White AD cases and controls were included. Both sexes cover broad range of neuropathology scores scores and risk factors such as age, and different APOE genotypes.<br><br>Age for control donors (Mean=86.16; Median= 88; Min=72; Max=98)<br>Age for AD donors (Mean=86.08; Median= 87; Min=73; Max=100)<br>APOE genotype for control donors (n numbers: APOE E2/3=1, APOE E3/3=8, APOE E3/4=3)<br>APOE genotype for AD donors (n numbers: APOE E3/3=5, APOE E3/4=5, APOE E4/4=2)<br>Braak stages for control donors (Mean= 2.1; Median= 2.0; n numbers: stage1 =2, stage2 =4, stage2.5 =1, stage3 =2)<br>Braak stages for AD donors (Mean= 5.5; Median=5.5; n numbers: stage5 =4, stage5.5 =3, stage6 =5)<br>Thal phases for control donors (Mean= 0.6; Median= 0.0; n numbers: phase0 =8, phase1 =1, phase3 =2)<br>Thal phases for AD donors (Mean= 4.4; Median= 5.0; n numbers: phase3=1, phase4 =8, phase5 =3)

Presence of TDP43 aggregates for control donors (n numbers: yes=3, no=6)
Presence of TDP43 aggregates for AD donors (n numbers: yes=7, no=5)
Presence of Lewy bodies for control donors (n numbers: yes=0, no=11)
Presence of Lewy bodies for AD donors (n numbers: yes=1, no=11)
Presence of Vascular dementia for control donors (n numbers: none=7, mild=4, moderate=1)
Presence of Vascular dementia for AD donors (n numbers: none=9, mild=2, moderate=1)
Brain weight at death for control donors (Mean= 1,315 grams; Median= 1,290 grams; Min=1,140 grams; Max= 1,700 grams)
Brain weight at death for AD donors (Mean= 1,125 grams; Median= 1,140 grams; Min=900 grams; Max= 1,240 grams)

Recruitment

Written informed consent was obtained from all participants or their qualified caregivers.

Ethics oversight

The study was approved by the Mayo Clinic institutional review board.

Note that full information on the approval of the study protocol must also be provided in the manuscript.

Field-specific reporting

Please select the one below that is the best fit for your research. If you are not sure, read the appropriate sections before making your selection.

☒ Life sciences ☐ Behavioural & social sciences ☐ Ecological, evolutionary & environmental sciences

For a reference copy of the document with all sections, see nature.com/documents/nr-reporting-summary-flat.pdf

Life sciences study design

All studies must disclose on these points even when the disclosure is negative.

Sample size

Postmortem: Frozen post-mortem brain tissues from 12 AD patients and 12 control donors, matched for age at death and sex, were obtained from the Mayo Clinic Brain Bank (MCBB). Sample size was determined by tissue availability of human frozen superior temporal gyrus. Selected cohort subjects that are assigned with neuropathology scores in broad range, and having different vulnerability levels against Alzheimer's disease. Braak neurofibrillary tangle (NFT) and Thal amyloid stages were assigned as previously described. Neuropathological assessment that comprises evaluation of gross and microscopic findings, as well as quantitative analysis of Alzheimer type pathology was conducted. Presence of TDP43 inclusion bodies were determined by immunohistochemistry with antibodies directed against pathological TDP43. Validation experiments contained the following number of subjects: 9 AD patients and 9 control donors from MCBB for RNAscope validation, 10 AD patients and 10 control donors from MCBB for immunohistochemical validation, 10 AD patients and 10 control donors from MCBB for qPCR validation, and 1 AD patient and 1 control donor from Columbia University Brain Bank for external immufluorescence validation.

Antemortem: Human participant sample size were determined by the number of available data. A total of 1,508 MCSA and 1,080 ADNI participants were analyzed for associations of infarcts with SMAD3 locus variants. A subset of 395 MCSA participants with PAXgene blood SMAD3 gene expression measures from RNAseq and 645 ADNI participants with PAXgene blood RNA expression were utilized for eQTL analysis. There were 638 participants from ADNI with blood PaxGene microarray SMAD3 expression, amyloid β (Aβ) positron emission tomography (PET) scans, and magnetic resonance imaging (MRI) scans.

IPSCs in vitro: Human participant sample size were determined by the availability of well-characterized AD and control donor derived IPSCs. Two AD patients and Two control donor derived IPSCs from Mayo Clinic Center for Regenerative Biotherapeutics were utilized. Independent from diagnosis background, IPSC were differentiated to pericytes and utilized for treatment experiments with VEGF, VEGFR2 inhibitor cocktail and aggregated Amyloid beta. Four IPSC lines were differentiated to pericytes in 9 different batches. Each treatment experiment was annotated with an identifier and summarized under Supplementary table S37. Effect of treatment was compared to medium change control. To perform statistical testing between non-treated and treated groups at a given treatment duration, we applied linear mixed effects model implemented in R package lmerTest. In the model, Treatment is the variable of primary interest, Treatment and Diagnosis are the fixed effects whereas Batch and Subject are the random effects.

Zebrafish in vivo: For single cells sequencing, SCOPIT was used to estimate sequencing depth and cell numbers. For experiments, effect and sample sizes were determined by G*Power and nQuery.

Data exclusions

None of the subjects described above were excluded from the relevant analyses.

Replication

Our main findings were successfully replicated using tiered approach. From human brain snRNAseq study, we discovered perturbed vascular and astrocytic transcript pairs, of which pericytic SMAD3 (up in AD) and astrocytic VEGFA (down in AD) were prioritized. In Vitro: We validated VEGFA-SMAD3 interactions in human iPSC-derived pericytes. Treatment of human pericytes with VEGF (encoded by VEGFA) reduces SMAD3, and blocking VEGF signalling increases SMAD3. In Vivo: To determine impact of VEGFA-SMAD3 interactions on the blood-brain-barrier experimentally, we utilized a well-established zebrafish model. Injection of amyloid beta-42 in this model decreased vegfaa (zebrafish ortholog to human VEGFA) expression in astroglia. Blocking vegfaa signaling pharmacologically increased phosphorylated Smad3, the active form of this signalling molecule and importantly also impaired blood-brain-barrier integrity.

Postmortem Measures: Age and sex matched AD and control donors were used in snRNAseq data generation (N=24). SMAD3 and VEGFA expression were validated independently from nuclei isolated on original 20/24 donors with sufficient tissue (Supplementary table S27). Each qPCR experiment contained three technical replicates for each gene expression. Expression of VEGFA in astrocytes and SMAD3 in vascular cells were validated in nuclei isolated original 20/24 donors with sufficient tissue via RNAscope (n=18, Supplementary tables S28 and S29). Total of 16 images were analyzed and all the number of cells were annotated for the marker expression. Immunohistochemistry experiments were performed in the TCX another cohort of AD and control donors from Mayo Clinic Brain Bank (N=20, Supplementary Table S30). each

staining experiment was performed independently from other donors. snRNAseq findings were validated using external datasets (Supplementary Table S31) independent.

Antemortem Measures: 2 independent study groups, ADNI and MCSA were used. For all analyses, we included relevant covariates such as APOE genotype, sex and batch in the model where appropriate. 5 Different SMAD3 probes were used to assess blood Paxgene expression. Correlation between SMAD3 probes as well as the average expression across all 5 probes is shown in Supplementary Figure S15.

IPSC Measures: 4 independent patient derived IPSCs were utilized in functional experiments. Each differentiating pericyte batch contained 6 technical replicates. There have been at least four repetitions of each experimental finding. Experimental design is displayed in Supplementary Figure S18.

Zebrafish Measures: Power analyses in zebrafish was performed using G*Power. At least 4 animals were used from both sexes as biological replicates. Multiple tissue sections were used per animal.

Randomization

Human postmortem: For human snRNAseq, randomization was performed such that the AD patients and control donors were matched for age at death and sex. Each batch or flowcell contained a balanced proportion of males/females and diagnosis groups.

Human antemortem: Variant dosages were tested for association with infarcts while adjusting for age (at time of neuroimaging), sex, batch and the first three principal components (PCs) accounting for population substructure.

Human IPSCs in vitro: All successfully differentiated pericytes were included in treatment experiments independent from diagnosis groups. We applied linear mixed effects model implemented in R package lmerTest. In the model, Treatment is the variable of primary interest, Treatment and Diagnosis are the fixed effects whereas Batch and Subject are the random effects.

Zebrafish in vivo: To minimize selection bias, zebrafish were randomly assigned to experimental and control groups to ensure that each fish has an equal chance of being placed in any group, making the groups comparable at the start of the experiment. In studies involving transgenic reporter lines, randomization was also applied to the selection of lines for experimentation. In experiments where there were known or potential confounding variables (e.g., age, sex, or batch effects in transgenic lines), a randomized block design was used. Zebrafish were grouped into blocks based on known variables, and then within each block, individuals were randomly assigned to experimental groups. Alongside randomization, samples were blinded to the group assignments, especially when evaluating outcomes. The person analyzing the results was different from the person conducting the experiments.

Blinding

Human postmortem: For the snRNAseq experiments, samples were randomized and were assigned a unique identifier. The investigators were blinded in the workflow except for nuclei sorting in snRNAseq data generation, where the diagnosis of the specimen was known. The investigator was not blinded for the analysis of the data.

Human antemortem: Bioinformatics personnel is blinded to overall experimental goals.

Human IPSCs in vitro: qPCR analysis were performed in a blinded fashion. IPSC maintenance, differentiation, and treatments were performed by an independent technician. RNA isolation and qPCR experimental data generation were performed by another technician. Data were analyzed and visualized by an independent investigator.

Zebrafish in vivo: Image analyses in zebrafish was performed in a blinded fashion. Tissue staining and labeling was performed by an investigator and IDs were revealed after quantification by another experimenter.

Reporting for specific materials, systems and methods

We require information from authors about some types of materials, experimental systems and methods used in many studies. Here, indicate whether each material, system or method listed is relevant to your study. If you are not sure if a list item applies to your research, read the appropriate section before selecting a response.

Materials & experimental systems

n/a	Involved in the study	
☐	☒	Antibodies
☐	☒	Eukaryotic cell lines
☒	☐	Palaeontology and archaeology
☐	☒	Animals and other organisms
☒	☐	Clinical data
☒	☐	Dual use research of concern
☒	☐	Plants

Methods

n/a	Involved in the study	
☒	☐	ChIP-seq
☐	☒	Flow cytometry
☐	☒	MRI-based neuroimaging

Antibodies

Antibodies used

Postmortem-SnRNAseq data Generation: anti-Human Nuclear Antigen (HNA) [235-1] (ab191181, Abcam, 1:50), Mouse IgG1, kappa monoclonal [15-6E10A7] isotype control (ab170190, Abcam, 1:50), and goat anti-mouse Alexa488 secondary antibody (ab150113, Abcam, 1:200).
Postmortem-Nuclei Purity Validation Nuclear H3 (Abcam, Y47, ab32356, 1/200) and mitochondrial COX4 (Abcam, mAbcam33985, ab62164, 1/200).
Postmortem - Immunohistochemistry: phospho-SMAD3 antibody (Thermo Fisher, S.434.0, MA5-14936, 1/100).

	Postmortem - Immunofluorescence: SMAD3 (Thermo Fisher, E.980.9, MA5-14939, 1:500), PDGFRB (Thermo Fisher, PR7212, MA5-28128, 1:500), VEGFA (R&D Biosystems, VG1, MAB2932-100, 1:500), and GFAP (Thermo Fisher, OPA1-06100, 1:500).<br>In vitro- IPSC Differentiation validation: Anti-NG2 (BD Pharmingen, 554275, Clone 9.2.27, 1/300), Anti-PDGFRB (R&D Systems, MAB1263, PR7212, 1/300) and Anti TRA1-60 (Abcam, ab16288, 1/300).<br>In vitro - Immunocytochemistry: Anti-PDGFRB (R&D Systems, MAB1263, PR7212, 1/100) and Anti-Actin (Thermo Fisher, MA511869, ACTN05 (C4), 1/200)<br>In vivo - Immunohistochemistry: chicken anti-GFP (Thermofisher, PA1-9533, 1:1000), rabbit anti-phospho-ERK (Cell Signaling, 9101, 1:500), rabbit anti-phospho-SMAD3 (Abcam, EP823Y, ab52903, 1:500), mouse anti-ZO-1 (Thermofisher, ZO1-1A12, 33-9100, 1:500).
Validation	Antibodies are validated by the manufacturer through wither western blots, immunization peptide inhibition or knockout validation. All antibodies were tested for efficacy and reliability with no-primary controls in human samples, cell cultures,and zebrafish before use.

Eukaryotic cell lines

Policy information about cell lines and Sex and Gender in Research

Cell line source(s)	Two fully characterized AD- and two control patient-derived iPSCs were kindly provided by Mayo Clinic Center for Regenerative Biotherapeutics (Supplementary table 36).
Authentication	We used already authenticated patient derived IPSC cells (Supplementary Table 36). We validated the pluripotency and ectodermal differentiating capability of IPSCs through immunocytochemistry. Details are shown in Supplementary Figure S16.
Mycoplasma contamination	Mycoplasma contamination was analyzed through optimized and validated with MycoAlert® PLUS Mycoplasma Detection Kit (Lonza, LT07-710). IPSCs were validated for the absence of mycoplasma contamination prior to pericyte differentiation.
Commonly misidentified lines<br>(See ICLAC register)	This study did not involve commonly misidentified cell lines.

Animals and other research organisms

Policy information about studies involving animals; ARRIVE guidelines recommended for reporting animal research, and Sex and Gender in Research

Laboratory animals	Double reporter transgenic zebrafish line: Tg(her4:DsRed) and Tg(fli1a:eGFP). Single reporter transgenic zebrafish line: Tg(kdrl:GFP). Background strains were AB. 6 months old animals were used.
Wild animals	This study did not involve wild animals.
Reporting on sex	Equal number of animals from both sex groups were included in the analysis. In every experimental set, animals from the same fish clutch were randomly distributed for each experimental condition.
Field-collected samples	This study did not involve field-collected samples.
Ethics oversight	Animals are maintained according to the Institutional Animal Care and Use Committee (IACUC) standards of the Institute of Comparative Medicine at the Columbia University Irving Medical Center and to the accepted guidelines. The animal care and use program at Columbia University is accredited by the AAALAC International and maintains an Animal Welfare Assurance with the Public Health Service (PHS), Assurance number D16-00003 (A3007-01). Animal experiments were approved by the IACUC at Columbia University (protocol number AC-AABN3554). 3R principles applied throughout the study.

Note that full information on the approval of the study protocol must also be provided in the manuscript.

Flow Cytometry

Plots

Confirm that:

☒ The axis labels state the marker and fluorochrome used (e.g. CD4-FITC).

☒ The axis scales are clearly visible. Include numbers along axes only for bottom left plot of group (a 'group' is an analysis of identical markers).

☒ All plots are contour plots with outliers or pseudocolor plots.

☐ A numerical value for number of cells or percentage (with statistics) is provided.

Methodology

Sample preparation	Nuclei Isolation and Purification from Frozen Human Brain: Temporal cortex tissue samples were obtained from the Mayo Clinic Brain Bank. Total RNA from ~20 mg collected tissue was isolated to evaluate the quality of tissue. RNA integrity number (RIN) was determined via Agilent 2100 Bioanalyzer using RNA Pico Chip assay, and tissues that have RIN > 6.0 were utilized in nuclei isolation and single nucleus RNA sequencing (snRNAseq). For each participant, 100 mg tissue sample was used for nuclei isolation using a modified protocol. Samples were homogenized with 25 strokes of loose and tight pestle sequentially using dounce homogenizer in homogenization buffer (0.25 M sucrose, 25 mM KCl, 5 mM MgCl2, 20 mM tricine-KOH, pH 7.8,

1 mM DTT, 0.15 mM spermine, 0.5 mM spermidine, protease inhibitors, 5 μ g/mL actinomycin, 5 u/ μL recombinant RNAase inhibitor, and 0.04% BSA). IGEPAL (5%, Sigma, I8896) solution was added following stroke with the tight pestle to a final concentration of 0.32%. After 10 additional strokes, the tissue homogenate was filtered using a 30 μm cell strainer. Debris was pelleted by centrifugation at 500g for 5 minutes and washed with Wash and Storage Buffer (WSB, 1XPBS with 2%BSA and 5 u/ μL recombinant RNAase inhibitor (Takara Bio, 2313A)). The nuclei-containing supernatant was filtered again with a 30 μm cell strainer, followed by centrifugation at 500g for 10 minutes. After re-suspending the pallet in 700μl cold PBS with 5 U/μl RNAse inhibitors, 300μl debris removal solution (Miltenyi Biotech) was added, and the solution was gently mixed. Another 1 mL WSB was carefully overlaid on top of the nuclei solution. The supernatant was removed after centrifugation at 3000g for 10 minutes. The nuclei were washed once with WSB and pelleted after centrifugation for 10 minutes at 1000g. Isolated nuclei were sorted using fluorescence-activated nuclei sorting (FANS). Human Nuclear Antigen [235-1] (ab191181, Abcam) antibody was applied to the nuclei at 1:50 and incubated for 1 hour on ice. Concurrently, mouse IgG1, kappa monoclonal [15-6E10A7] isotype was included as controls (ab170190, Abcam, 1:50). Goat anti-mouse Alexa488 secondary antibodies (ab150113, Abcam, 1:200) were incubated with the nuclei for 30 minutes on ice. The stained nuclei were reconstituted in WSB and sorted using BD FACSAria II sorter using the 70-micron nozzle with 70 psi sheath pressure and 1.5 ND filter.

In vitro - IPSC Validation: Two fully characterized AD- and two control patient-derived iPSCs were kindly provided by Mayo Clinic Center for Regenerative Biotherapeutics (Supplementary table 36). These cells were fully characterized previously80,81,83 and validated for pluripotency and ectodermal differentiation capability (Supplementary Figure 16). iPSCs were maintained in mTesR1 medium (Stem Cell Technologies, 100-0276) on Matrigel (Corning, 354277) coated plates. All iPSCs were passaged when the lines reached 70 % confluency by either manual selection of healthy colonies or ReLeSR (StemCell Technologies, 05872). Pericyte differentiation was adapted from previous studies and applied with slight modifications79. Prior to differentiation, iPSCs were passaged with Accutase (StemCell Technologies, 7920) and plated onto 6-well plates with mTeSR1 medium supplemented with 10 μM Rock Inhibitor Y27632 (Stem Cell Technologies, 72302) and plated at a density of 40,000 cells/cm2. On the first day, the cells were washed with 1x PBS and maintained in differentiation N2B27 medium (1:1 DMEM/F12 + neurobasal medium, B27 and N2 supplements, penicillin, and streptomycin, and Beta-Mercaptoethanol) supplemented with 25 ng/mL of BMP4 (R&D Biosystems, 314-BP-050) and 8 uM CHIR99021 (R&D Biosystems, 4423/10) for three days. On both days 4 and 5, medium was changed to fresh N2B27 media supplemented with 2 ng/mL of Activin A (R&D Biosystems, 338-AC-010) and 10 ng/mL PDGF-BB (Stem Cell Technologies, 78097). On day 6, pericytes were passaged with Accutase, plated onto a new matrigel coated plate at a density of 18000 cells/cm2, and cultured in N2B27 medium for 6 days. Medium is changed in every 2 days. On day 12, pericytes were passaged via Accutase and seeded for treatment experiments onto 24-well plates with the density of 50,000 cells/well. Pericytes were incubated with primary antibodies Anti-NG2 (BD Pharmingen, 554275, Clone 9.2.27, 1/300), Anti-PDGFRB (R&D Systems, MAB1263, PR7212, 1/300) and Anti TRA1-60 (Abcam, ab16288, 1/300) in blocking buffer (1x PBS, 0.5% BSA, 2% FBS, and 3 mM EDTA) for 1 hour on ice. Cells were incubated for 30 minutes on ice in secondary antibody solution that contains goat anti-mouse Alexa488 secondary antibody (Abcam, ab150113, 1/200). 7-AAD (Sigma, A1310, 1/100) was used to stain live/dead cells.

In vivo - Reporter Cell Isolation and Purification from Amyloid Beta 42 Injected Zebrafish Brain: Amyloid beta toxicity was induced by Cerebroventricular injection as previously described (https://doi.org/10.1371/journal.pone.0027395). The brains were dissected, and single cell suspensions were prepared according to previously established method, which is depicted under https://doi.org/10.1016/j.xpro.2020.100042.

Instrument

Mayo BD FACS Aria II (BD Biosciences), Mayo Attune NxT Flow cytometer (Life Technologies), and Columbia BD FACS Aria II (BD Biosciences)

Software

BD FACSDiva software (BD Biosciences) were used to sort gated population on FACS Aria. FCS files were acquired through Attune NxT Flow cytometer (Life Technologies) and processed and gated in FlowJo (BD Biosciences, v10).

Cell population abundance

Postmortem - Population: 79,751 cells were sequenced and passed the quality filter in human brain snRNAseq data.
In vivo - Population: 22,396 cells were sequenced and passed the quality filter in zebrafish brain scRNAseq data.

Gating strategy

Postmortem Gating and Sorting Strategy: FSC-A/SSC-A gates were set to select nuclear fraction. SSC-H/SSC-W and FSC-H/FSC-W gates were applied to nuclear fraction to discriminate doublets. Secondary antibody stained nuclei control sample were gated for HNA+ in FITC-H/SSC-H channel. This gate was applied to anti-HNA stained sample and sorting was performed. Sorted nuclear sample is run again FACS Aria II to validated the sorting and determine the efficiency of the sorting. Sorting strategy is depicted in Supplementary Figure S25.

In vitro Gating Strategy: FSC-A/SSC-A gates were set to select cell fraction. FSC-H/FSC-A gate was applied to cell fraction to discriminate doublets. Secondary antibody stained cells were gated for HNA+ in FITC-H/SSC-H channel. This gate was applied to anti-HNA stained sample and sorting was performed. The shift in the population is calculated.

In vivo Gating and Sorting Strategy: Cytox-Blue negative (viable) and DyeCycle Ruby positive (proliferating) cell population were gated for cell viability. FSC-A/SSC-A gates were set to select cellular fraction. FSC-H/FSC-A gate was applied to cell fraction to discriminate doublets. Two seperate gates were applied to sort transgenic cells into two seperate tubes: GFP+/dsRED- and GFP-/dsRED+ fractions. Sorting strategy is depicted in Supplementary Figure S22.

☒ Tick this box to confirm that a figure exemplifying the gating strategy is provided in the Supplementary Information.

Magnetic resonance imaging

Experimental design

Design type	n/a
Design specifications	n/a
Behavioral performance measures	n/a

Acquisition

Imaging type(s)	Structural
Field strength	1.5 Tesla and 3 Tesla
Sequence & imaging parameters	MPRAGE or comparable T1-weighted volumetric scan
Area of acquisition	Whole brain

Diffusion MRI ☐ Used ☒ Not used

Preprocessing

Preprocessing software	FreeSurfer V5.1
Normalization	Non-linear transformation of MRI scans to normal atlas space
Normalization template	MNI
Noise and artifact removal	All data undergoes visual QC to confirm segmentation and parcellation. Scans with poor Freesurfer segmentations/ parcellations due to noise, movement, or other artifact are excluded from the sample for imaging comparisons.
Volume censoring	None

Statistical modeling & inference

Model type and settings	Multivariable linear regression
Effect(s) tested	gene expression levels

Specify type of analysis: ☒ Whole brain ☐ ROI-based ☐ Both

Statistic type for inference (See Eklund et al. 2016)	Cluster-wise $p<0.05$
Correction	Random field theory (RFT) correction

Models & analysis

n/a	Involved in the study
☒	☐ Functional and/or effective connectivity
☒	☐ Graph analysis
☐	☒ Multivariate modeling or predictive analysis

Multivariate modeling and predictive analysis	Effect of blood SMAD3 gene expression levels on vertex-wise cortical thickness covaried for age, sex, years of education, MRI field strength, and total intracranial volume
